# Comparative Proteomics of Inner Membrane Fraction from Carbapenem-Resistant *Acinetobacter baumannii* with a Reference Strain

**DOI:** 10.1371/journal.pone.0039451

**Published:** 2012-06-26

**Authors:** Vishvanath Tiwari, Jitendraa Vashistt, Arti Kapil, Rajeswari R. Moganty

**Affiliations:** 1 Department of Biochemistry, All India Institute of Medical Sciences, Ansari Nagar, New Delhi, India; 2 Department of Microbiology, All India Institute of Medical Sciences, Ansari Nagar, New Delhi, India; University of Minho, Portugal

## Abstract

*Acinetobacter baumannii* has been identified by the Infectious Diseases Society of America as one of the six pathogens that cause majority of hospital infections. Increased resistance of *A.* *baumannii* even to the latest generation of β-lactams like carbapenem is an immediate threat to mankind. As inner-membrane fraction plays a significant role in survival of *A.* *baumannii,* we investigated the inner-membrane fraction proteome of carbapenem-resistant strain of *A.* *baumannii* using Differential In-Gel Electrophoresis (DIGE) followed by DeCyder, Progenesis and LC-MS/MS analysis. We identified 19 over-expressed and 4 down-regulated proteins (fold change>2, *p*<0.05) in resistant strain as compared to reference strain. Some of the upregulated proteins in resistant strain and their association with carbapenem resistance in *A.* *baumannii* are: i) β-lactamases, AmpC and OXA-51: cleave and inactivate carbapenem ii) metabolic enzymes, ATP synthase, malate dehydrogenase and 2-oxoglutarate dehydrogenase: help in increased energy production for the survival and iii) elongation factor Tu and ribosomal proteins: help in the overall protein production. Further, entry of carbapenem perhaps is limited by controlled production of OmpW and low levels of surface antigen help to evade host defence mechanism in developing resistance in *A.* *baumannii*. Present results support a model for the importance of proteins of inner-membrane fraction and their synergistic effect in the mediation of resistance of *A.* *baumannii* to carbapenem.

## Introduction

*Acinetobacter baumannii* is a non-motile, Gram negative bacteria known to cause a number of hospital-acquired (nosocomial) infections including pneumonia, urinary tract infections particularly, amongst patients in the intensive care units, neonatal units and neurosurgical wards. Infections caused by *A.* *baumannii,* have increased substantially in the last decade and account for about 10% of total bacterial infections [1]–[4]. However, in India, prevalence of *A.* *baumannii* is about 20%, making it one of the most notorious gram negative bacteria [5]. The alarming rate (26%) at which *A.* *baumannii* is gradually increasing is of great concern [6]. Infections caused by *A.* *baumannii* represent an important source of morbidity, mortality and increased costs [7], [8], [1].

*A.* *baumannii* has acquired resistance to most of these antibiotics all over the world which is a potential hazard in the treatment. In other words, the availability of effective antibiotics to treat *A.* *baumannii* is restricted due to rapid increase in the drug resistance of *A.* *baumannii* [9], [1], [2]. Carbapenems including imipenem, meropenem and doripenem were identified as alternate therapeutics for *A.* *baumannii* and are still the most important options for serious infections caused by multidrug-resistant *A.* *baumannii*. However, by using versatile and robust mechanisms, *A.* *baumannii* acquired resistance even to the latest carbapenems. This can be understood by looking at the resistant rate to carbapenem which was only 2% in early 1990’s has increased to 71% by 2008 [10] and is still increasing. Therefore, *A*. *baumannii* infections are increasingly becoming difficult to eradicate due to high-level of resistance as a result of both intrinsic and acquired mechanisms.

*A.* *baumannii* is known to utilise and activate a number of mechanisms in developing resistance which include, altering outer membrane proteins (to decrease the permeability), increasing production of β-lactamases (to hydrolyze β-lactam), alterations in penicillin binding proteins (to facilitate cell wall synthesis) and activate production of efflux pumps [11]–[16]. It has been reported that antibiotic resistance in *A.* *baumannii* is highly associated with membrane proteins [17]. Differential production of membrane proteins in susceptible and highly resistant strains of *A.* *baumannii* from different parts of the world clearly shows its strong association with the emergence of the resistance phenotype [17]–[20]. Inner membrane fraction proteins (IMFPs) are essential for energy production, metabolic activities and cell signalling etc. Most of the studies carried out on membrane proteomics of *A.* *baumannii* focused on the outer membrane [21], [11]. However, laboratory/artificially induced imipenem resistance was studied by Yun et al in plasma membrane of DU202 strain [20] and Siroy et al performed inner membrane proteomics on resistant strain of *A.* *baumannii* using conventional 2D electrophoresis [17]. However, there are no report available on IMFPs of clinical isolates from hospital using Differential In-Gel Electrophoresis (DIGE), a highly sensitive fluorescence based method. Therefore, present study is an attempt to identify differently expressed IMF proteins of *A.* *baumannii* in three clinical isolates (with different resistance levels) from our hospital by using DIGE-based proteomic approach.

## Materials and Methods

### Reagents

MacConkey agar and Muller Hinton agar were purchased from Himedia Laboratories Ltd., India and LB media was from Pronadisa Laboratories, Spain. Urea, thiourea, Tris-HCl, NaCl and glycine were from Merck, India; *N*-lauroyl-sarcosine and ammonium bicarbonate (mass spectrometry grade) were from Sigma Chemical Co., U.S.A. Hydrochloric acid, glacial acetic acid, glycerol, and methanol were from Qualigens, India. Acrylamide, bisacrylamide, ammonium persulphate, TEMED, SDS, EDTA, Coomassie Brilliant Blue, β-mercaptoethanol and bromophenol blue were obtained from Bio-Rad Laboratories, U.S.A. Mass spectrometry grade trypsin was from Promega, U.S.A. Immobiline dry strips, pharmalytes, dry strip cover fluid, dithiothreitol (DTT), iodoacetamide and fluorescent dyes (Cy2, Cy3 and Cy5) were purchased from GE Health Care, Singapore. Dimethylformamide (HPLC grade) was from Spectrochem, India. CHAPS, HPLC grade acetonitrile (ACN) and proteomic grade water were purchased from G. Biosciences, U.S.A. All other routine chemicals obtained from Merck, India, were of analytical grade.

**Table 1:** Selected protein spots from comparative analysis of differentially expressed proteins in DeCyder and Progenesis software.

S.No.	Spot number in DeCyder (Progenesis)	Fold change In DeCyder (ANOVA)	Fold change in Progenesis (ANOVA)	Differential Expression
1	490 (213)	7.23 (0.00018)	2.6(0.005)	Upregulated
2	904(417)	7.14 (0.00032)	3.4(0.019)	Upregulated
3	902 (416)	3.75 (0.00055)	2.7(0.013)	Upregulated
4	603 (267)	5.34 (0.0015)	3.7(0.012)	Upregulated
5	455(187)	4.95 (0.0017)	3.4(0.001)	Upregulated
6	595(265)	3.51 (0.0022)	2.3(0.010)	Upregulated
7	428(173)	3.01 (0.0029)	2.9(0.014)	Upregulated
8	489 (212)	4.20 (0.0041)	2.2(0.003)	Upregulated
9	436 (178)	5.48 (0.0048)	2.5(0.030)	Upregulated
10	365 (158)	3.68 (0.0050)	3.7(0.009)	Upregulated
11	366 (153)	3.46 (0.0070)	2.7(0.047)	Upregulated
12	327 (107)	2.96 (0.0082)	3.0(0.005)	Upregulated
13	432 (176)	3.35 (0.0092)	2.9(0.005)	Upregulated
14	434 (180)	3.49 (0.028)	2.5(0.011)	Upregulated
15	1012 (ND)	4.90 (0.00067)	ND	Upregulated
16	745 (ND)	2.03 (0.0065)	ND	Upregulated
17	1089 (ND)	2.96 (0.0010)	ND	Upregulated
18	645 (ND)	6.50 (0.0017)	ND	Upregulated
19	650 (ND)	3.74 (0.0010)	ND	Upregulated
20	945 (420)	−6.85 (0.0050)	−3.4(0.012)	Downregulated
21	1004 (465)	−6.28 (0.0053)	−7.2(0.002)	Downregulated
22	1266(579)	−6.37 (0.0054)	−2.6(0.017)	Downregulated
23	1269(582)	−4.79 (0.0077)	−2.8(0.010)	Downregulated

ND =  Not Detected in Progenesis with significant p value <0.05 and fold change >2.

### Bacterial Strains and Minimal Inhibitory Concentration Determination

The reference (susceptible) strain ATCC 19606 and 25 clinical strains of *A.* *baumannii* were collected from the Department of Microbiology, All India Institute of Medical Sciences, New Delhi. Various biochemical tests like Gram staining, catalase test, citrate test, triple sugar iron agar test, urease test, motility test, indole test and temperature sensitive test were used for confirmation of strains of *A.* *baumannii* for the present study [22]. The Minimal inhibitory concentrations of ATCC and 25 clinical strains of *A.* *baumannii* were determined for imipenem. ATCC19606 and three carbapenem resistant strains (high resistant RS307, intermediate resistant RS122 and low resistant RS259) of *A.* *baumannii* were selected for present study.

**Figure 1:**
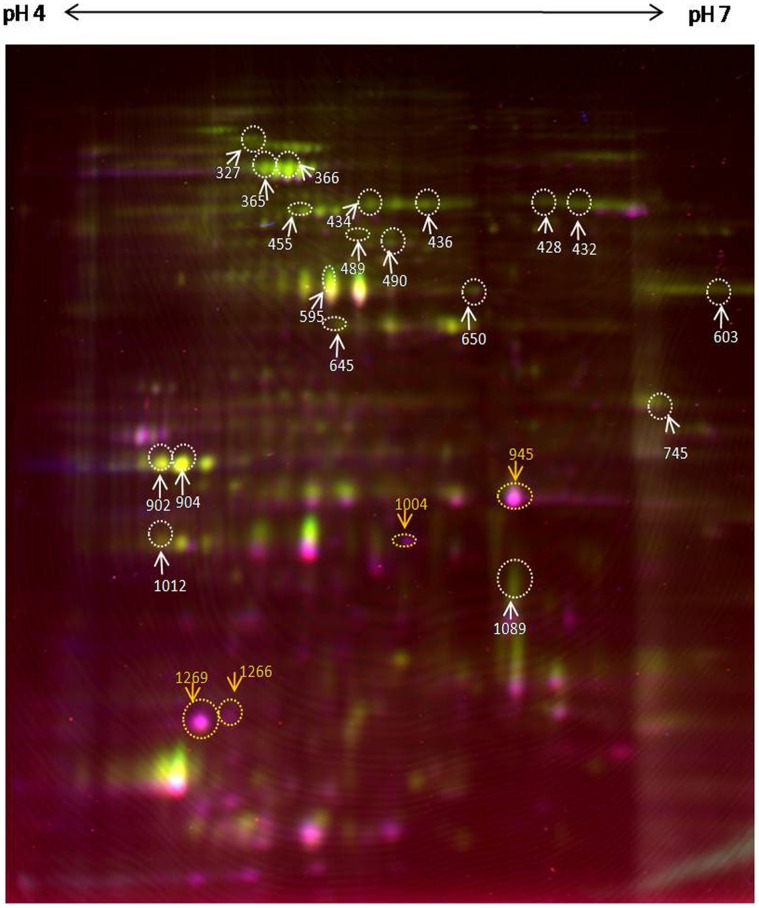
Differential In-Gel Electrophoresis of inner membrane fraction of *Acinetobacter baumannii* ATCC19606 and resistant strain RS307. Up-facing white arrow: Upregulated proteins; Down-facing yellow arrow: Down-regulated proteins.

### Inner Membrane Faction Proteins (IMFPs) Extraction

Total membrane proteins were extracted according to our previously described method [11]. The pellet containing the total membrane fraction was washed and resuspended in 2% Sarkosyl buffer (*N-*laurylsarkosine in Tris-HCl, pH 7.5**)** which solubilise the inner membrane [23]. After sarkosyl treatment, sample was subjected to ultracentrifugation at 100,000 *g* for 30 min. The inner membrane fraction proteins (IMFPs) were separated out as supernatant which contain “inner membrane proteins” and “periplasmic proteins” and they were stored at −70°C. However, it may be mentioned that a small protein fractions in IMFPs may be derived from cytoplasm and outer membrane which is unavoidable [24]. ATCC19606, RS307, RS122 and RS259 of *A.* *baumannii* were grown three times independently under conditions explained above and its IMFPs were extracted and stored at −70°C.

### Differential In-Gel Electrophoresis (DIGE)

50 µg of total IMFPs either from native or RS307 was labeled with the 200 pmol of fluorescent dyes Cy3 or Cy5 separately. One-sixth fraction of protein sample from each strain was pooled and labeled with Cy2. Proteins labeled with Cy2 acts as internal standard [25]. Protein labeling was performed using similar method published in our previous paper [11]. The final volume of reaction mixture was adjusted to 250 µL using rehydration buffer (7 M urea, 2 M thio-urea, 2% CHAPS, 0.7 mg of DTT, and 1.25 µL of IPG buffer). Rehydration was performed for 16 h in the dark with 13 cm, 4–7 pH IPG strip. Similar experiments of DIGE were also performed with ATCC and RS122 or RS259.

### Two-Dimensional Gel Electrophoresis

In 2D gel electrophoresis, isoelectric focusing (IEF) was done on Ettan IPGphor 3 IEF system and SDS-PAGE analysis were carried out in SE 600 Ruby gel apparatus (GE Healthcare). Rehydrated IPG strip was subjected to isoelectric focusing for 25000 VhT. After IEF, strips were equilibrated in 2.5 mL of SDS-equilibration buffer (50 mM Tris-HCl (pH 8.8), 6 M urea,30% glycerol, 2% SDS, and 0.02% bromophenol blue) containing first 0.05% DTT for 15 minute and then with SDS-equilibration buffer containing 1.25% iodoacetamide for 15 minute. Second-dimension electrophoresis was run on a 12% polyacrylamide gel at 4°C with 15 mA for 30 min and then at 30 mA for both the strips, until the bromophenol blue came out of the gel.

**Table 2:** Identification of differentially expressed proteins of inner membrane fraction of *Acinetobacter baumannii* resistant strain, RS307 with reference to ATCC 19606.

Master No.	Mw kDa	pI	Identification and Accession No.	Score/% coverage	Possible Role in Resistance
**Up-regulated Proteins**					
**β-Lactamases**					
603	43.2	9.49	Beta-lactamase class C [*Acinetobacter baumannii*]2::gi|7258342	156/10	AmpC, the major lactamase of class C cleaves antibiotics.
745	30.6	8.43	Carbapenem-hydrolyzing oxacillinase OXA-71[*Acinetobacter baumannii*], gi|57491188	127/8	OXA-71 belongs to OXA-51 oxacillinase and cleaves oxacillin and other carbapenem.
**Proteins associated with metabolism and energy production**					
455	50.3	5.03	ATP synthase subunit beta OS = *Acinetobacter* *baumannii* (strain ACICU) 1::ATPB_ACIBC	860/48	Make resistant strain more energy efficient by ATP production.
436 and 434	55.5	5.29	ATP synthase subunit alpha OS = *Acinetobacter* *baumannii* (strain ACICU) 1::ATPA_ACIBC	371/16	Maintain functional conformation of ATP synthase and help beta subunit to produce ATP
428	42.5	5.64	Dihydrolipoamide dehydrogenase [*Acinetobacter* *baumannii* ATCC 17978] 2::gi|126642748	63/7	Helps with addition energy requirements by producing acetyl COA.
645	35.4	5.20	Malate dehydrogenase OS = *Acinetobacter* *baumannii* 1::MDH_ACIBC	316/21	Involved in adaptation during oxidative stress and also in biofilm formation.
432	51.2	5.96	2-oxoglutarate dehydrogenase complex[*Acinetobacter* sp. RUH2624] 2::gi|260549007	276/15	Associated with higher oxygen transport and efficient energy production in resistant strain.
650	38.6	5.27	Coproporphyrinogen III oxidase [*Acinetobacter* *baumannii* SDF]	90/6	Enzyme involved in the porphyrin metabolism, but its specific role in resistance is unknown.
**Protein synthesis machinery**					
1089	18.0	5.62	50 S Ribosomal protein of *Acinetobacter* *baumannii* RL10_ACIB3	151/16	It activates protein translation to take care of the increased metabolic requirements.
327	61.3	4.89	30 S ribosomal protein 1::RS1_ECO57	117/2	Elevated ribosomal proteins facilitate carbapenem resistant.
490	43.2	5.20	Elongation factor Tu [*Acinetobacter baumannii*]1::EFTU_ACIBT	138/11	Up regulation of EF-Tu is the consequent event of ribosome upregulation; promotes translation.
489	43.2	5.20	Elongation factor Tu OS = *Acinetobacter* sp.(strain ADP1) 1::EFTU_ACIAD	162/8	Same as above
595	37.3	5.10	DNA-directed RNA polymerase subunit alphaOS = *Acinetobacter baumannii*. RPOA_ACIBC	199/14	Activate transcription and thereby protein synthesis under the antibiotic stress.

Differential expression is shown as fold change (minimum 2 fold, p-value e0.05).

### Image Acquisition

DIGE gels were scanned for Cy2, Cy3 and Cy5 fluorescence labeled proteins using a Typhoon TRIO Variable Mode Imager (GE Healthcare). Cy2 images were scanned using 488 nm excitation and 520BP40 emission filter; Cy3 images were scanned using 532 nm excitation and 580BP30 emission filter; Cy5 images were scanned using 633 nm excitation and 670BP30 emission filter. All gels were scanned with a PMT of 600. Images were cropped using Image-Quant version 7.0 (GE Healthcare) to remove extra areas to the gel image. The final protein levels were determined by the DeCyder software version 7.0 (GE Healthcare) and Progenesis Same Spots v3.2 (Nonlinear Dynamics).

### Statistical Analysis

Replicate gels were used to calculate average abundance differences and Student’s *t*-test *p*-values for each protein across the three replica gels.

**Table 3:** Identification of differentially expressed proteins of inner membrane fraction of *Acinetobacter baumannii* resistant strain, RS307 with reference to ATCC 19606.

Master No.	Mw kDa	pI	Identification and Accession No.	Score/% coverage	Possible Role in Resistance
**Up-regulated Proteins**					
**Chaperonins**					
365	57.2	4.92	60 kDa chaperonin OS = *Acinetobacter baumannii* (strain AB307-0294) 1::CH60_ACIB3	191/11	Maintain newly translated protein in the correctly folded form.
366	31.7	4.74	TCP-1/cpn60 chaperonin family protein 2::gi|254480295	62/20	Involved in stress induced stabilisation of protein. It also protects disassembled polypeptides under heat-shock conditions.
**Transporter**					
1012	16.4	4.79	Putative lipoprotein [*Acinetobacter baumannii* SDF] 2::gi|169633901	236/36	Lipoproteins have been implicated in the adhesion and translocation of virulence factors in host cells.
**Unknown function**					
902 and 904	26.4	4.79	Carbapenem-associated resistance protein precursor [*Acinetobacter baumannii*] 2::gi|56131242	283/19	Known to be involved in carbapenem resistance- direct function is unknown
**Down-regulated Proteins**					
1266 and 1269	11.3	4.77	Surface antigen [*Acinetobacter baumannii* ATCC 17978] 2::gi|126641429	75/11	Evade host defense mechanism and decrease cellular recognition by host.
945	20.7	5.66	Ribosome-recycling factor OS = *Acinetobacter baumannii* (strain SDF) RRF_ACIBT	75/8	Associated with increase in the efficiency of translation.
1004	21.2	5.56	Putative outer membrane protein W [*Acinetobacter baumannii* ] 2::gi|239500971	53/10	Decreased entry of antibiotic.

Differential expression is shown as fold change (minimum 2 fold, p-value e0.05).

#### DeCyder

Gel images were processed by DeCyder Differential Analysis (DIA) using default setting. The estimated number of spots for each co-detection procedure was set to 1500. The spots on gels were co-detected automatically as 2DE DIGE image pairs, which intrinsically link a sample to its in-gel standard. Differential protein production between two samples in the gel was based on the ratio of standardized log abundance of the Cy3 versus Cy5 spot volume over the Cy2 spot volume. Initially, all DIA workspaces were imported to Biological Variance Analysis (BVA) workspace and the experimental setup and relationship between samples were assigned in the BVA workspace. Each individual image of Cy3 gel or Cy5 gel was assigned an experimental condition, either native ATCC or resistant strain according to the labeling. All Cy2 images were classified as internal standards and were used for gel-to-gel matching of the standard spot maps in all gel images. The gel with the highest spot count was assigned as the master gel. Matching between gels was performed utilizing the in-gel standard from each image pair. Matching was further improved by land marking and manually confirming potential spots of interest. The degree of difference in standardized abundance between two protein spot groups is expressed as average ratio (fold change). A fold change with a threshold value of minimum 2-fold increase or decrease was used. Student *t* test was performed for every matched spot-set, comparing the average and standard deviation of protein abundance for a given spot. Therefore, proteins which had higher value than 2-fold change and also with a significant *p*-value (e0.05) were considered in the present study for identification [26], [27].

#### Progenesis same spots

The gel image analysis was also performed using Progenesis Same Spots v3.2 image analysis software. The image realigning, noise filtering, and spots segmentation were carried out using default setting as outlined in the software manual [28], [29]. Automatic analysis was performed on all the aligned images using the analysis wizard. The aligned images were grouped into ATCC and resistant strain to reflect the biological grouping and the statistically ranked list of spots were evaluated in the review stage of the software package [28]. A total of 1200 protein spots were identified across all the samples. Each spot volume was normalized using the ratiometric method in Progenesis. The logarithm form of the normalized spot volume was taken. Differentially expressed protein spots were determined using analysis of variance (ANOVA) across the two groups (ATCC and resistant strain). The protein spots with a p-value ≤0.05 were considered as significant and those spots were used for subsequent study [29].

### In-Gel Digestion and Peptide Extraction

Protein spots were excised and in-gel digestion was carried out using trypsin (mass spectrometry grade, Promega Corp., Madison,WI) and peptide were extracted according to manufacture protocol.

### Liquid Chromatography and Electrospray Ionization Mass Spectrometry (LC-MS/MS)

After digestion, tryptic peptides were analyzed by LC-MS/MS. The resulting peptide mixture was separated by reverse phase chromatography (TempoTM nano-LC system, Applied Biosystems) using a Pep Map C18 column. Peptides were separated using a 70 minute linear gradient from 5% to 98% acetonitrile in 0.1% formic acid with a flow rate of 400 nl/min. The eluting peptides were ionized by electrospray ionization (ESI) and analysed by QSTAR XL system (Applied Biosystems, USA). Nanospray ionization was carried out using an ion spray voltage of 900. The progress of each run was monitored by recording the total ion current (TIC) for positive ions as a function of time in the m/z range of 400–1600 for MS and 140–1600 for MS/MS. The spectra was acquired in an information dependent manner utilizing the Analyst QS 2.0 software acquisition features to generate raw data in the *.wiff format. The other parameters set were: interface temperature, 50°C; curtain gas flow, 1.13 L/min; declustering potential, 60 V; focusing potential, 280 V; declustering potential 2, 15 V. Database searching was done using Mascot (Matrix Science, U.K., www.matrixscience.com). Different parameters were selected during Mascot searching are modifications of methionine (oxidation) and cysteine (carbamidomethylation), charged state ranging from +1, +2, +3 and search limited to Eubacteria, peptide mass tolerance range of 1.2 Da and fragment mass tolerance range of 0.8 Da.

**Figure 2:**
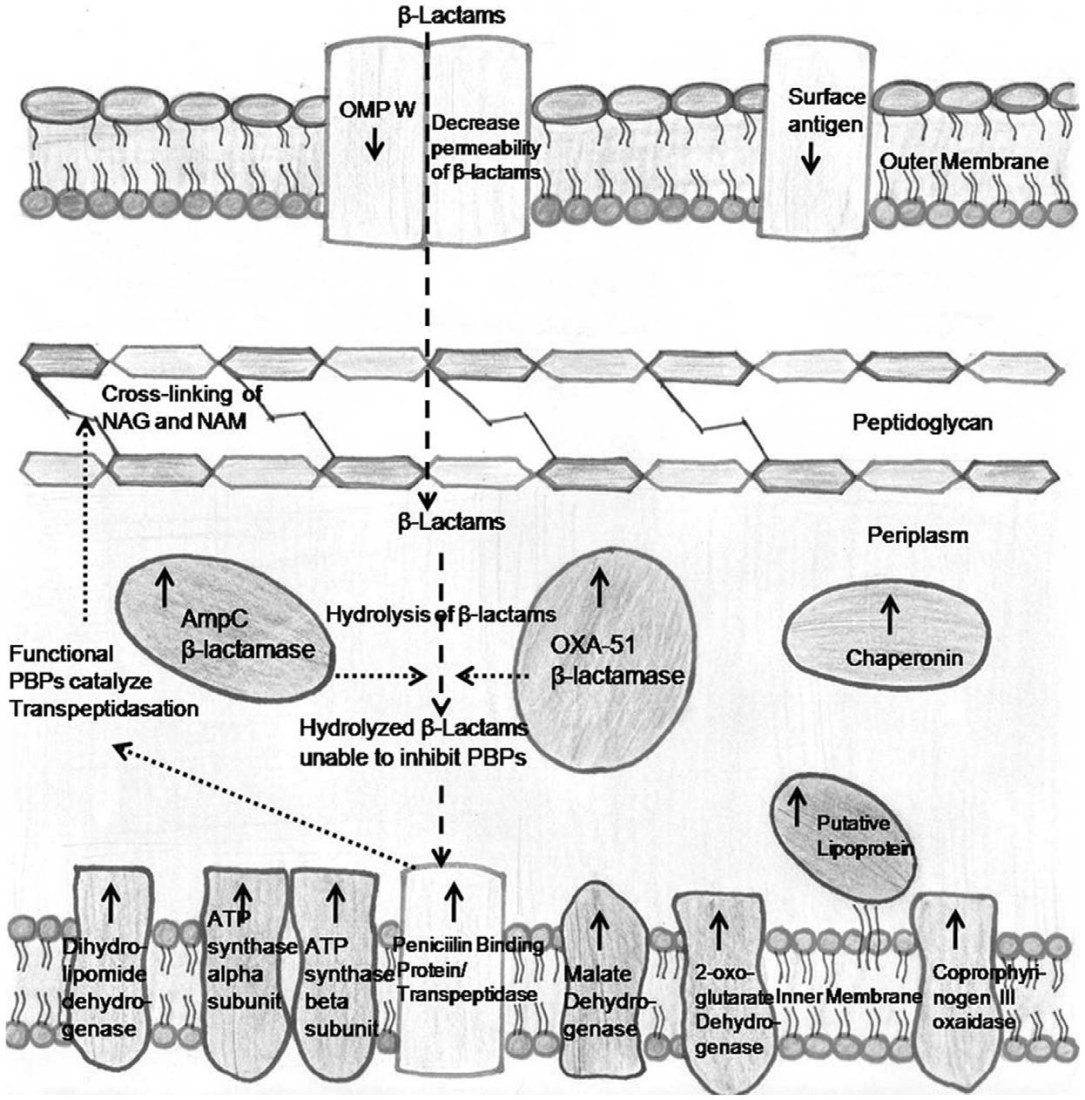
Schematic presentation of upregulated (↑) and downregulated (↓) proteins in the carbapenem-resistant *A.* *baumannii*.

## Results and Discussion

ATCC and 25 clinical strains of *Acinetobacter* were identified as *A.* *baumannii* based on a routine set of biochemical tests [30], [22]. MIC of imipenem for all the twenty five clinical strains and a standard reference strain, ATCC 19606 were determined by Agar dilution method. Details of these resistant strains and their MICs for imipenem were as follows: ATCC 19606 (MIC; 0.1 µg/ml), 14 strains with MIC; 0.2 to 8 µg/ml, RS030603, RS8238, RS259, RS117, RS322, RS8031, RS278, RS144, RS256, RS119, RS336, RS355, RS325 and RS3320, 8 strains with MIC; >8 to 64 µg/ml: RS170, RS71, RS122, RS250, RS398, RS688, RS15368, RS1556 and 3 strains with MIC; >64 µg/ml: RS168, RS721, RS307. MIC data revealed that 9 of 25 of clinical isolates of *A.* *baumannii* were highly resistant (MIC ≥64 µg/ml) to imipenem. It was alarming to see a steep increase to 36% in resistance of *A.* *baumannii* as compared to the incidence of about 20% found five years ago in our hospital [6]. This clearly showed the rise of resistance of *A.* *baumannii* to carbapenems in A.I.I.M.S. Clinical strains with MIC >64 µg/ml pose a bigger threat (than the rest of the strains) as they are becoming increasingly resistant. RS307 (MIC, 128 µg/ml) is a representative of the three clinical strains with high MIC. The rationale for selecting RS307, is to compare ‘naturally induced’ resistance in RS307 with that of ATCC19606. Comparative proteomics has been used as an approach to understand the resistance mechanism in the microorganisms where the ATCC strain was considered as a reference (susceptible) strain [31], [32], [17], [11]. We have also performed proteomics of clinical resistant strains with lower MICs: RS122 with MIC, 32 µg/ml and RS259 with MIC, 1 µg/ml. However, results showed only two differentially expressed proteins in each strain (ATP synthase, DNA protection during starvation protein in RS122 (Table S1) and Elongation factor Tu, 3-isopropyl malate dehydratase in RS259 (Table S2) respectively).

### Differential In-Gel Electrophoresis (DIGE) and Mass Spectrometry

In the present study, DIGE revealed the presence of about 1200 protein spots in each individual profile of native and RS307 strain (Figure S1). After DIGE experiments, the data was analysed using two independent software programs, DeCyder v7.0 (Figure S2) and Progenesis same spot v3.2 (Figure S3). The rationale behind using two different programs for the analysis of DIGE data is to validate the differentially expressed proteins with more authenticity and confidence [28]. However, the difference in the approach of the two softwares is an added advantage in identifying the proteins with better accuracy without which some proteins would have been missed or incorrectly detected.

One would expect that the quantitative changes estimated between a set of two cross matched protein spots would be the same or at least similar in terms of fold change by the two software packages. In fact, the two softwares indeed similarly identified differentially expressed protein as “up/down” however, there is a significant discrepancy in the fold change that is estimated [28]. Results tabulated in table 1 clearly show that protein fold changes were substantially different in both packages, for example, DeCyder and Progenesis has given a fold change in production of protein S.no:1 as 7.23 and 2.6 respectively. This issue was very elegantly discussed in the recent technical notes [28], which clearly states that results obtained for protein fold changes and p-value were substantially different in Progenesis and DeCyder, which indicates that in spite of using internal standards, quantification is software dependent [28]. A glance at Table 1 also tells us that the maximum fold change detected by DeCyder is much higher ratio (upto 7.23 and −6.85) as compared to Progenesis Same Spots (3.7 and −7.2). The fold change of particular spot calculated by the software depends on the way spot boundaries are defined [28]. Spot detection is therefore dependent on segmentation of a spot, for example splitting the same into two or more spots. For example spot 425 was detected as single spot by Progenesis while the same is detected as multiple spots by DeCyder which often results into oversplits.

Interestingly, our results reveal that the same proteins were identified as upregulated proteins and downregulated proteins by DeCyder and Progenesis, however, only differ in the estimation of fold change and p-value (see table 1, upregulated 14 spots and down regulated 4 spots). Although protein spots 15–19 (table 1) were not detected by Progenesis but were identified by DeCyder with high significance (p-value and fold change) and therefore can not be ignored.

Relative production of selected proteins from resistant strain RS 307 and ATCC 19606 strain is presented in Table 1 and Figure 1. For the convenience of clearly distinguishing all the protein spots in figures and throughout the text, we have designated the proteins and referred them by their master number from DeCyder. The identified proteins (Figure S4) were further classified according to their production levels in RS307 as compared to ATCC; a) upregulated b) down regulated. Most of the differentially expressed proteins in the carbapenem resistant strain (present study) are also present in sensitive isolate ATCC as it is known from the genome database (NCBI) of *Acinetobacter baumannii.* The upregulated proteins and their relevance to the resistance are discussed below.

### Upregulated Proteins

The following discussion deals with those proteins whose levels are relatively high in resistance phenotype RS307. On the basis of their cellular functions, all the upregulated proteins in RS307 are associated with: (i) β-lactamase (ii) metabolism and energy production (iii) protein synthesis machinery and iv) chaperonin.

#### β-Lactamases

It is well known that β-Lactamases hydrolyze β-lactam ring and neutralize the effect of antibiotics like imipenem, penicillin and meropenem etc. β-Lactamases are produced by Gram negative bacteria as a means of self-defence against β-lactam antimicrobials; and *A.* *baumannii* is no exception.

##### Class C (AmpC) β-lactamas

Spot no. 603 (43.2 kDa, 9.49 pI) was upregulated by 5.34 fold and identified as class C (AmpC) β-lactamase of *A.* *baumannii* (Table 2). Based on the recombinant experiments by over expressing AmpC β-lactamase in *K. pneumoniae*, Martinez-Martinez *et al.,* found that MIC of carbapenems indeed increased significantly from 1 to 64 µg/ml in the recombinant organism harbouring AmpC β-lactamase gene and loss of porins [33]. Further, a report from France also demonstrates the emergence of peculiar AmpCs (ADCs) in the hospital isolates of *A.* *baumannii* and their role in broad spectrum cephalosporin resistance [34]. This elevated level of AmpC may be due to the presence of the insertion sequence ISAba1 upstream to AmpC gene [35]. These reports are in full agreement with the present finding of elevated levels of AmpC in the RS 307 of *A.* *baumannii* which directly correlates with increased resistance to carbapenem.

##### OXA-71/OXA-51, a class D β-lactamase

The other β-lactamase which has been found to be upregulated in RS 307 was the protein spot no.745 which has Mol. Wt. 30.6 kDa and a pI of 8.48. Mass spectrometry identified it as OXA-71, a class D β-lactamase of *A.* *baumannii* (Table 2). OXA-71 has 99% similarity with OXA-51 of *A.* *baumannii* [36]. The over-production of the OXA-51 is an interesting observation from the present study. Presence of different variants of OXA was reported in imipenem resistant *A.* *baumannii* in different parts of the world- India [36], Argentina [37], Taiwan [7] and Brazil [38]. A hospital study from Taiwan has shown that high load of OXA-51 could predict the mortality of patients suffering from *A.* *baumannii* bacteremia [7]. Similarly, Costa *et. al.* reported that presence of OXA-23 in *A.* *baumannii* confers resistance to most β-lactams including imipenem, aztreonam and Ceftazidime [38]. Figueiredo et al. reported that overexpression of OXA β-lactamase gene is mediated by the presence of insertion sequence IS*Aba* in OXA gene [39], [40]. Therefore, it is explicit that OXAs carrying *A.* *baumannii* are increasingly becoming endemic.

#### Proteins associated with metabolism and energy production

Increased metabolic activity and energy production are required for pathogen to resist high antibiotics load/stress [20], [41]. Metabolic enzymes are reported to be present in the inner membrane fraction [17], [20], [24], [42], [43]. The metabolic changes may be a consequence of the biological cost of antibiotic resistance [44]. In the present study, seven protein spots are overproduced which found to have a role in metabolism and energy production.

##### ATP synthase

DeCyder analysis showed that spot 455 (50.3 kDa and 5.0 pI) was upregulated by 4.95 fold and been identified as beta subunit of ATP synthase which is crucial for ATP synthesis (Table 2). This is supported by the findings of Lee et al., where the authors have noticed increased production of beta-subunit of ATP synthase when *A.* *baumannii* cultures were grown containing imipenem (32 µg/ml) [30]. It may be mentioned here that besides ATP synthase, Lee et al., also found some more induced proteins in *A.* *baumannii*, which will be discussed in sequel. Identification of upregulated proteins, spot no. 436 and 434 as ATP synthase subunit alpha (55.5 kDa, 5.29 pI) (Table 2) suggests their important role in maintaining functional conformation of F_1_ subunit of ATP synthase. Based on 2D-DIGE, Santos et al., has shown that piperacilln-induced resistance strain of *E. coli* showed elevated levels of ATP synthase [45]. All these suggest the activation of energy efficient mechanism by the bacteria as a means of survival.

##### Dihydrolipoamide dehydrogenase

Spot no. 428 (42.5 kDa and 5.64 pI) was found to be upregulated by 3.01 fold and was identified as dihydrolipoamide dehydrogenase of *A.* *baumannii* (Table 2). Dihydrolipoamide dehydrogenase provides acetyl-CoA required for TCA cycle and biosynthesis of fatty acids. High levels of dihydrolipoamide dehydrogenase was also reported in several other microorganisms like antimicrobial peptide-resistant *Vibrio parahaemolyticus* [46], toluene-resistant *Pseudomonas putida* [47] and tellurite- resistant *E. coli* [48]; suggesting the need of the enzyme for addition energy requirement in survival.

##### Malate dehydrogenase

Protein spot no. 645 was upregulated by 6.5 fold and identified as malate dehydrogenase of *A.* *baumannii* with Mol. Wt. of 35.4 kDa and pI of 5.2 (Table 2). Malate dehydrogenase being a part of TCA cycle (which acts as anapleurotic cycle) is important for energy production. Increased levels of malate dehydrogenase is an attempt to overcome the oxidative stress as seen in *E. coli* [49], [48], during the adaptation from anaerobic to aerobic conditions and also in the biofilm formation in *A.* *baumannii* [21].

##### Enzymes involved in porphyrin synthesis

Two protein spots 432 and 650 were identified as 2-oxoglutarate dehydrogenase complex and coproporphyrinogen III oxidase of *Acinetobacter* respectively (Table 2) and are known to have a role in porphyrin synthesis. However, it has been suggested that increased production of prophyrin helps in oxidative phosphorylation in *E. coli* [50].

#### Protein synthesis machinery

It has been seen that under stress conditions like antibiotics, the microorganism activate the protein synthesis so as to take care of the increased metabolic requirements. Supporting this phenomenon in RS 307 of *A.* *baumannii*, we report the upregulation of 30 S, 50 S ribosomal proteins and elongation factor Tu (EF-Tu) which are part of the protein synthesis machinery (Table 2). Recently, these proteins are reported in membrane fraction of *A.* *baumannii* [17], [20] and other negative bacteria [51]. Because of their critical role in resistance, they have been chosen as the target for a number of antibiotics against gram negative bacteria [52]. The increased production of ribosomal proteins in the clinical strain may be one of the ways by which organism tries to counterbalance the effect of antibiotics. Upregulation of EF-Tu is the consequent event of upregulated 30 S and 50 S ribosome and promotes translation. Yun et al., also report elevated ribosomal proteins in the carbapenem resistant strain of *A.* *baumannii* [20] High copy number of mRNA is another factor which increases the protein synthesis. Elevated levels (fold change 3.5) of the alpha subunit of DNA-directed RNA polymerase, (spot 595) suggests the overall activation of transcription and thereby protein synthesis under the antibiotic stress in *A.* *baumannii*.

#### Chaperonin required to maintain native conformation of proteins

As explained above, overall protein synthesis related proteins were more abundant in resistant bacteria and it is also known the protein after synthesis is brought into its native, active conformation by the group of proteins, “chaperonins”. Chaperorins are usually found in the cytosol but there are reports which have shown the presence of chaperonins in the inner membrane fraction [17], [53]. This suggests that there is an increased need of chaperonins in resistance [54], in order to maintain the newly translated protein in the correctly folded form. This is exactly what is observed in RS 307 of *A.* *baumannii*, which increased the production of two, 60 kDa chaperonin (spot no 365) and TCP-1/cpn60 chaperonin family protein (spot no. 366) of *A.* *baumannii* (Table 3) under carbapenem stress. It suggests the requirement of efficient folding machinery in the carbapenem-resistant strain. Lee *et al.,* found that cpn60 chaperonin was elevated in the hetero-resistance *A.* *baumannii* under imipenem induction [30].

#### Putative lipoprotein

Transporters as the name suggests, supply of nutrients/ions/metabolites and required for survival of the microorganism. We found high concentration (4.9 fold) of one such transporter of lipid, putative lipoprotein (spot no. 1012) with mol. wt. of 16.4 kDa (Table 3). In Gram-negative bacteria, mature lipoproteins are localized to various sites within the cell wall. They are targeted to the periplasmic face of the inner or outer membranes by the lipoprotein localization machinery [55]. Hence it is possible for lipoproteins to be present in the inner membrane fraction at the time of isolation from *A.* *baumannii*. Thus lipoprotein could be used to generate novel countermeasures to infections caused by *A.* *baumannii.*

#### Unknown proteins

Further, two other protein spots, 902 and 904 (26.4 kDa, 4.79 pI) which were identified as the carbapenem-associated resistance protein precursor of *Acinetobacter baumannii* (Table 3). Function of this precursor is unknown in *A.* *baumannii.*

### Downregulated Proteins

In addition, *A.* *baumannii* also changes the protein production of several other proteins so as to evade the host defence mechanism.

#### Surface antigens

Two such protein spots no. 1266 and 1269 were found to be diminished by 6.37 and 4.79 respectively and have been identified as surface antigen of *A.* *baumannii* (Table 3). Surface antigen on the membrane of gram negative bacteria is recognised by host defence mechanism. Cell surface antigens are involved in cellular recognition processes by antibodies produced by host against bacteria hence either down regulation or modification of surface antigen is advantageous for pathogenic bacteria [56].

#### Ribosome-recycling factor

As the name suggests ribosome-recycling factor is involved in recycling of ribosome by splitting ribosome into 50 S and 30 S subunits. The lower levels of this protein increase translation of mRNA. However, in the present study, it was found that the resistant strain of *A.* *baumannii* suppressed (by 6.8 fold) the production of ribosome recycling factor.

#### OmpW

OmpW is an outer membrane protein and it was recently proposed that OmpW of *A.* *baumannii* is involved in the uptake of antibiotics like colistin and β-lactams (ceftriaxone) [16]. Although it is surprising to find OmpW in the inner membrane fraction, there are such reports in the past where OmpW is seen in periplasmic space [20]. Decreased production of OmpW (spot no 1004) by 6.23 fold in β-lactam- resistant *A.* *baumannii,* RS307 (Table 3), is in agreement with earlier reports in colistin-resistant *A.* *baumannii* and ceftriaxone-resistant strain of *Salmonella typhimurium* [57], [16]. Hence decreased OmpW perhaps reduces entry of β-lactams in the *A.* *baumannii* and makes β-lactams unavailable to its target (PBPs).

### Conclusions

Acquired resistance to carbapenem, in *A.* *baumannii* is a multifactorial phenomenon in which the bacterium applies a number of intricate and robustic means to evade the antimicrobial effect of antibiotic. Increased production of β-lactamases to cleave the antibiotic is the first step in developing resistance. It appears that *A.* *baumannii* employs efficient and robust metabolic mechanisms by increasing the production of metabolic enzymes, as is evident from the present data. It is tempting to speculate that, elevated levels of ribosomal proteins and chaperonin may help in rapid translation and proper folding of metabolic enzymes respectively. In addition, *A.* *baumannii* down regulates a number of proteins like surface antigen which assist in evading host defence mechanism. In conclusion, *A.* *baumannii* seems to apply a number of ways which work cooperatively and synergistically which help in developing resistance to antibiotic in clinical strain with respect to reference strain. Present finding are schematically summarized in Figure 2.

## Supporting Information

Figure S1 **Differential In-Gel Electrophoresis (DIGE) results of RS307 and ATCC19606.**
(PDF)

Figure S2 **DeCyder analysis of RS307 DIGE results.**
(PDF)

Figure S3 **Progenesis analysis of RS307 DIGE results.**
(PDF)

Figure S4 **Mascot search results for differentially expressed proteins in inner membrane fraction of RS307.**
(PDF)

Table S1 **Differentially expressed proteins identified in inner membrane fraction of RS122.**
(PDF)

Table S2 **Differentially expressed proteins identified in inner membrane fraction of RS259.**
(PDF)
