# Supplementary material for: Comparative Proteomics of Inner Membrane Fraction from Carbapenem-Resistant Acinetobacter baumannii with a Reference Strain
Source: PLoS One. 2012 Jun 26;7(6):e39451. doi: 10.1371/journal.pone.0039451 (PMC3383706; doi:10.1371/journal.pone.0039451)
Supplement: Figure S1 — Differential In-Gel Electrophoresis (DIGE) results of RS307 and ATCC19606. (PDF) [file pone.0039451.s001.pdf]

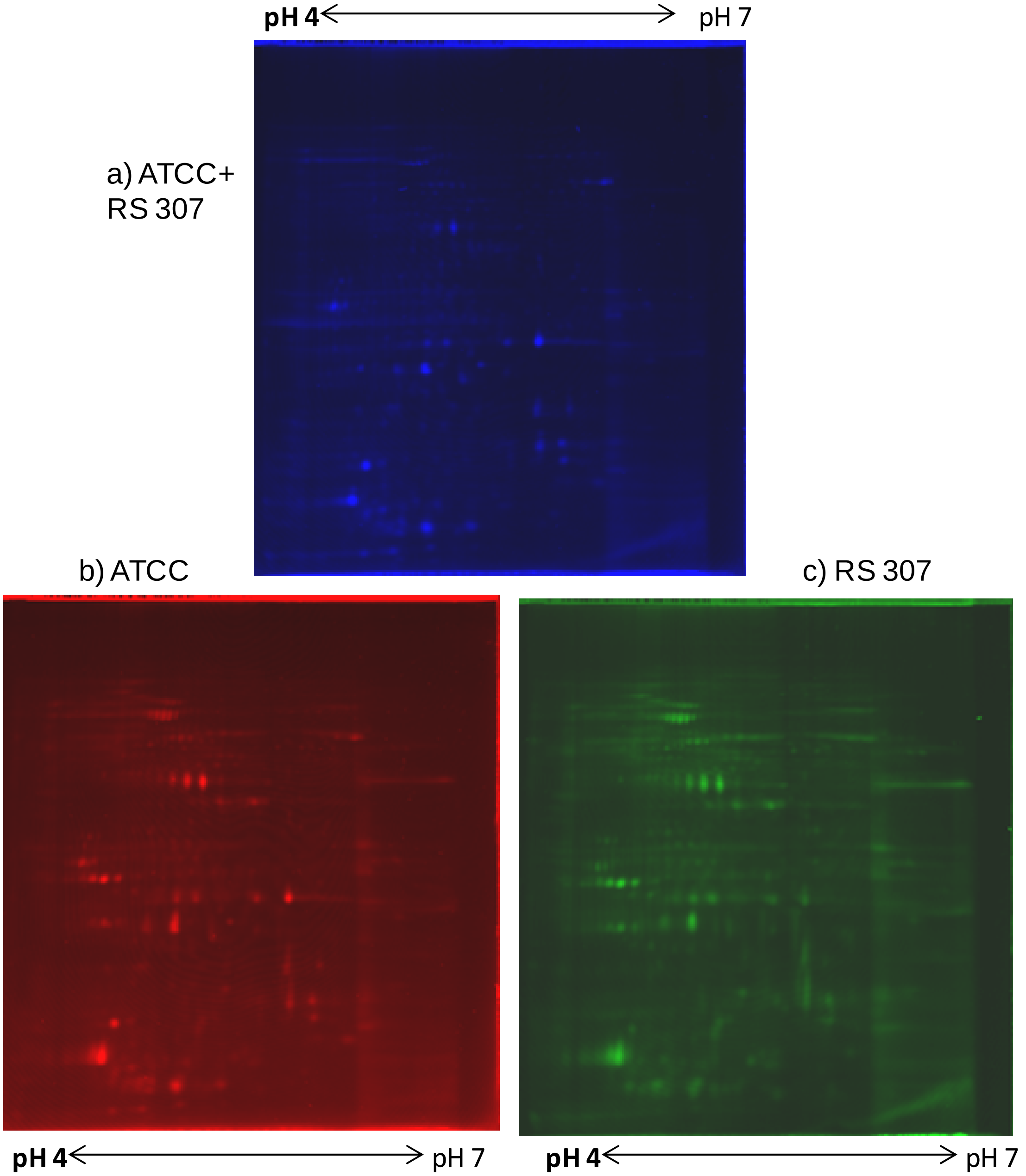

Figure S1a : DIGE of high resistant strain RS 307 and ATCC19606 of *A. baumannii*. Panel A represents Cy2 (200pmol, exci 448nm, emis. 520nm) labelled 50µg pooled equal fraction from ATCC and 307, Panel B represents Cy5 (200pmol, exci 630nm, emis 670nm) labelled 50µg protein from ATCC, Panel C represents Cy3 (200pmol, exci 532nm, emis 580nm) labelled 50µg proteins from high resistant strain 307. 150µg of total protein was loaded on 13 cm long 4-7L IPG strip and second dimensional electrophoresis was done using SE 600 ruby gel apparatus (16x18cm) on 12.5% SDS PAGE in dark and gel were scanned by typhoon variable mode imager for each Cy dyes.

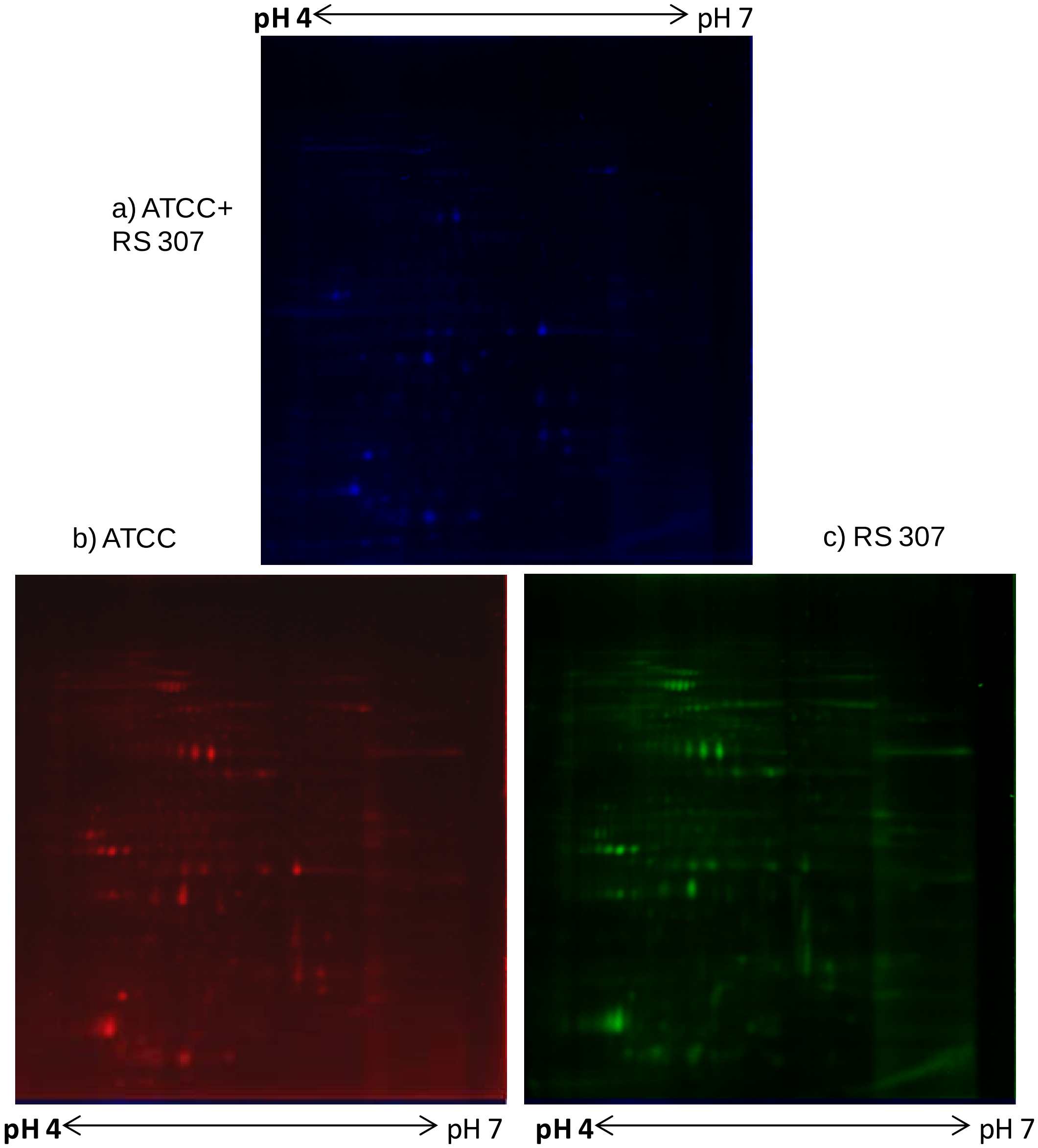

Figure S1b: DIGE of high resistant strain RS 307 and ATCC19606 of *A. baumannii.* All the experimental conditions are same as that in Figure S1a. However, the cultures are grown independently and the samples are biological replicate of experiment of RS 307 and ATCC.

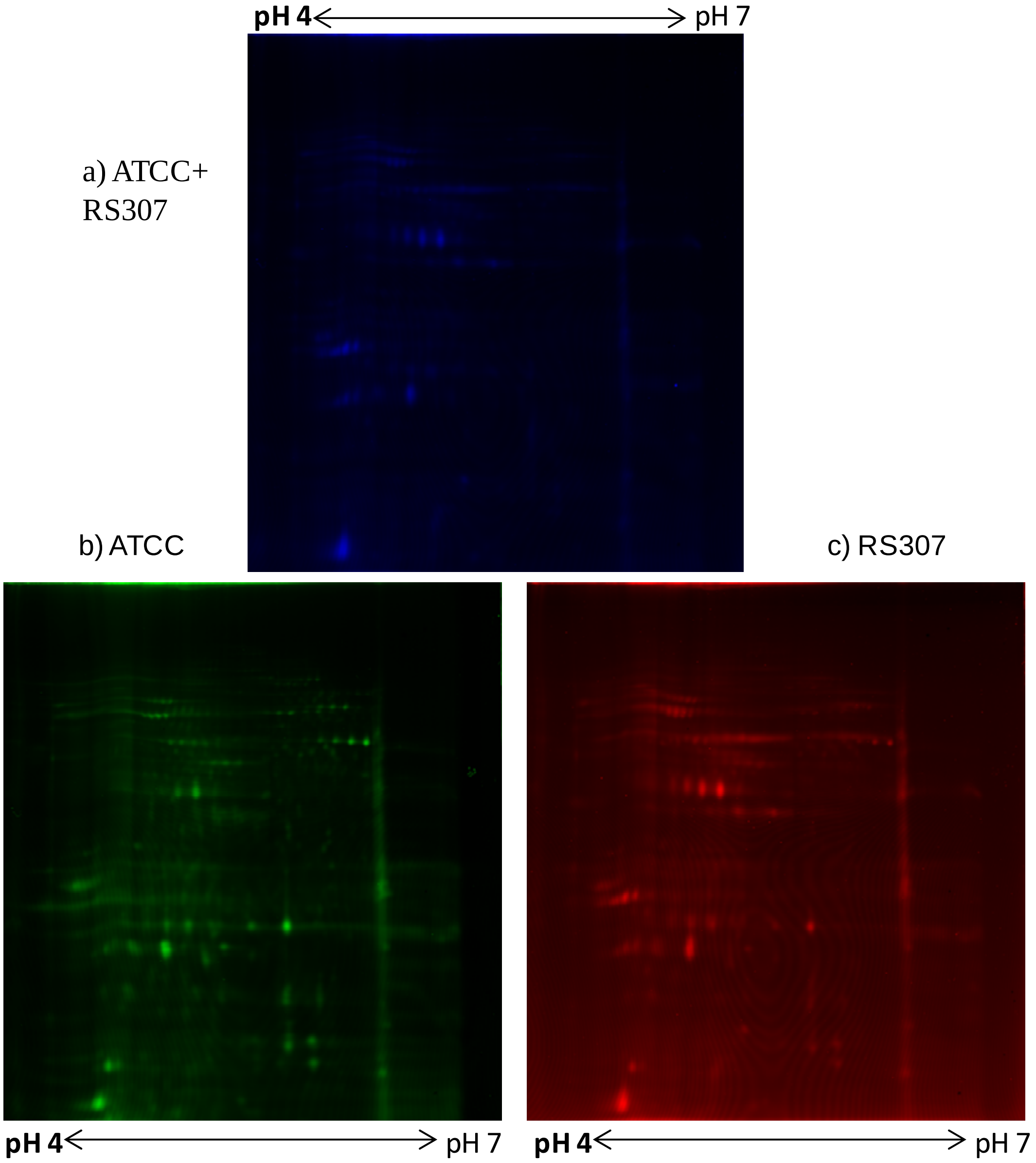

Figure S1c: DIGE of high resistant strain RS 307 and ATCC19606 of *A. baumannii*. All the experimental conditions are same as that in Figure S1a. However, the cultures are grown independently and the samples are biological replicate of experiment of RS 307 and ATCC. The dyes are swapped; the ATCC is labelled with Cy3 and RS307 with Cy5.
