# Supplementary material for: Comparative Proteomics of Inner Membrane Fraction from Carbapenem-Resistant Acinetobacter baumannii with a Reference Strain
Source: PLoS One. 2012 Jun 26;7(6):e39451. doi: 10.1371/journal.pone.0039451 (PMC3383706; doi:10.1371/journal.pone.0039451)
Supplement: Figure S2 — DeCyder analysis of RS307 DIGE results. (PDF) [file pone.0039451.s002.pdf]

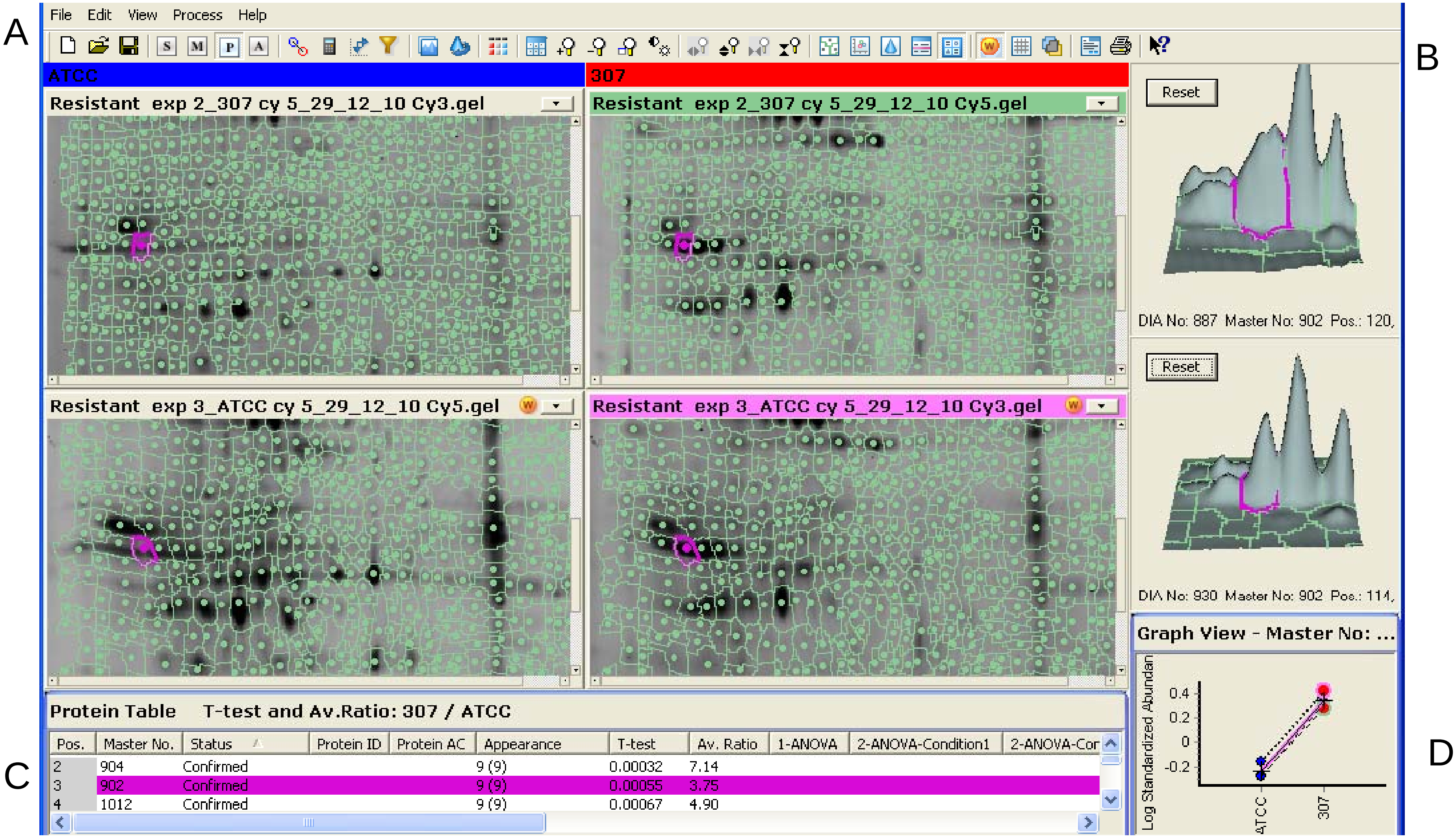

Figure S2.1: Representation of comparative DeCyder gel analysis of normalized gel image of native strain ATCC with resistant strain RS 307 using BVA module. All the combined results are displayed for upregulated master spot no. 902 of master gel. Panel A shows the spot 902 in both the compared strains, Panel B mean value of 3D expression of spot 902 in both the strains, Panel C represents graphical analysis for all three independent experiments, and Pane D shows statistical measurement of one way ANOVA and student‘t’ test.

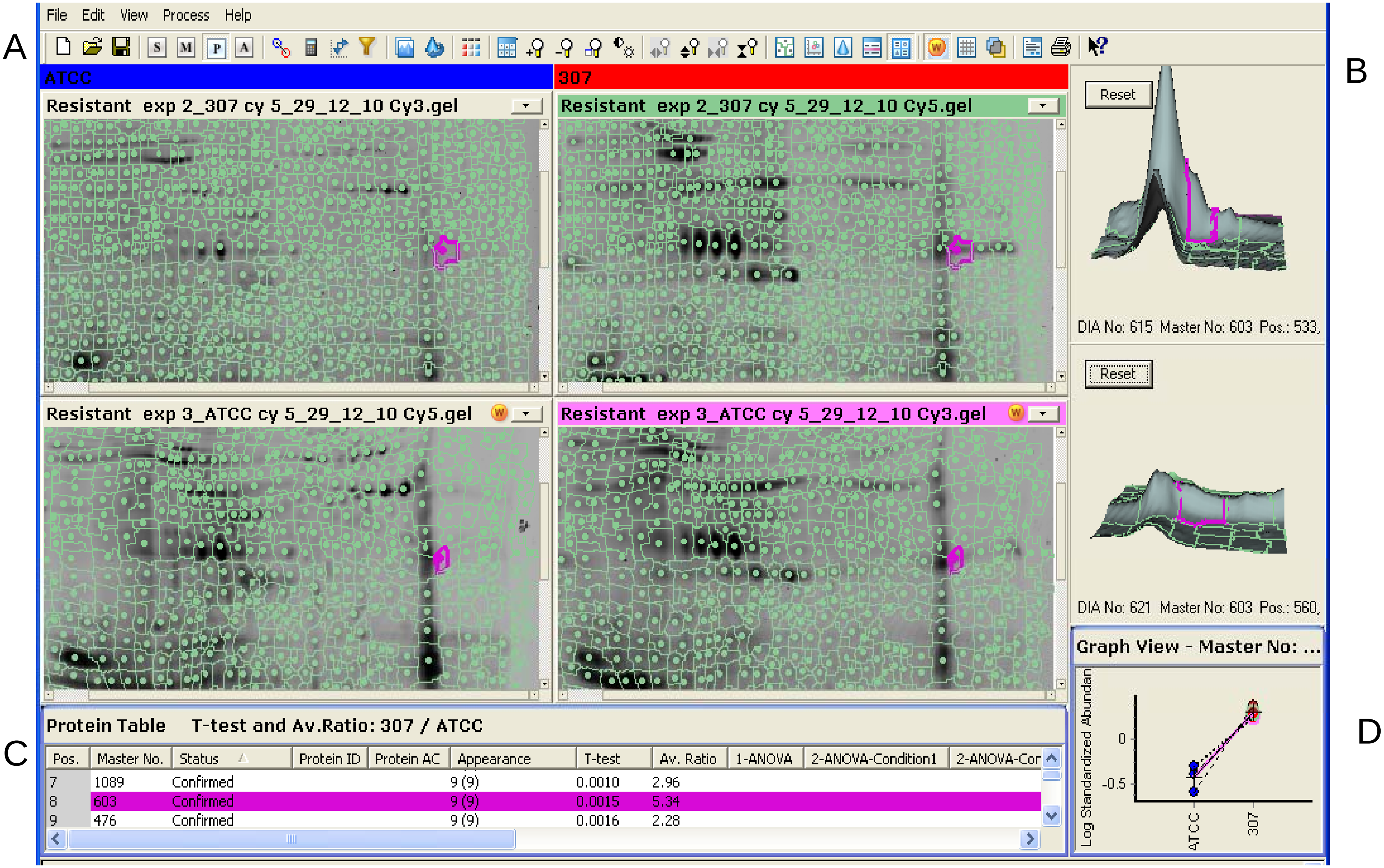

Figure S2.2: Representation of comparative DeCyder gel analysis of normalized gel image of native strain ATCC with resistant strain RS 307 using BVA module. All the combined results are displayed for upregulated master spot no. 603 of master gel.

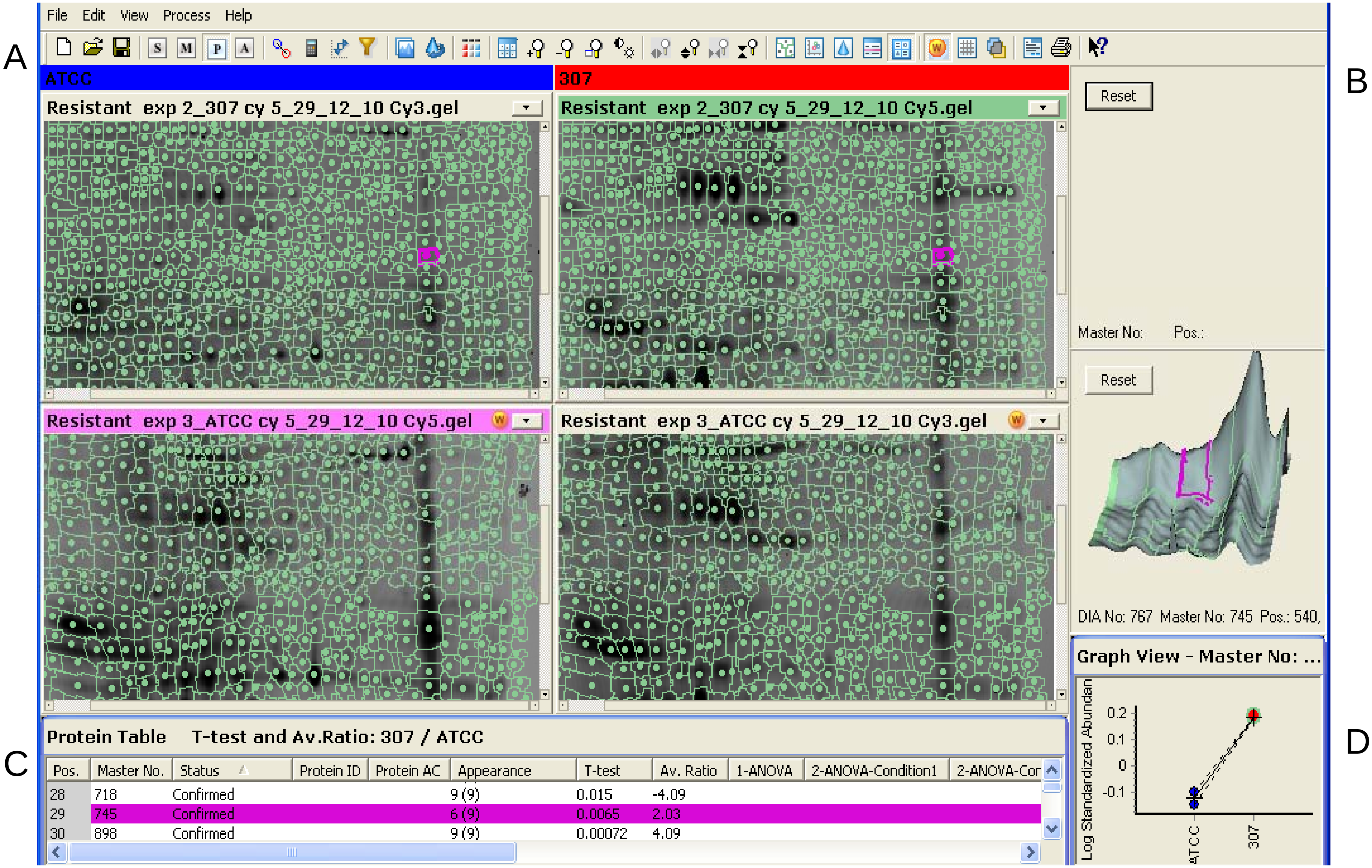

Figure S2.3: Representation of comparative DeCyder gel analysis of normalized gel image of native strain ATCC with resistant strain RS 307 using BVA module. All the combined results are displayed for upregulated master spot no. 745 of master gel.

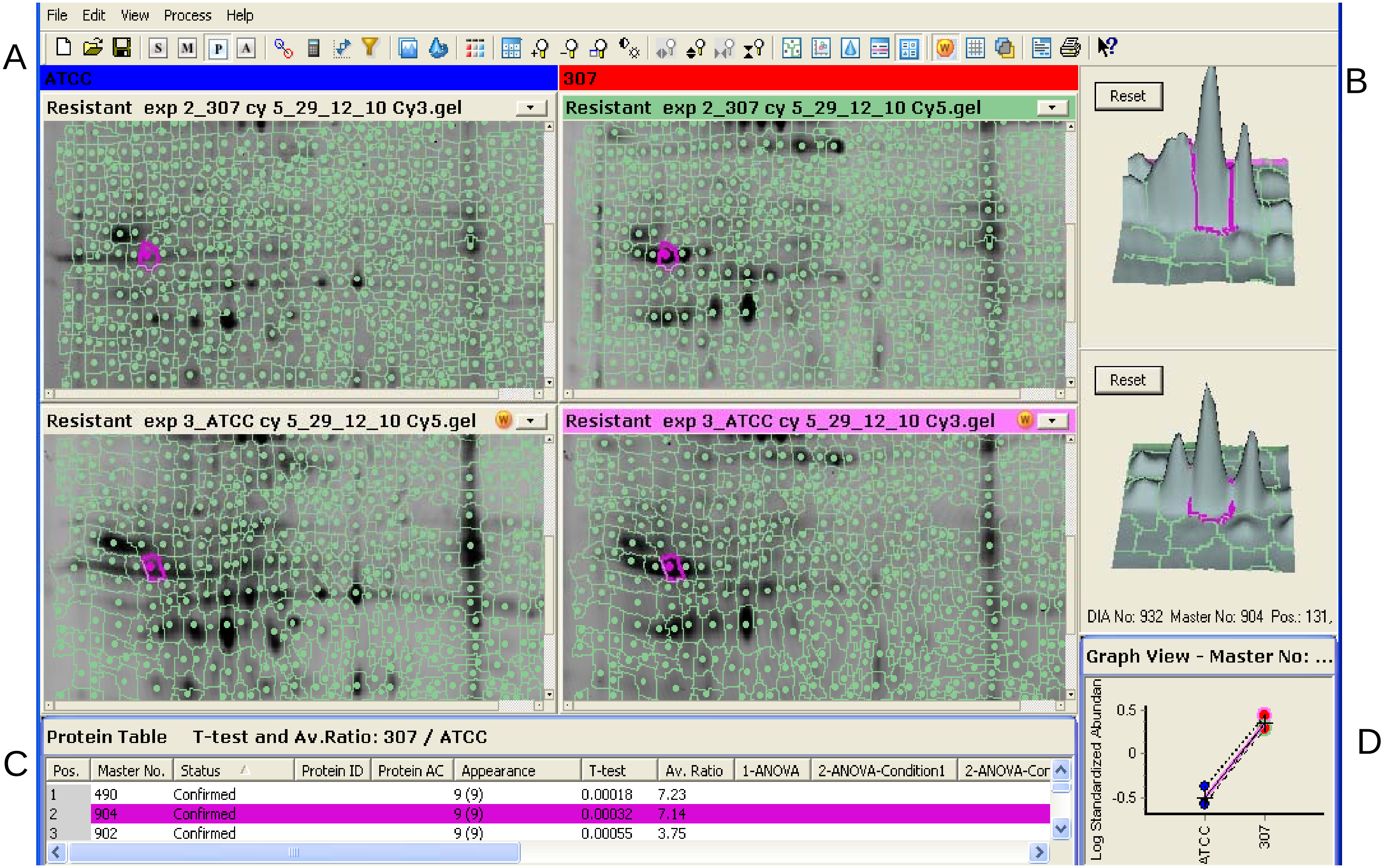

Figure S2.4: Representation of comparative DeCyder gel analysis of normalized gel image of native strain ATCC with resistant strain RS 307 using BVA module. All the combined results are displayed for upregulated master spot no. 904 of master gel.

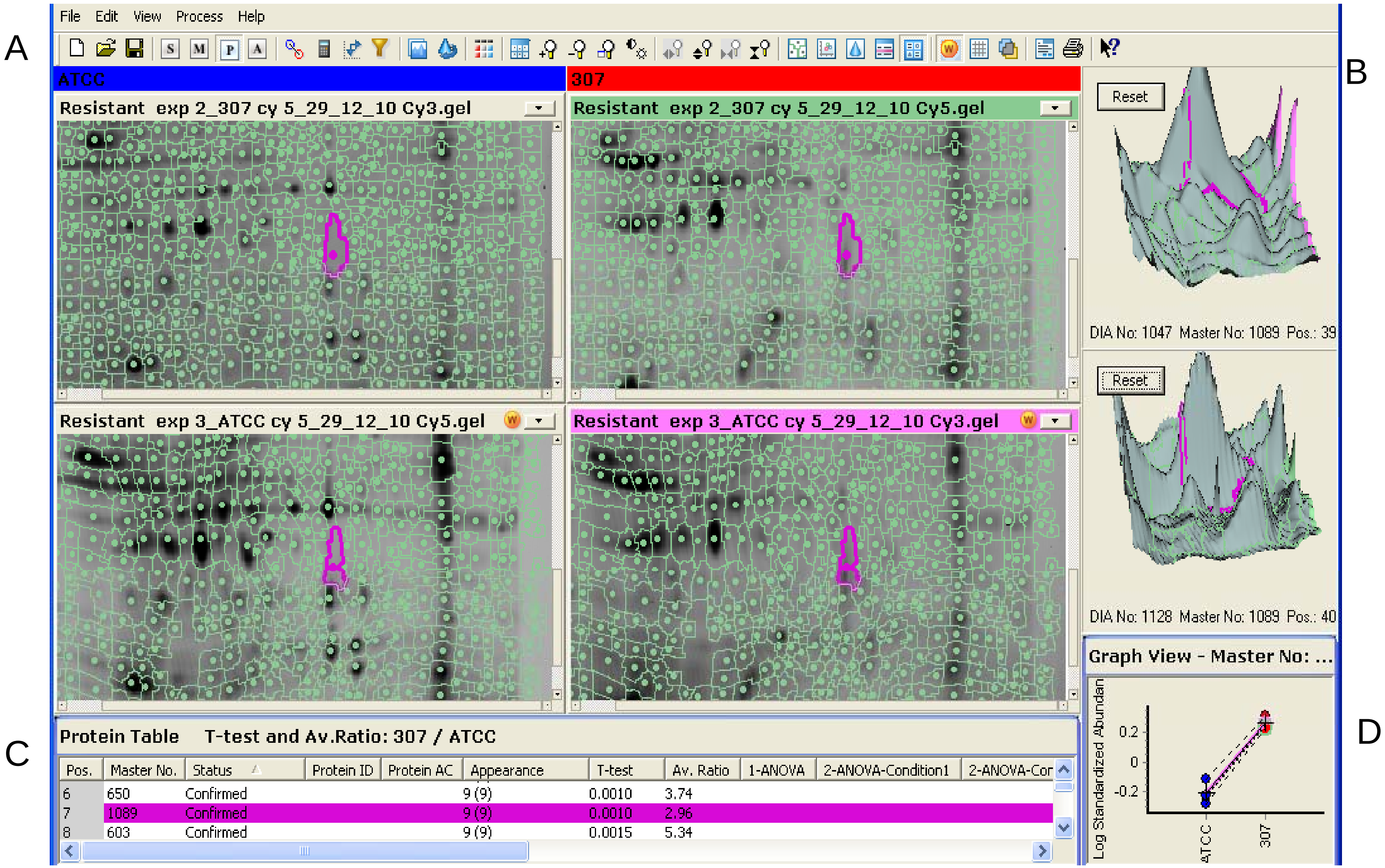

Figure S2.5: Representation of comparative DeCyder gel analysis of normalized gel image of native strain ATCC with resistant strain RS 307 using BVA module. All the combined results are displayed for upregulated master spot no. 1089 of master gel.

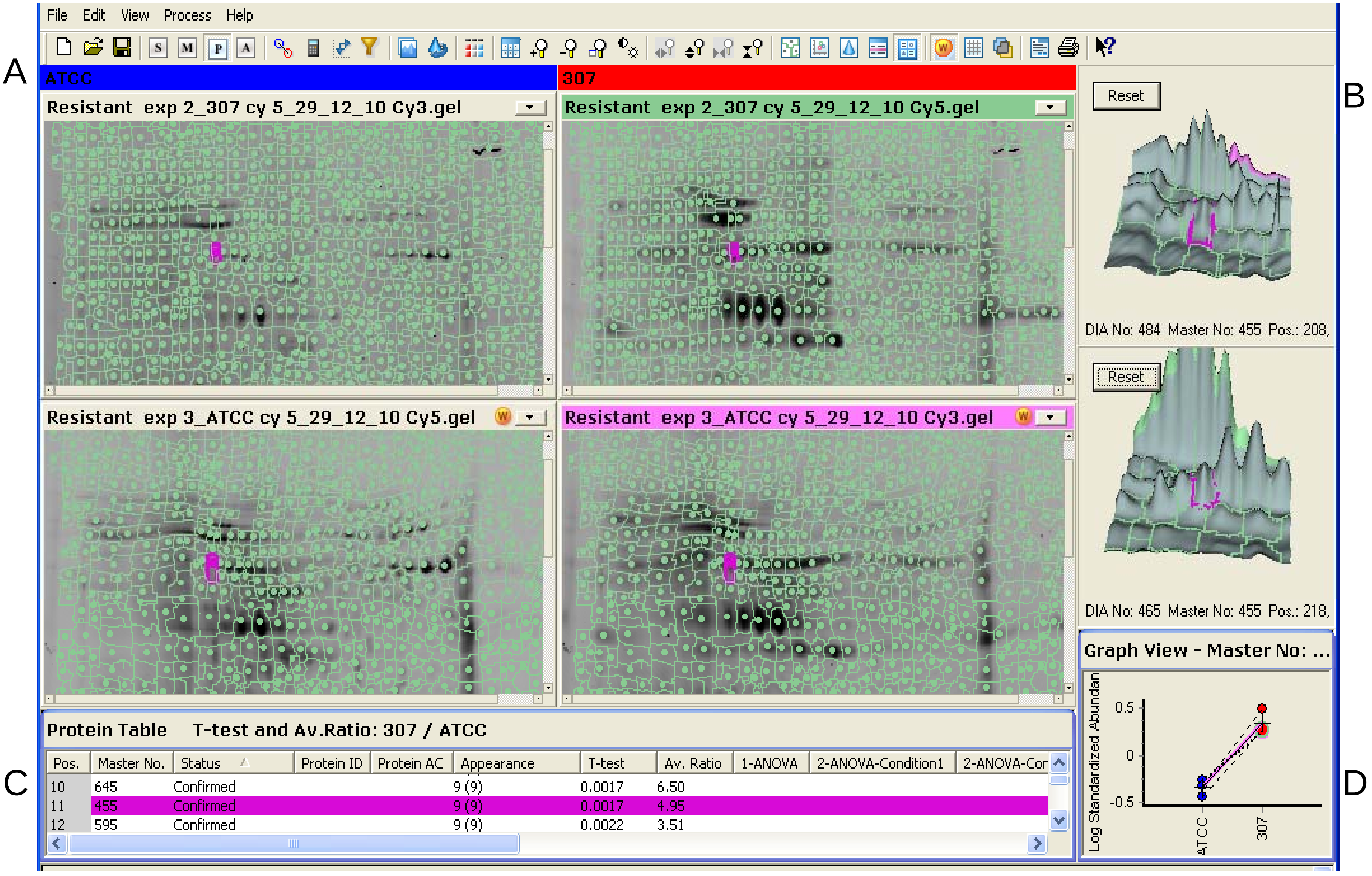

Figure S2.6: Representation of comparative DeCyder gel analysis of normalized gel image of native strain ATCC with resistant strain RS 307 using BVA module. All the combined results are displayed for upregulated master spot no. 455 of master gel.

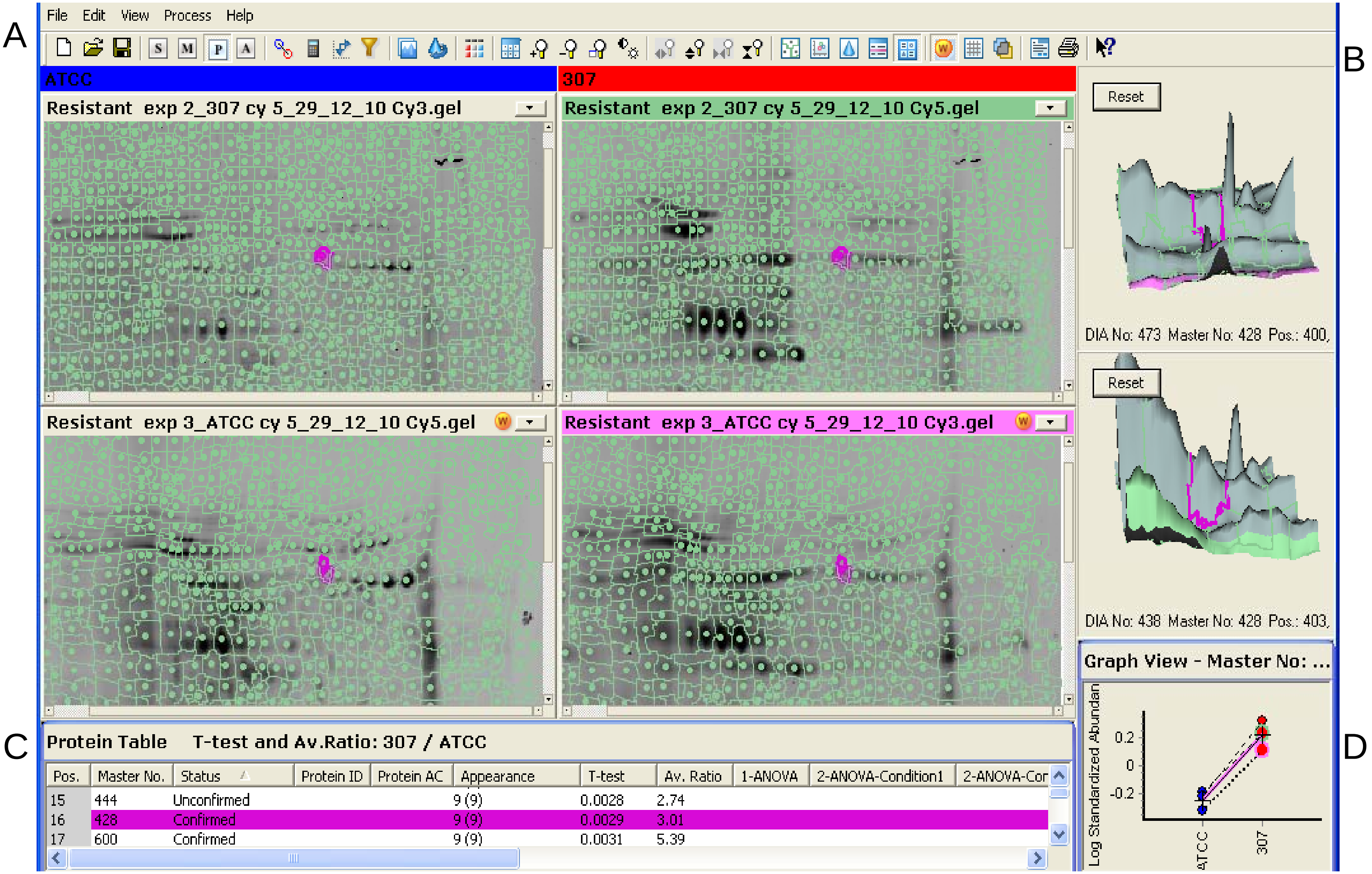

Figure S2.7: Representation of comparative DeCyder gel analysis of normalized gel image of native strain ATCC with resistant strain RS 307 using BVA module. All the combined results are displayed for upregulated master spot no. 428 of master gel.

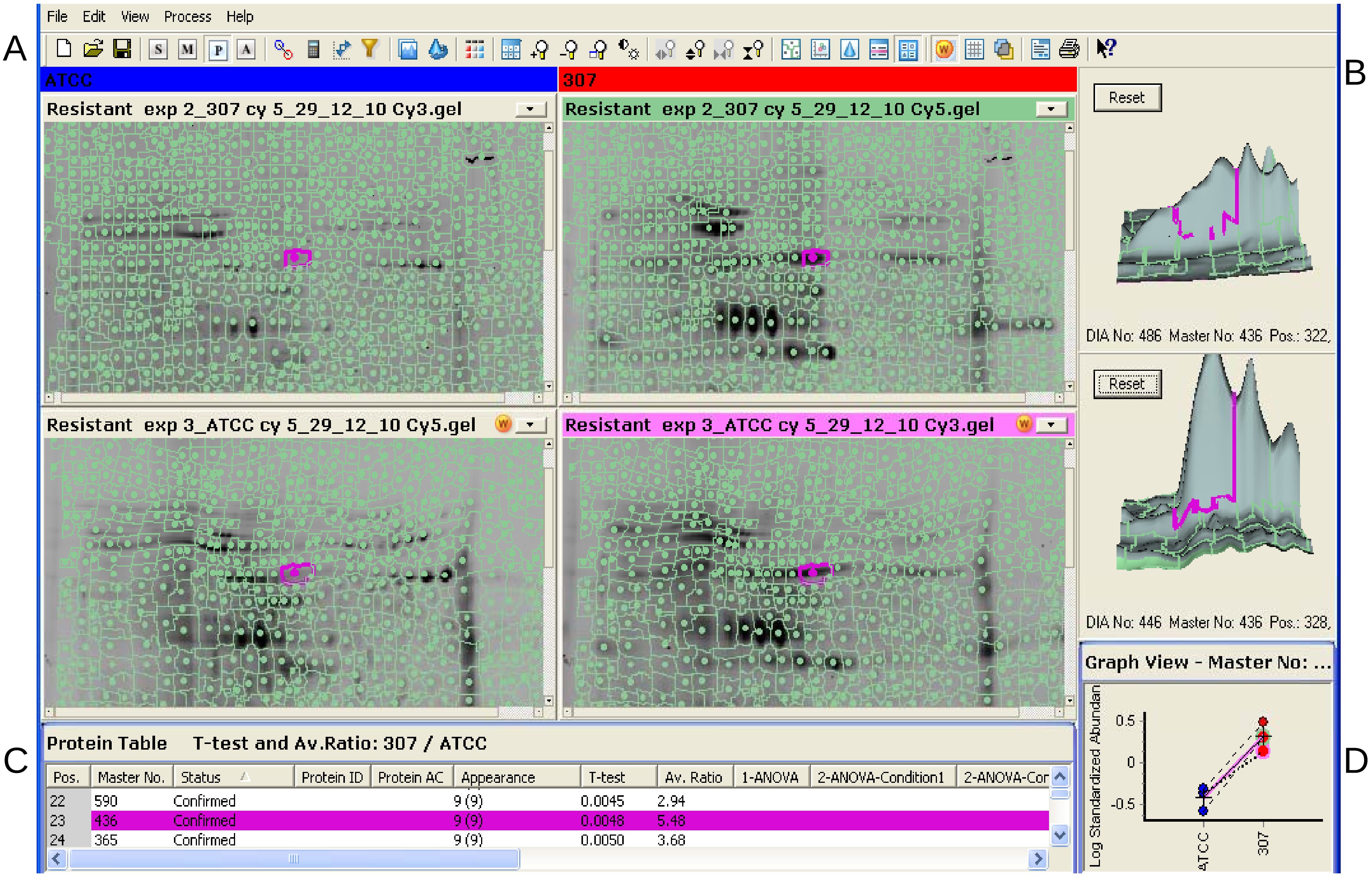

Figure S2.8: Representation of comparative DeCyder gel analysis of normalized gel image of native strain ATCC with resistant strain RS 307 using BVA module. All the combined results are displayed for upregulated master spot no. 436 of master gel.

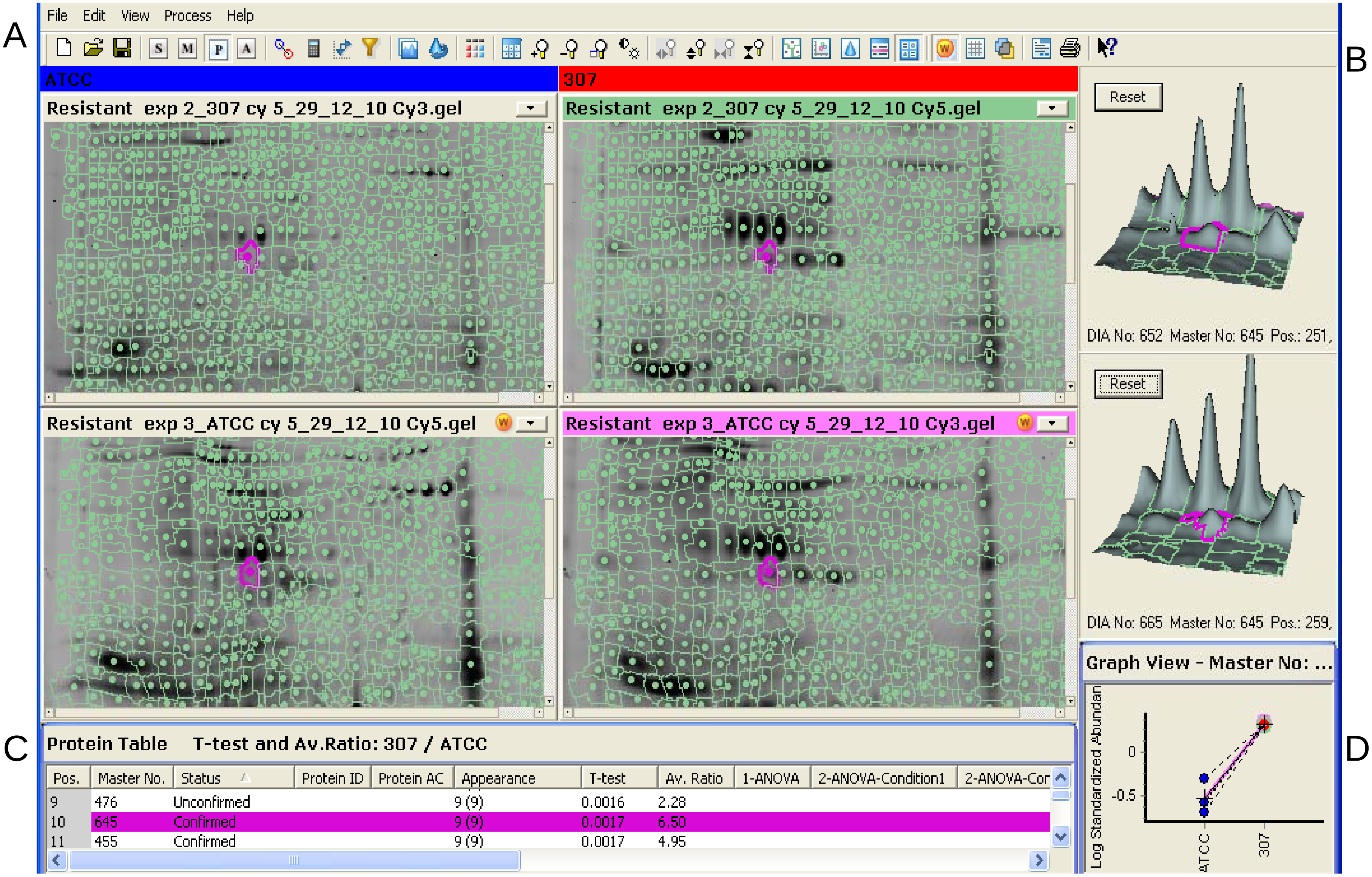

Figure S2.9: Representation of comparative DeCyder gel analysis of normalized gel image of native strain ATCC with resistant strain RS 307 using BVA module. All the combined results are displayed for upregulated master spot no. 645 of master gel.

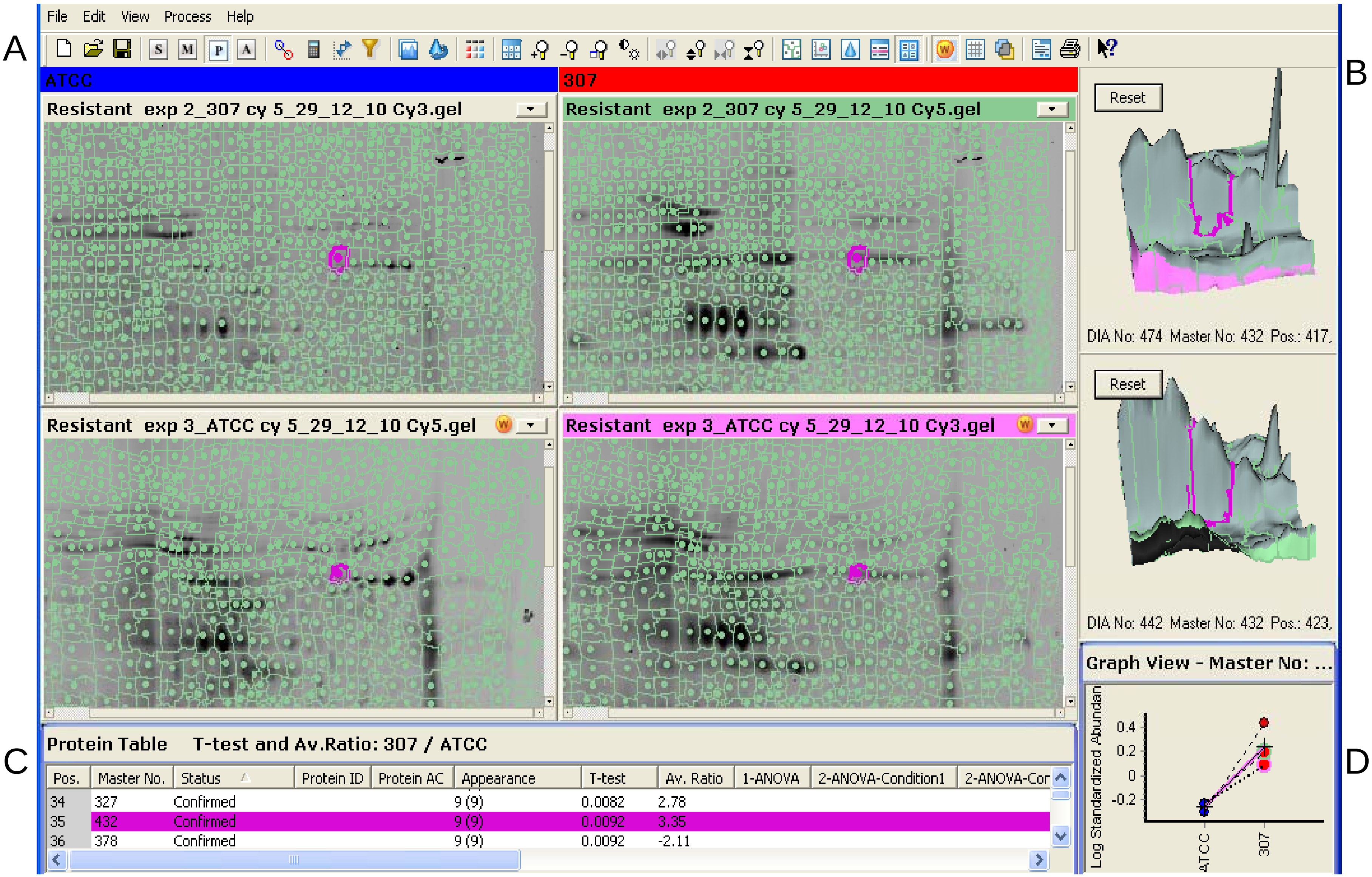

Figure S2.10: Representation of comparative DeCyder gel analysis of normalized gel image of native strain ATCC with resistant strain RS 307 using BVA module. All the combined results are displayed for upregulated master spot no. 432 of master gel.

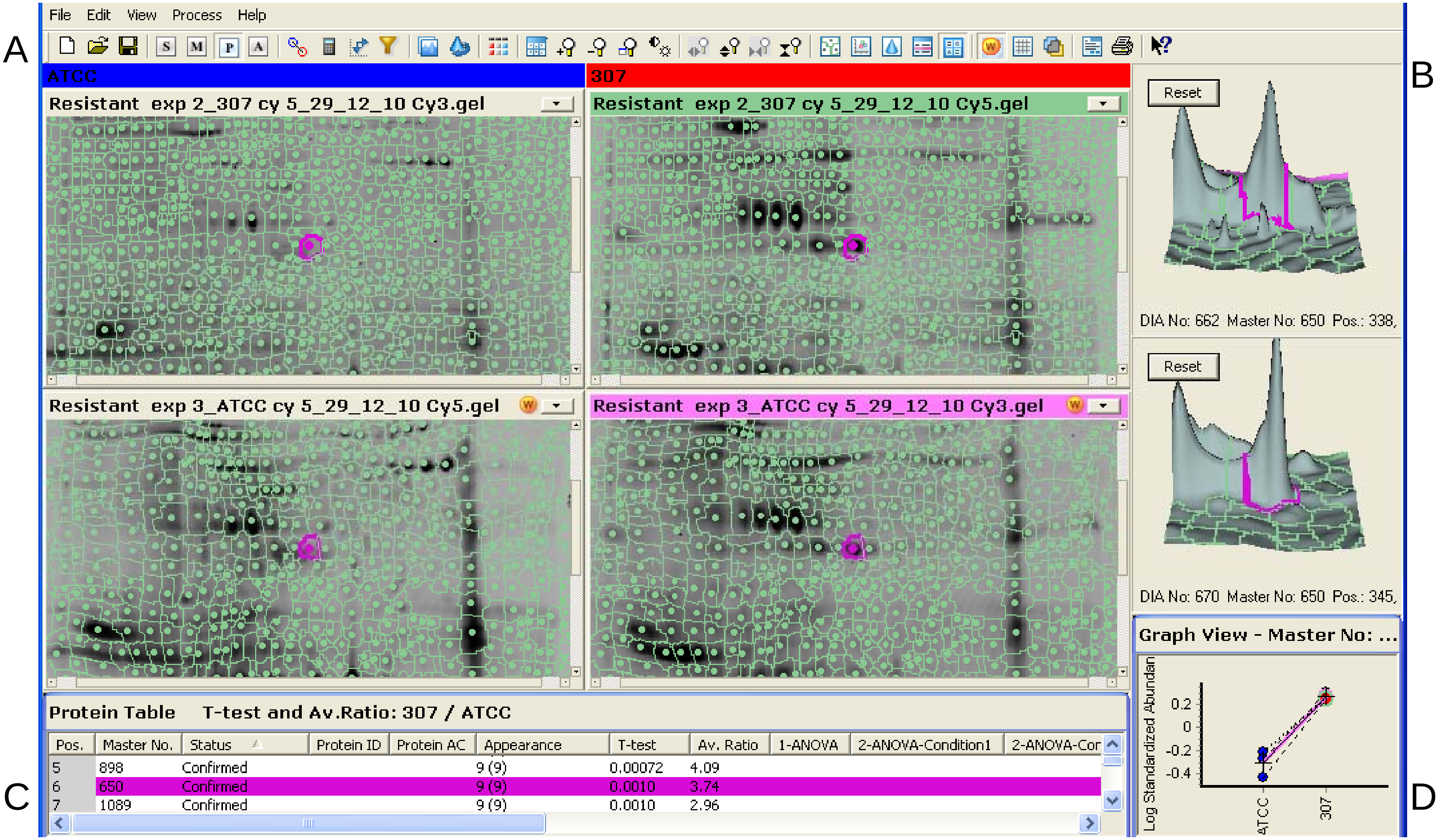

Figure S2.11: Representation of comparative DeCyder gel analysis of normalized gel image of native strain ATCC with resistant strain RS 307 using BVA module. All the combined results are displayed for upregulated master spot no. 650 of master gel.

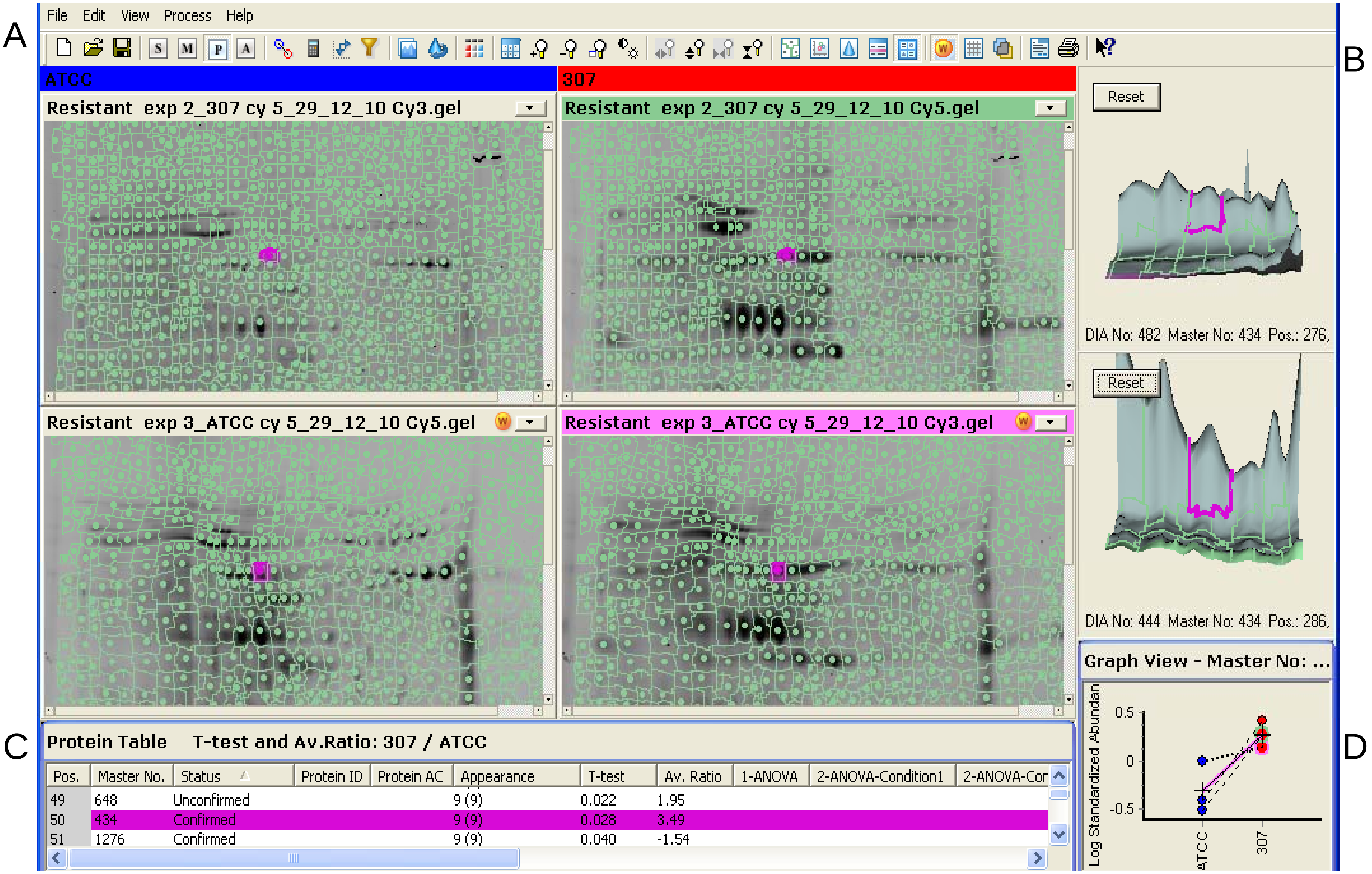

Figure S2.12: Representation of comparative DeCyder gel analysis of normalized gel image of native strain ATCC with resistant strain RS 307 using BVA module. All the combined results are displayed for upregulated master spot no. 434 of master gel.

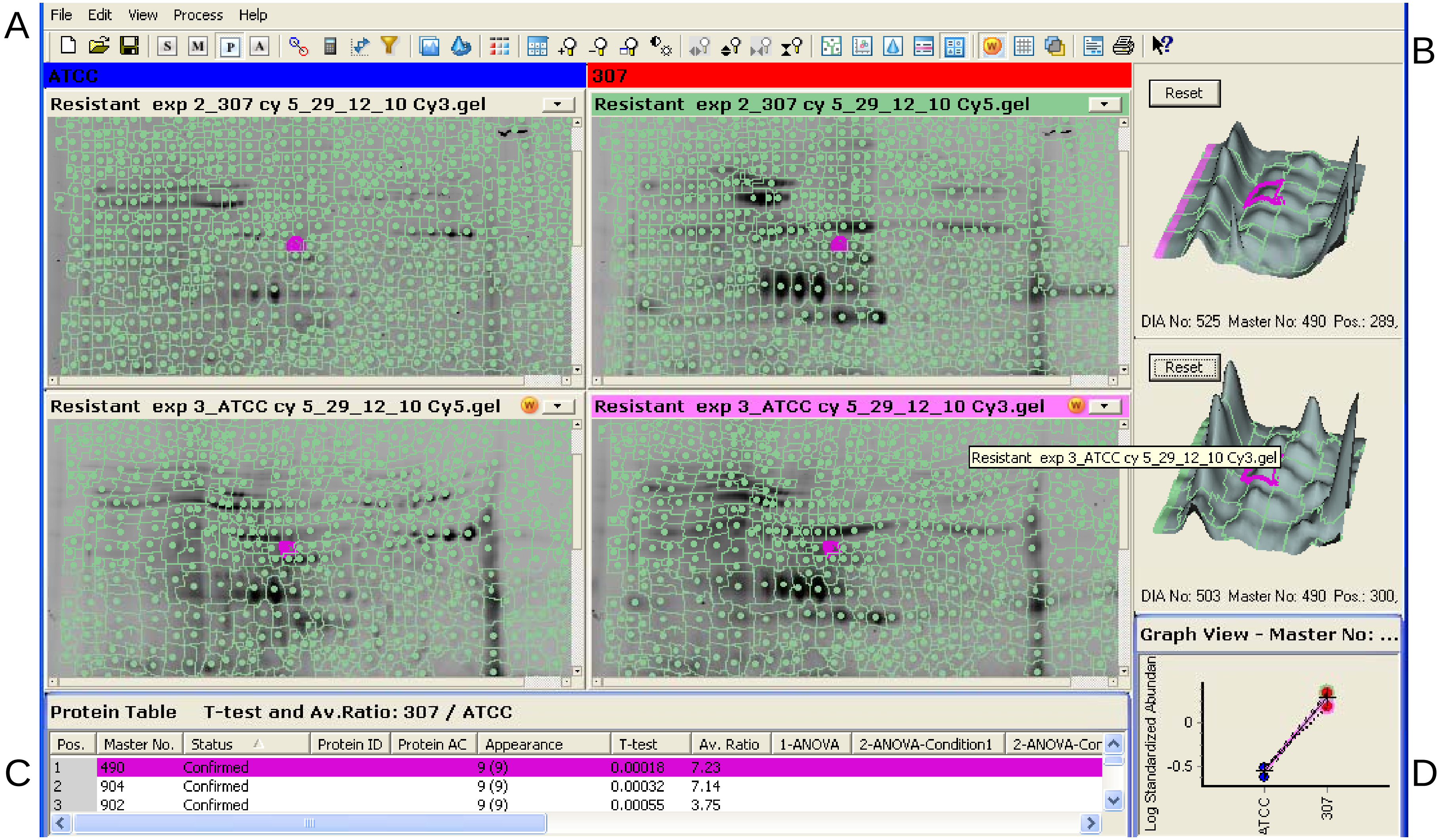

Figure S2.13: Representation of comparative DeCyder gel analysis of normalized gel image of native strain ATCC with resistant strain RS 307 using BVA module. All the combined results are displayed for upregulated master spot no. 490 of master gel.

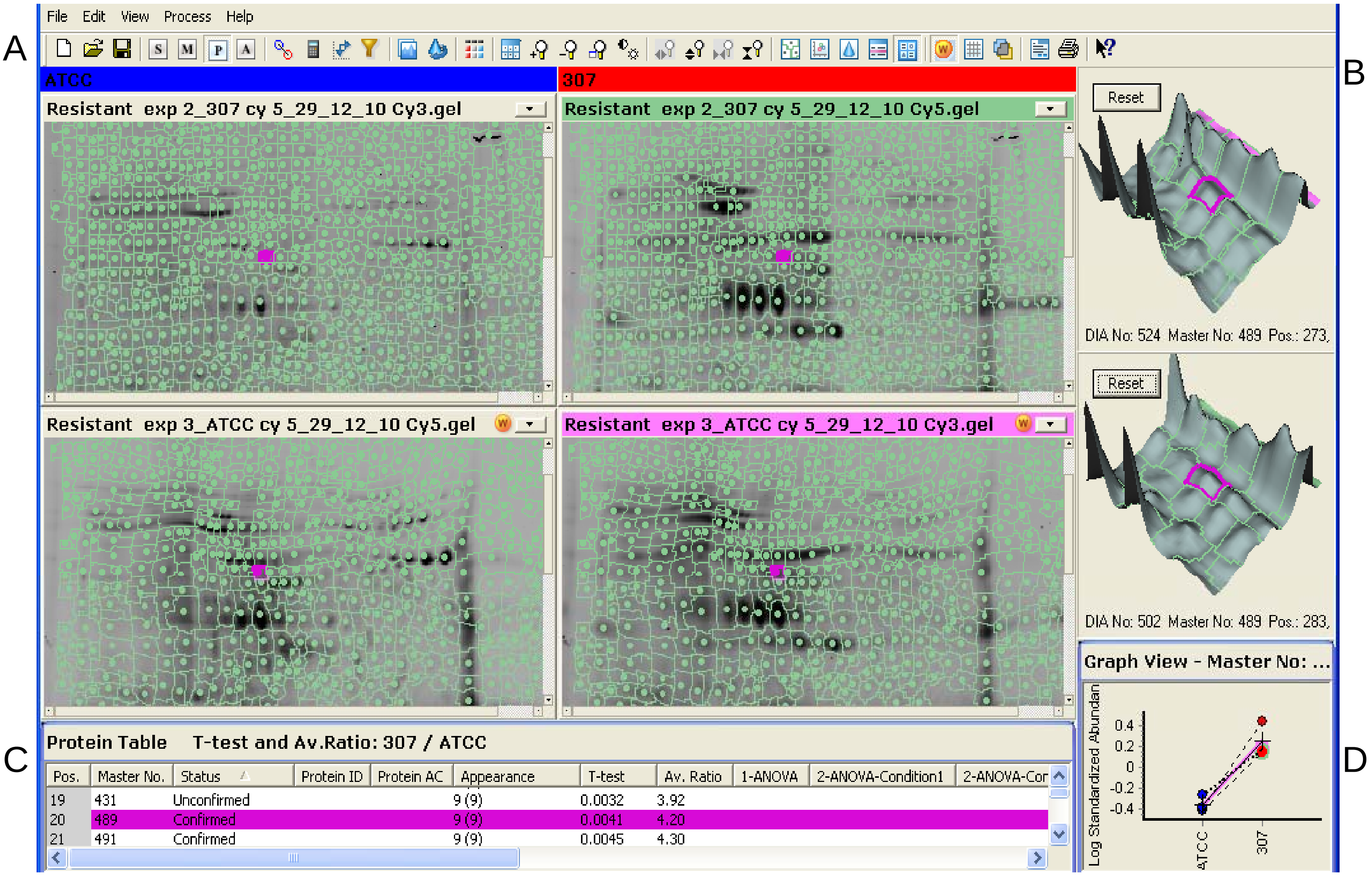

Figure S2.14: Representation of comparative DeCyder gel analysis of normalized gel image of native strain ATCC with resistant strain RS 307 using BVA module. All the combined results are displayed for upregulated master spot no. 489 of master gel.

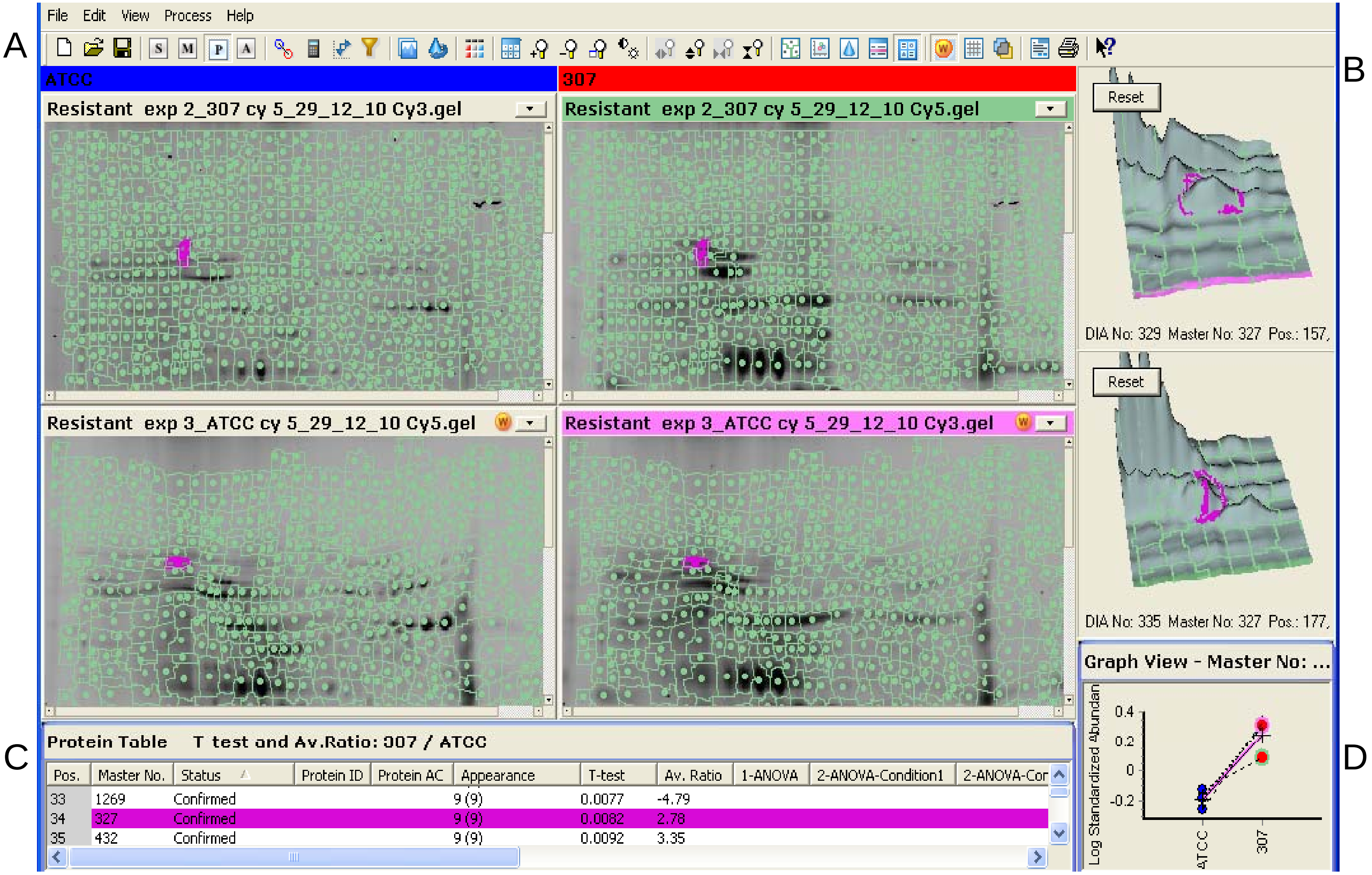

Figure S2.15: Representation of comparative DeCyder gel analysis of normalized gel image of native strain ATCC with resistant strain RS 307 using BVA module. All the combined results are displayed for upregulated master spot no. 327 of master gel.

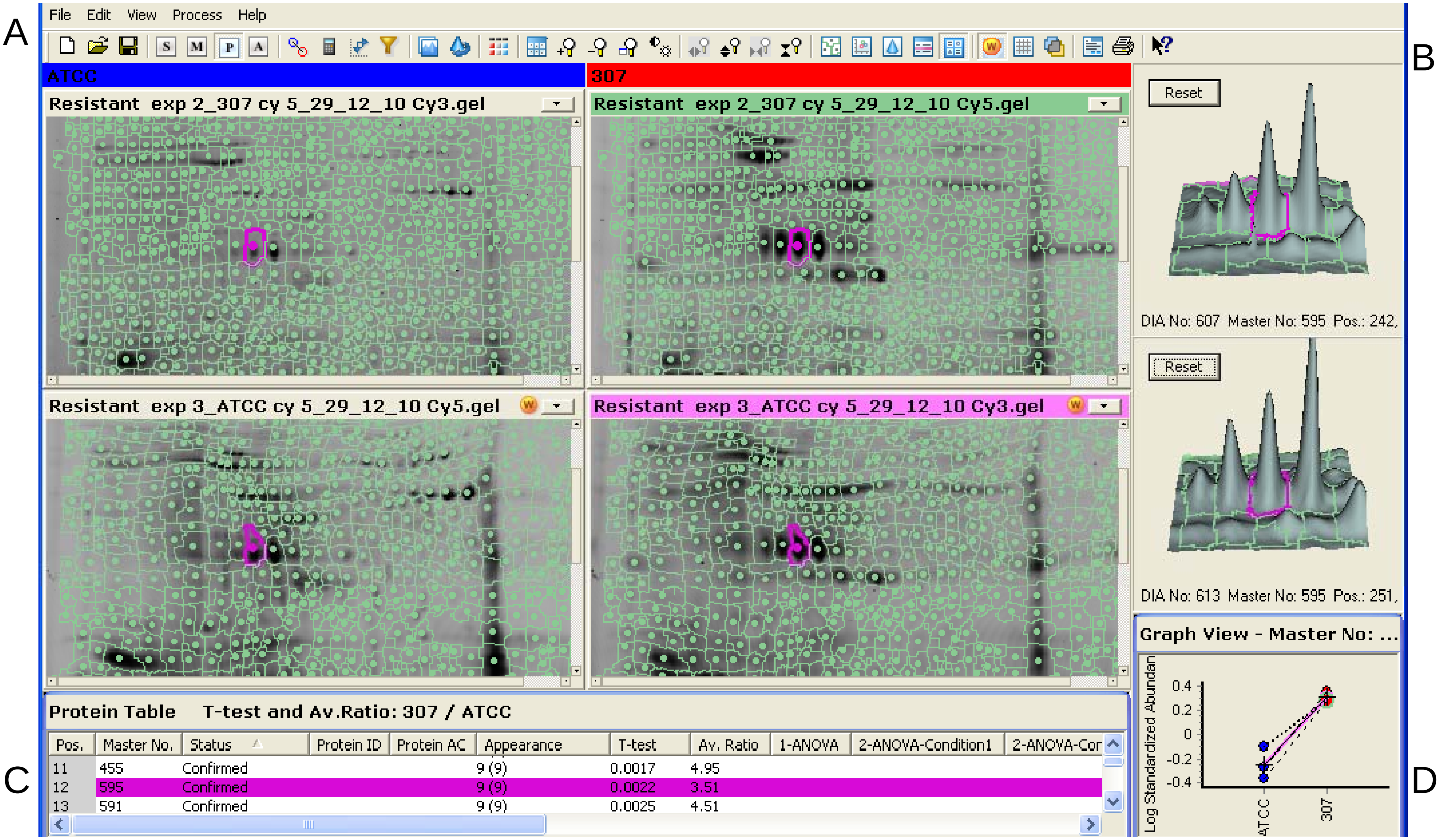

Figure S2.16: Representation of comparative DeCyder gel analysis of normalized gel image of native strain ATCC with resistant strain RS 307 using BVA module. All the combined results are displayed for upregulated master spot no. 595 of master gel.

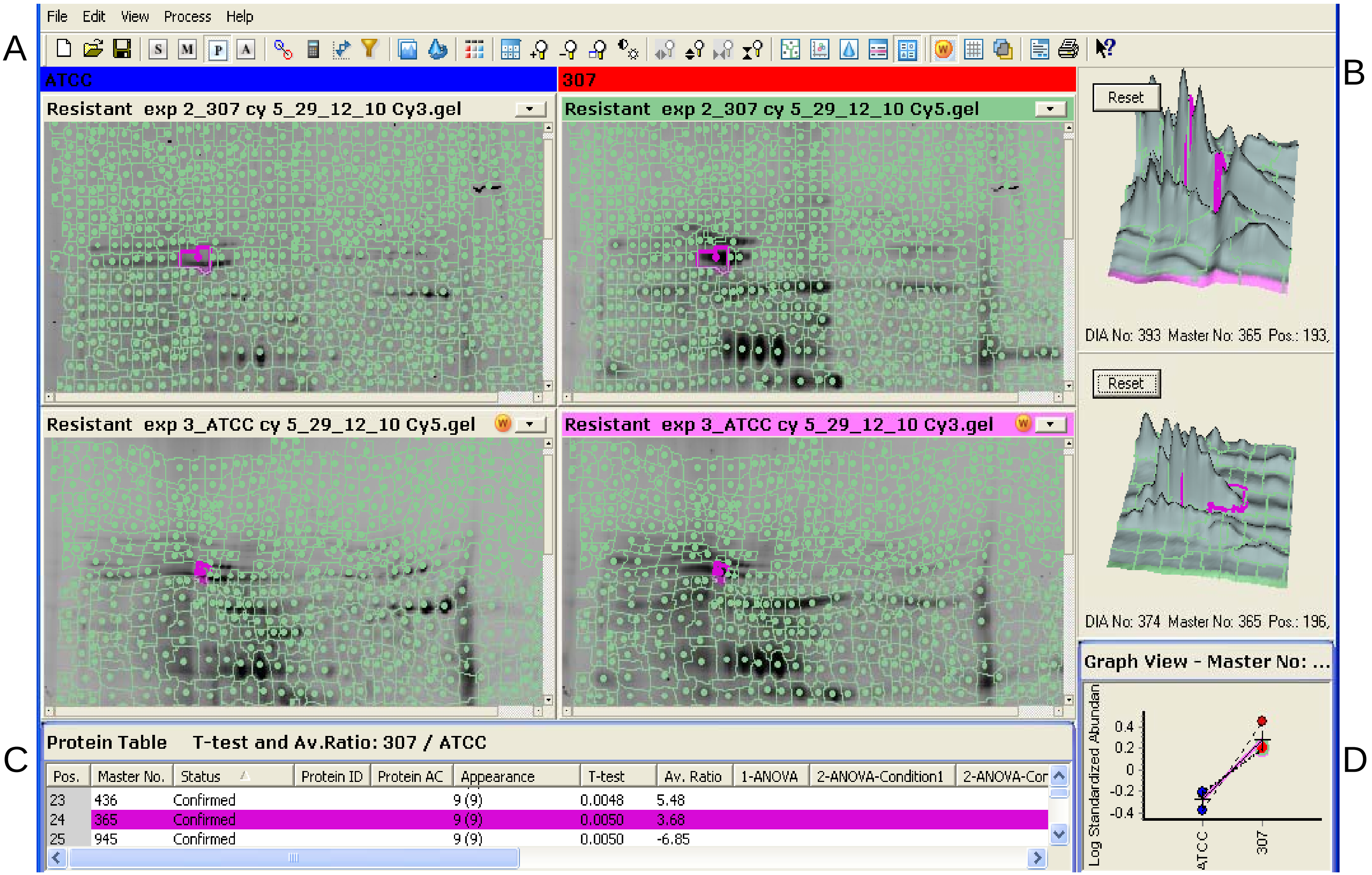

Figure S2.17: Representation of comparative DeCyder gel analysis of normalized gel image of native strain ATCC with resistant strain RS 307 using BVA module. All the combined results are displayed for upregulated master spot no. 365 of master gel.

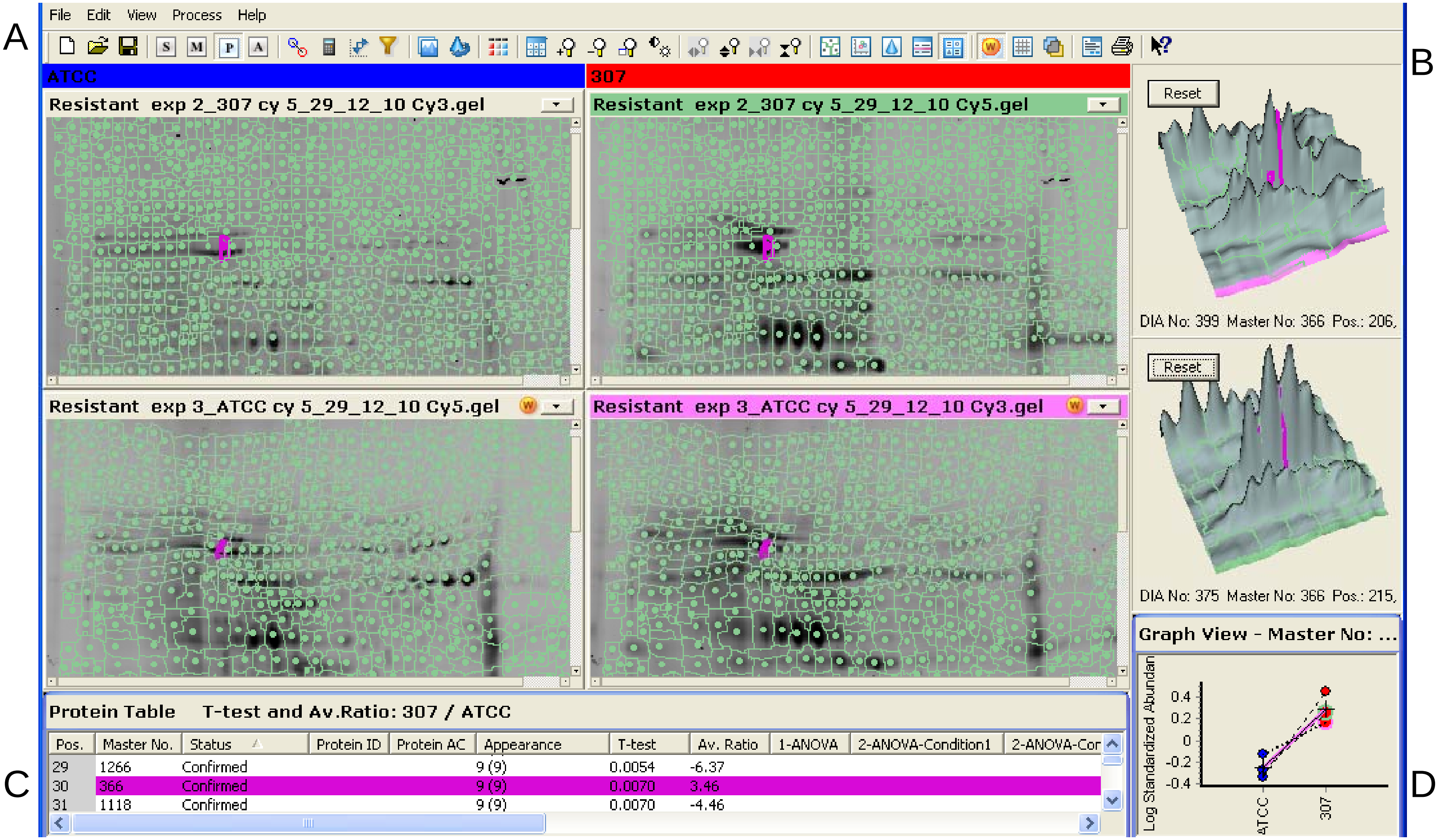

Figure S2.18: Representation of comparative DeCyder gel analysis of normalized gel image of native strain ATCC with resistant strain RS 307 using BVA module. All the combined results are displayed for upregulated master spot no. 366 of master gel.

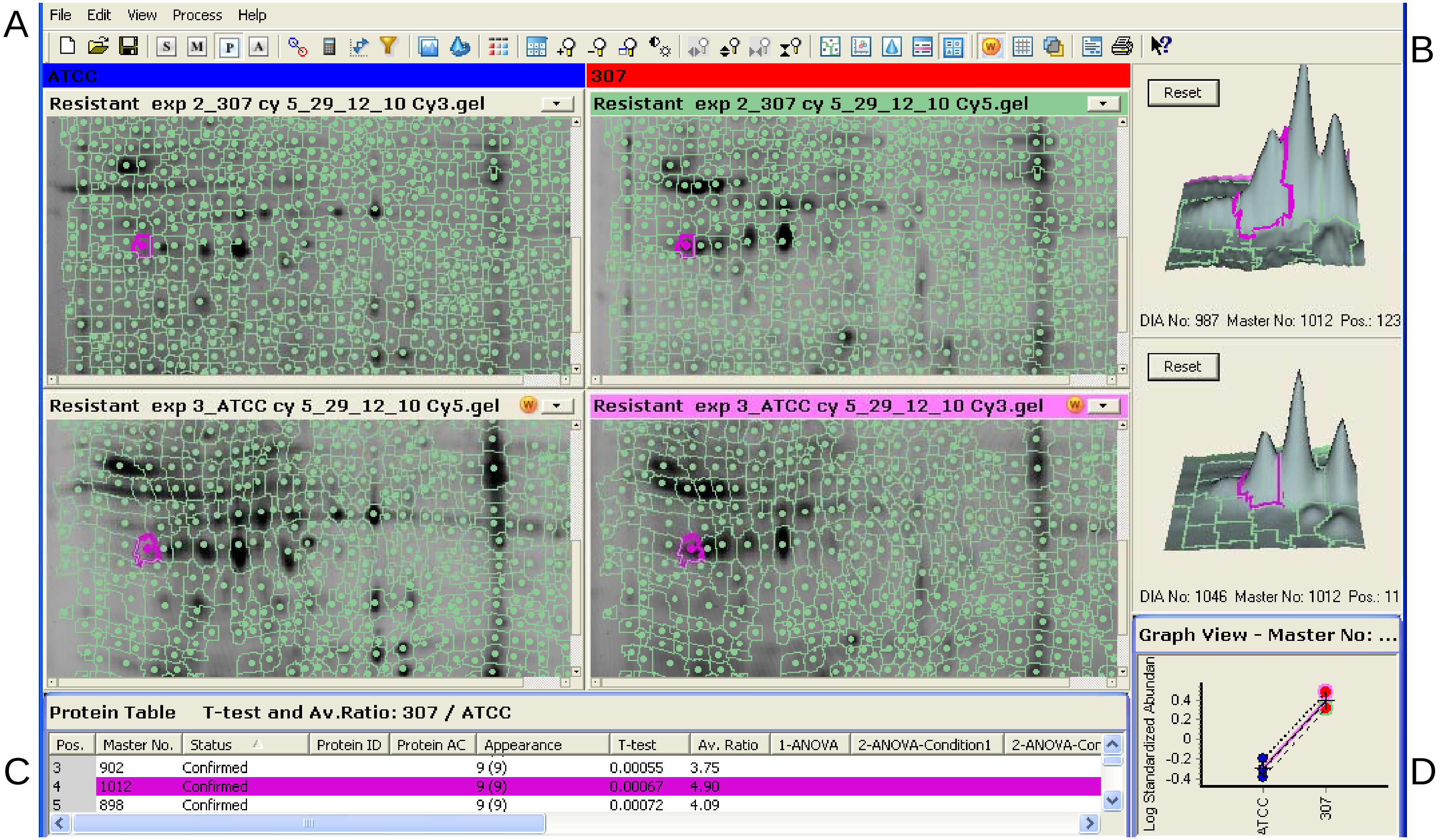

Figure S2.19: Representation of comparative DeCyder gel analysis of normalized gel image of native strain ATCC with resistant strain RS 307 using BVA module. All the combined results are displayed for upregulated master spot no. 1012 of master gel.

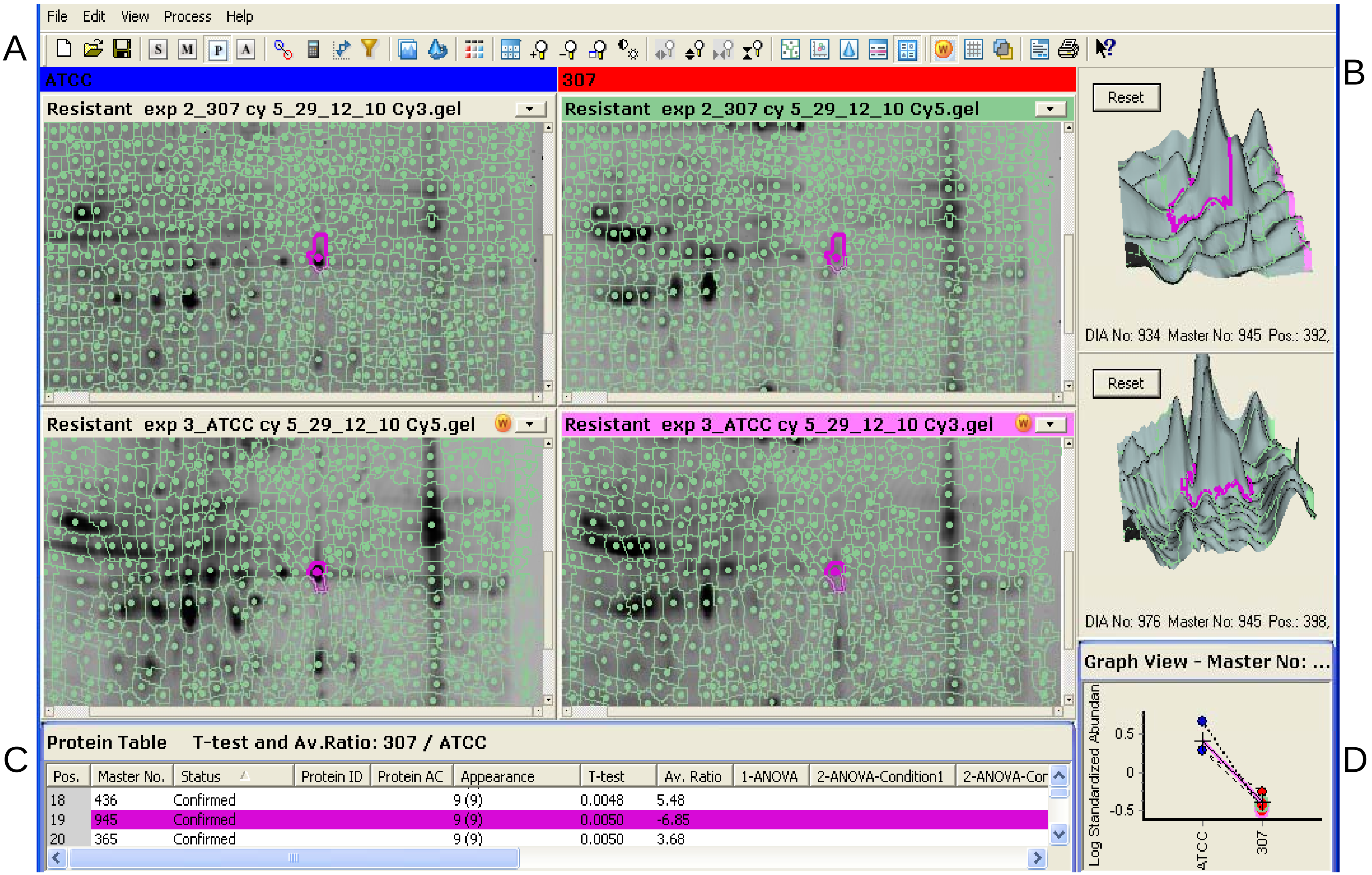

Figure S2.20: Representation of comparative DeCyder gel analysis of normalized gel image of native strain ATCC with resistant strain RS 307 using BVA module. All the combined results are displayed for downegulated master spot no. 945 of master gel.

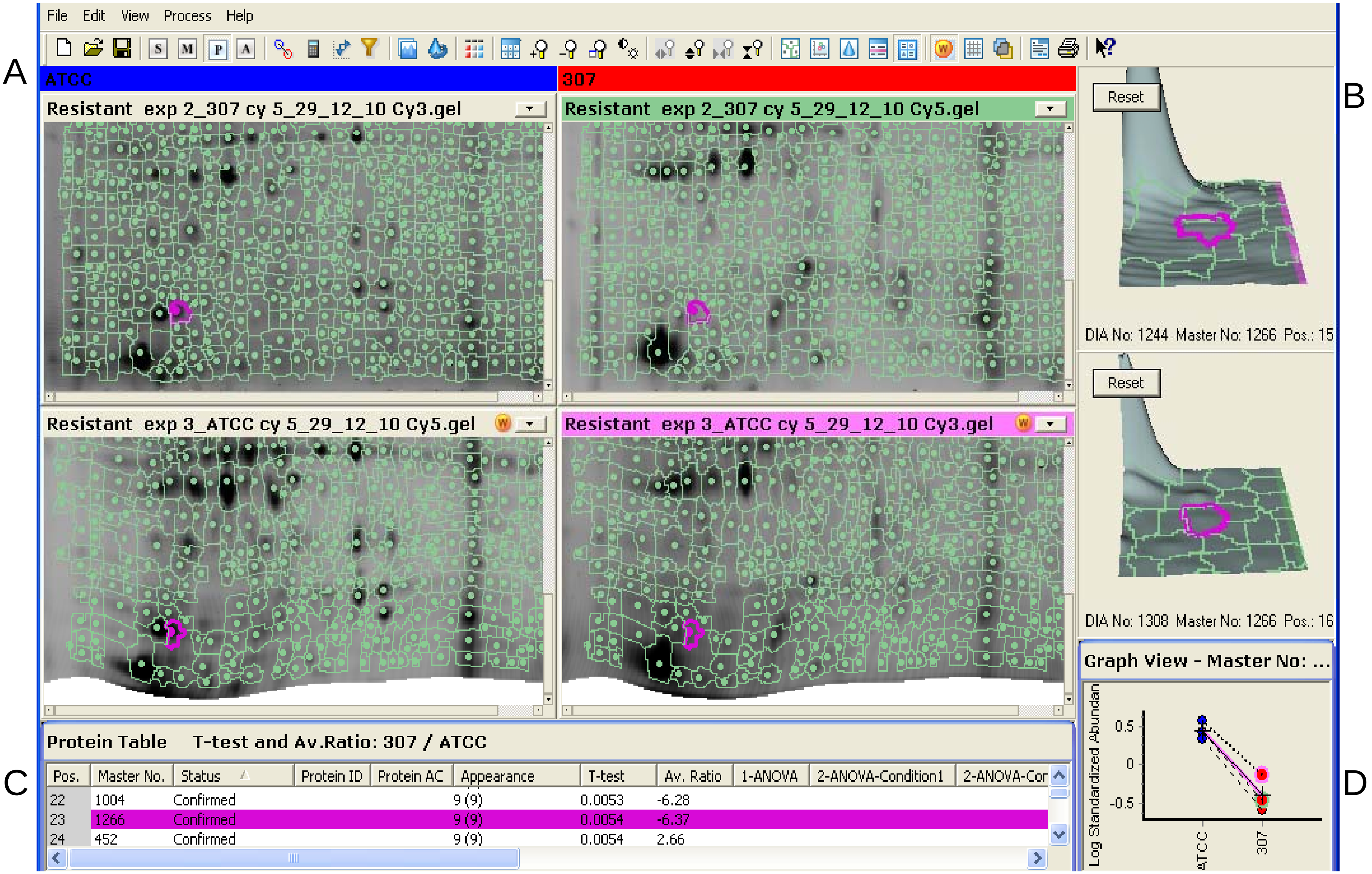

Figure S2.21: Representation of comparative DeCyder gel analysis of normalized gel image of native strain ATCC with resistant strain RS 307 using BVA module. All the combined results are displayed for downegulated master spot no. 1266 of master gel.

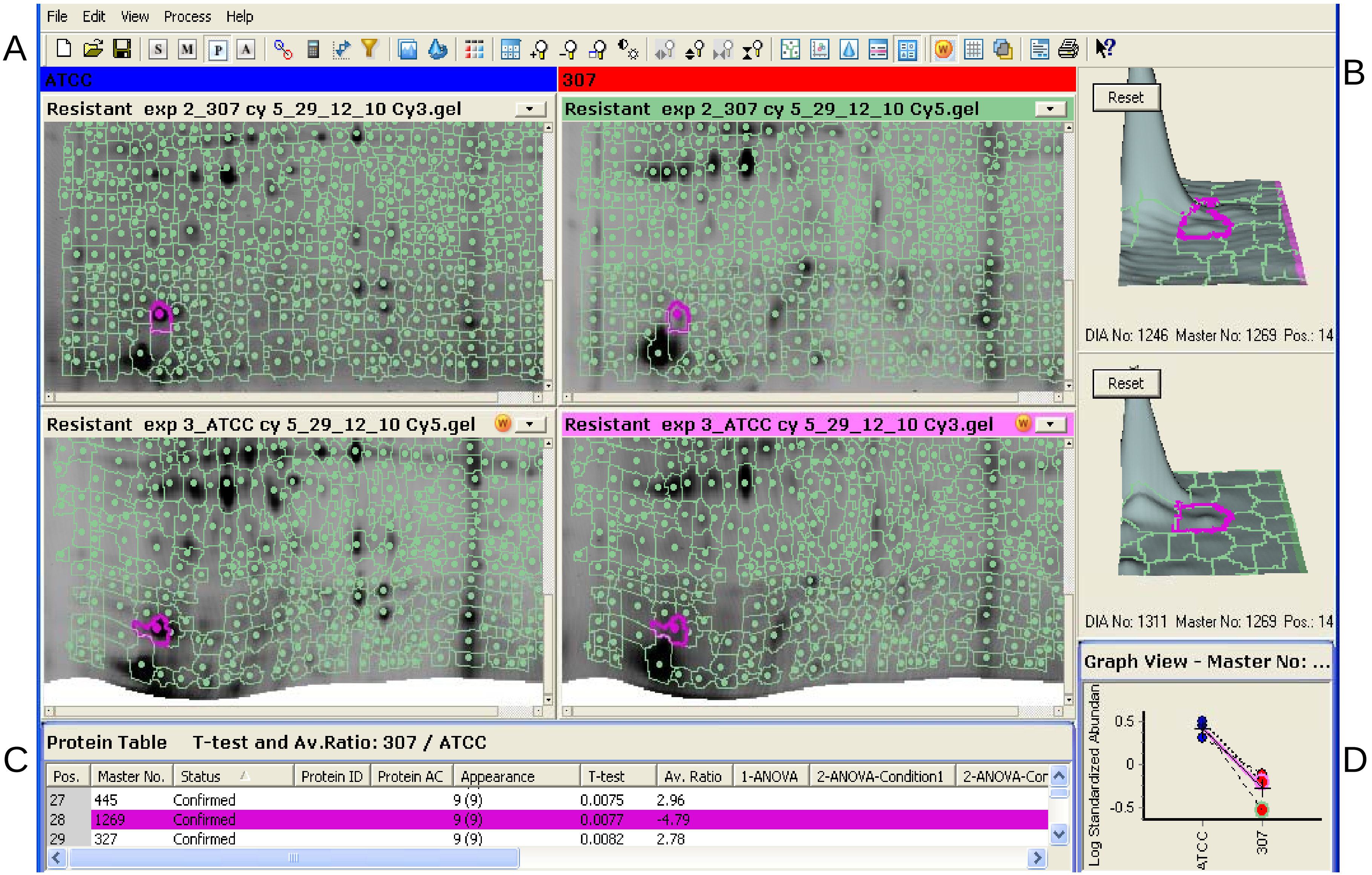

Figure S2.22: Representation of comparative DeCyder gel analysis of normalized gel image of native strain ATCC with resistant strain RS 307 using BVA module. All the combined results are displayed for downegulated master spot no. 1269 of master gel.

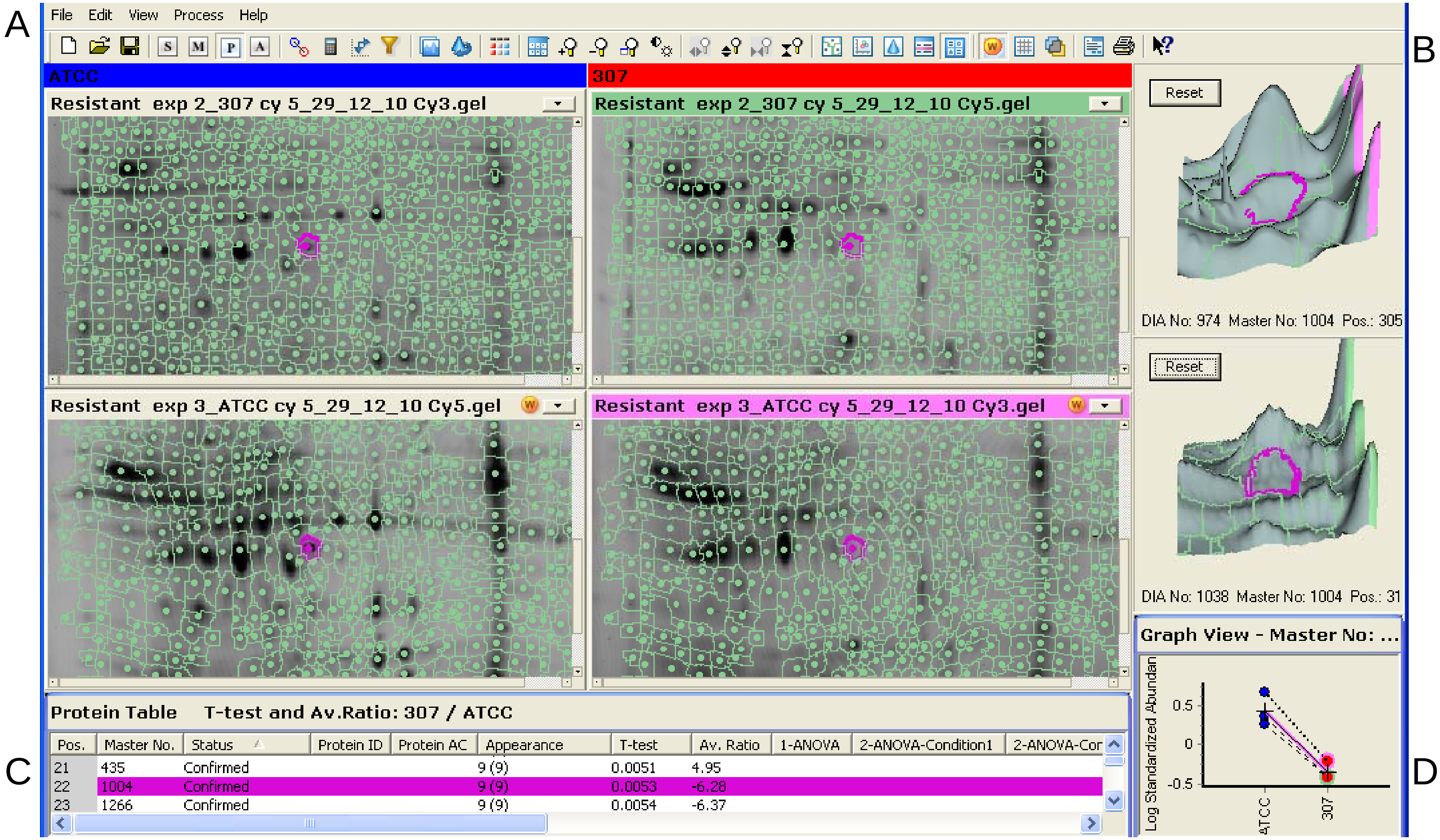

Figure S2.23: Representation of comparative DeCyder gel analysis of normalized gel image of native strain ATCC with resistant strain RS 307 using BVA module. All the combined results are displayed for downegulated master spot no. 1004 of master gel.
