# Supplementary material for: Comparative Proteomics of Inner Membrane Fraction from Carbapenem-Resistant Acinetobacter baumannii with a Reference Strain
Source: PLoS One. 2012 Jun 26;7(6):e39451. doi: 10.1371/journal.pone.0039451 (PMC3383706; doi:10.1371/journal.pone.0039451)
Supplement: Figure S3 — Progenesis analysis of RS307 DIGE results. (PDF) [file pone.0039451.s003.pdf]

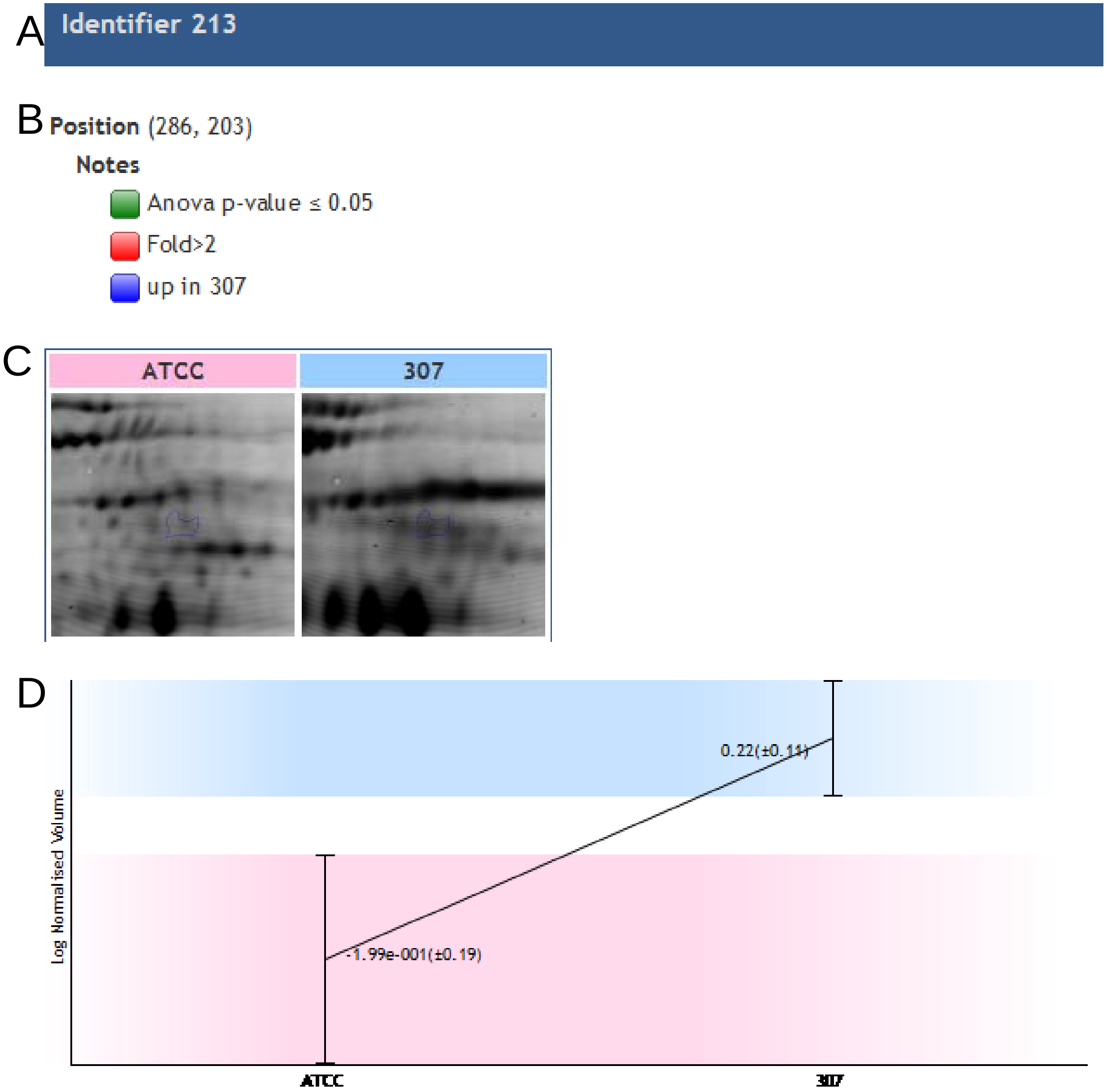

Figure S3.1 Representation of comparative Progenesis gel analysis of normalized gel image of native strain ATCC with high resistant strain RS 307 using Progenesis software. All the combined results are displayed for upregulated master spot no. 213 in Progenesis of master gel which is same as that of master number 490 in decyder. Panel A shows the master spot number in the gel. Panel B represents position, statistical measurement and fold change of the spot. Panel C shows location of spot in the gel. Pane D shows log normalized volume for same spot.

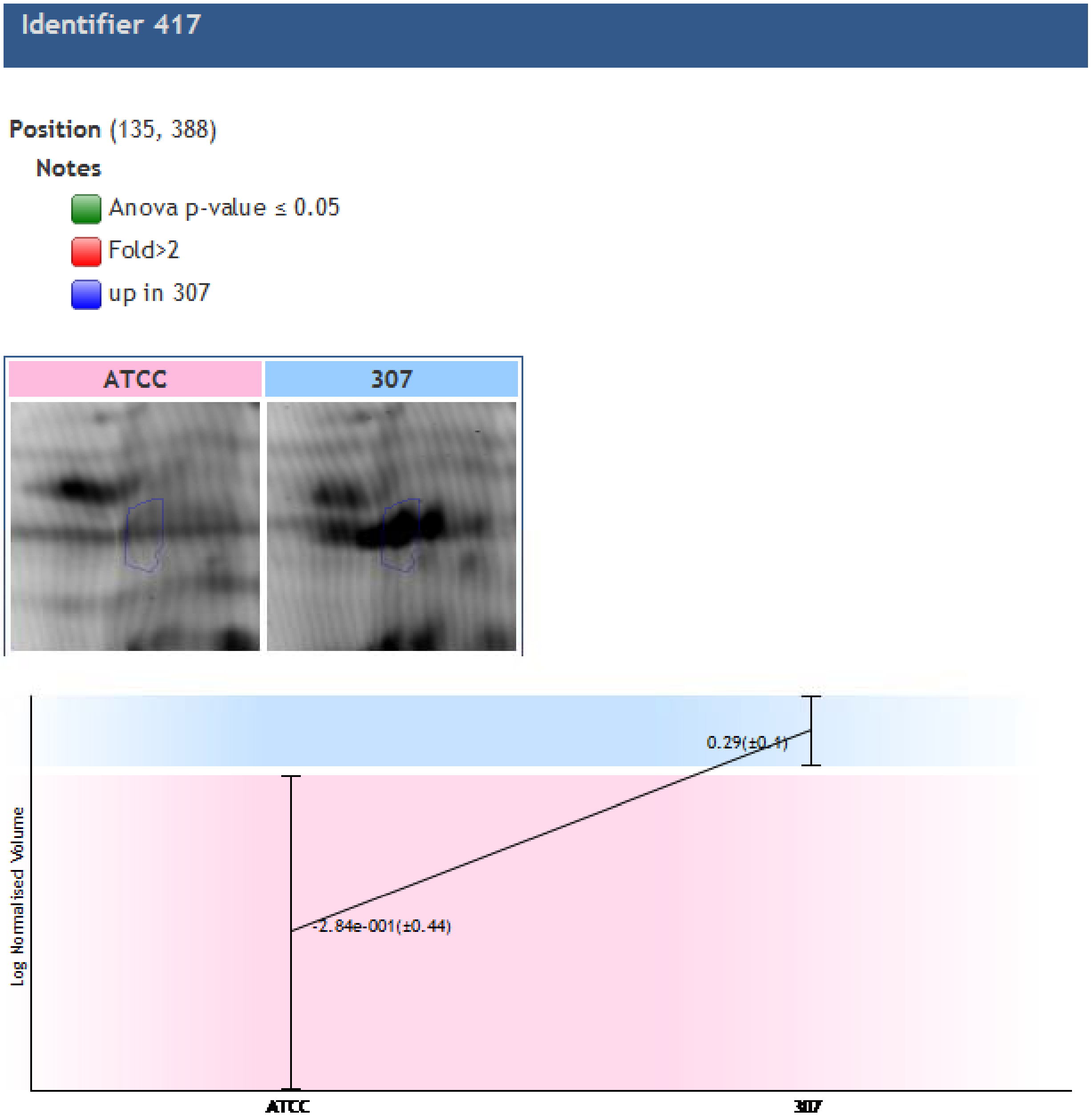

Figure S3.2. Representation of comparative Progenesis gel analysis of normalized gel image of native strain ATCC with high resistant strain RS 307 using Progenesis software. All the combined results are displayed for upregulated master spot no.417 in Progenesis of master gel which is same as that of master number 904 in decyder

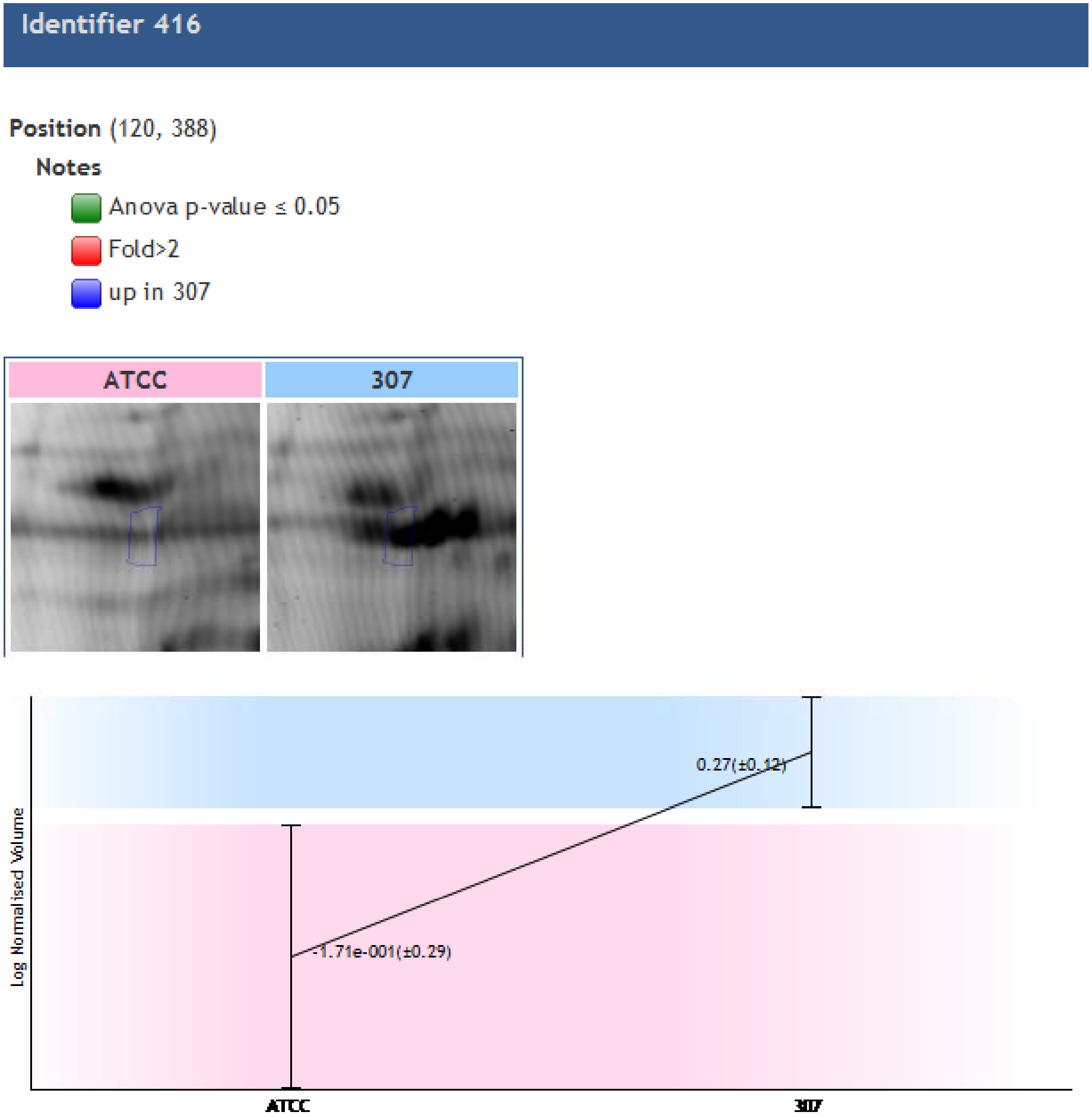

Figure S3.3 Representation of comparative Progenesis gel analysis of normalized gel image of native strain ATCC with high resistant strain RS 307 using Progenesis software. All the combined results are displayed for upregulated master spot no 416 in Progenesis of master gel which is same as that of master number 902 in decyder.

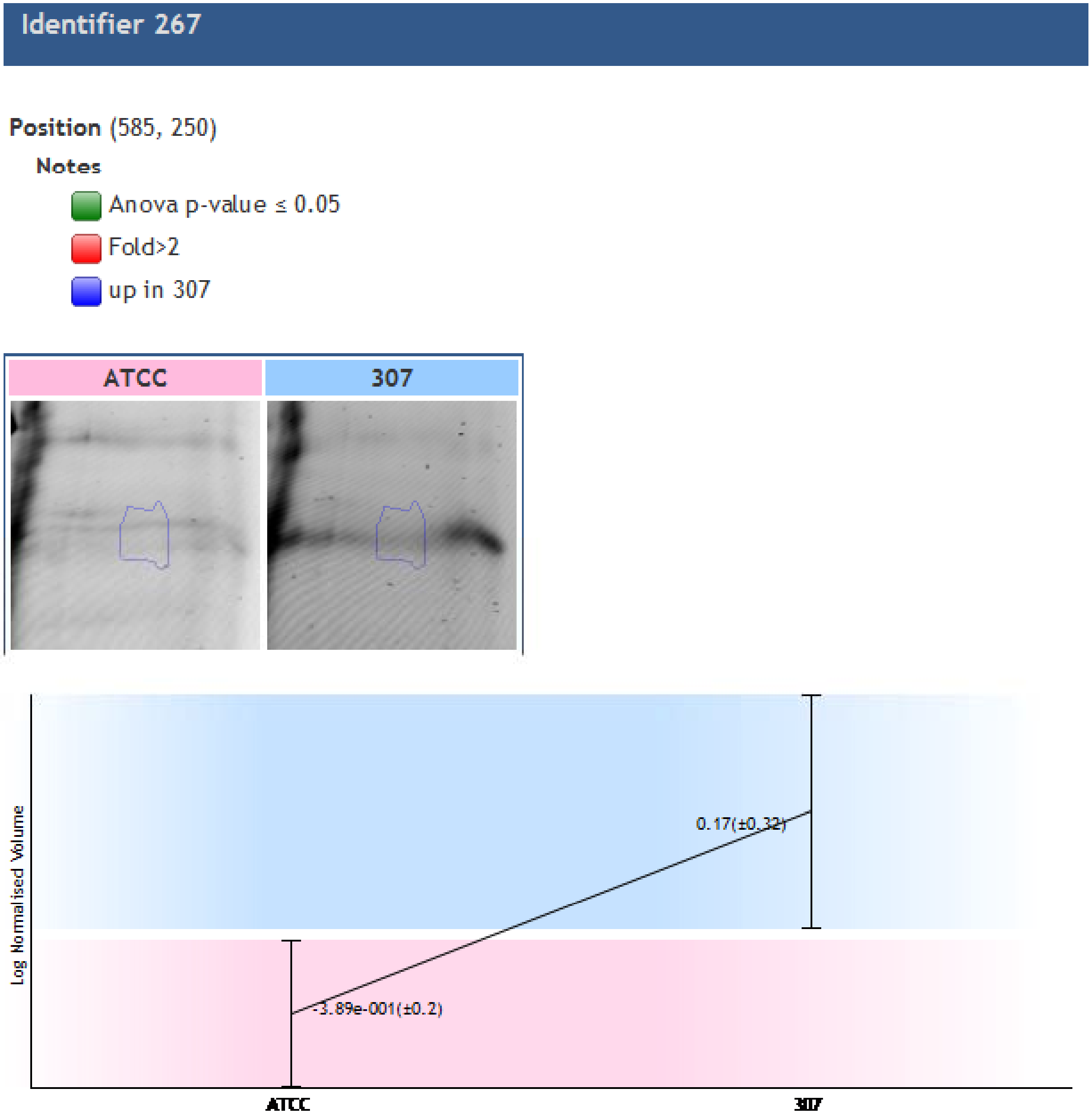

Figure S3.4 Representation of comparative Progenesis gel analysis of normalized gel image of native strain ATCC with high resistant strain RS 307 using Progenesis software. All the combined results are displayed for upregulated master spot no 267 in Progenesis of master gel which is same as that of master number 603 in decyder.

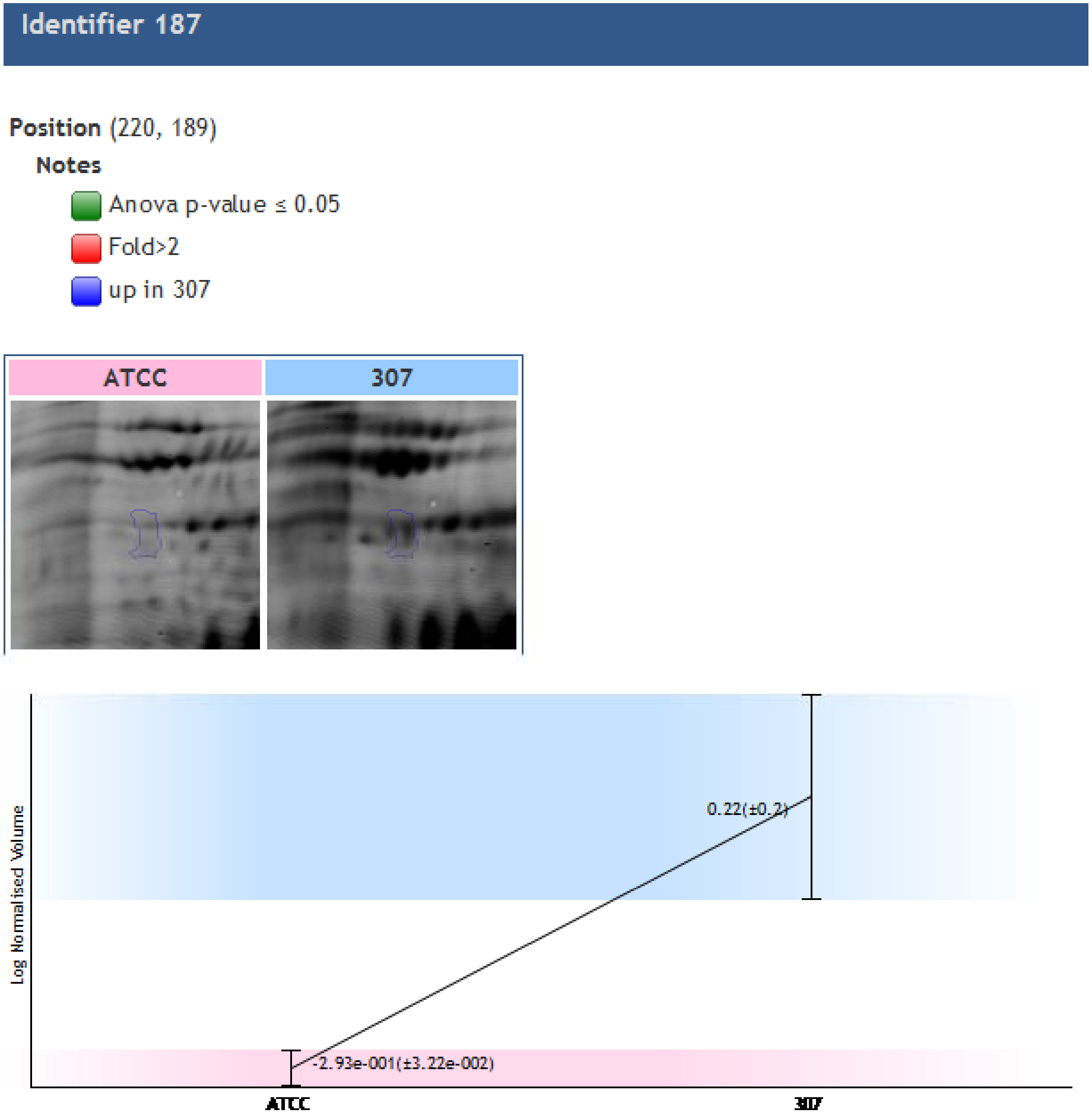

Figure S3.5 Representation of comparative Progenesis gel analysis of normalized gel image of native strain ATCC with high resistant strain RS 307 using Progenesis software. All the combined results are displayed for upregulated master spot no 187 in Progenesis of master gel which is same as that of master number 455 in decyder.

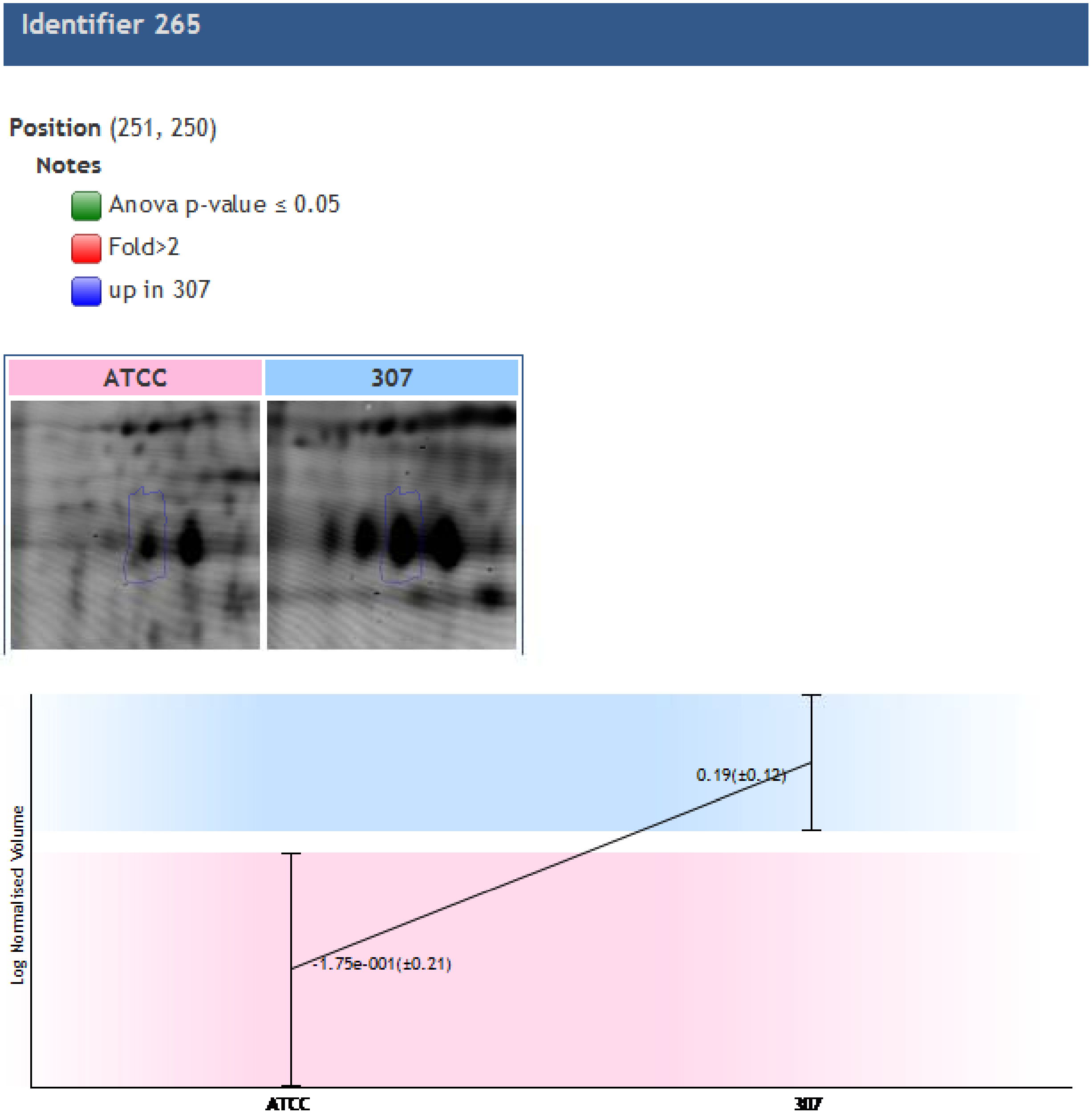

Figure S3.6 Representation of comparative Progenesis gel analysis of normalized gel image of native strain ATCC with high resistant strain RS 307 using Progenesis software. All the combined results are displayed for upregulated master spot no 265 in Progenesis of master gel which is same as that of master number 595 in decyder.

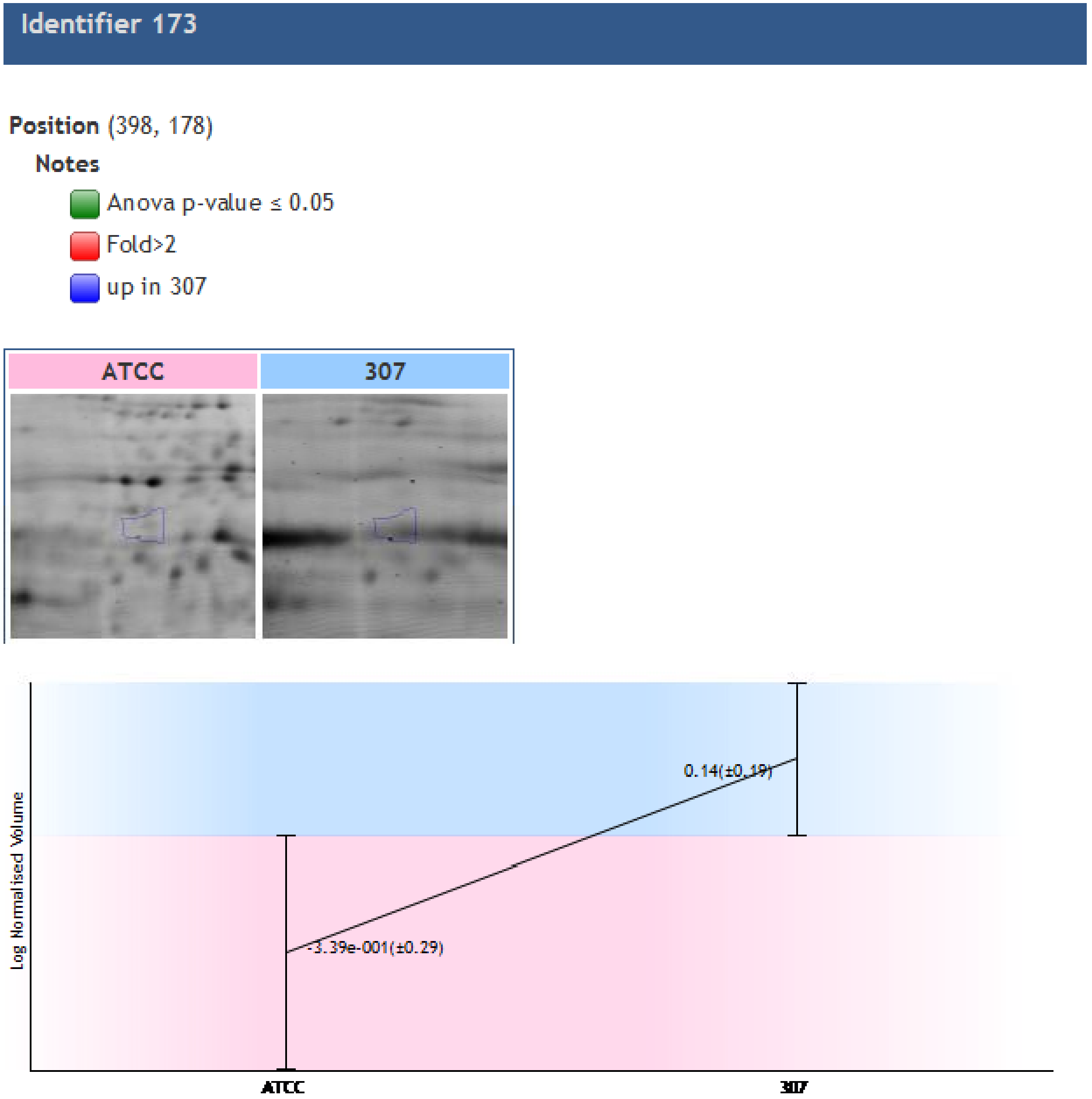

Figure S3.7 Representation of comparative Progenesis gel analysis of normalized gel image of native strain ATCC with high resistant strain RS 307 using Progenesis software. All the combined results are displayed for upregulated master spot no 173 in Progenesis of master gel which is same as that of master number 428 in decyder.

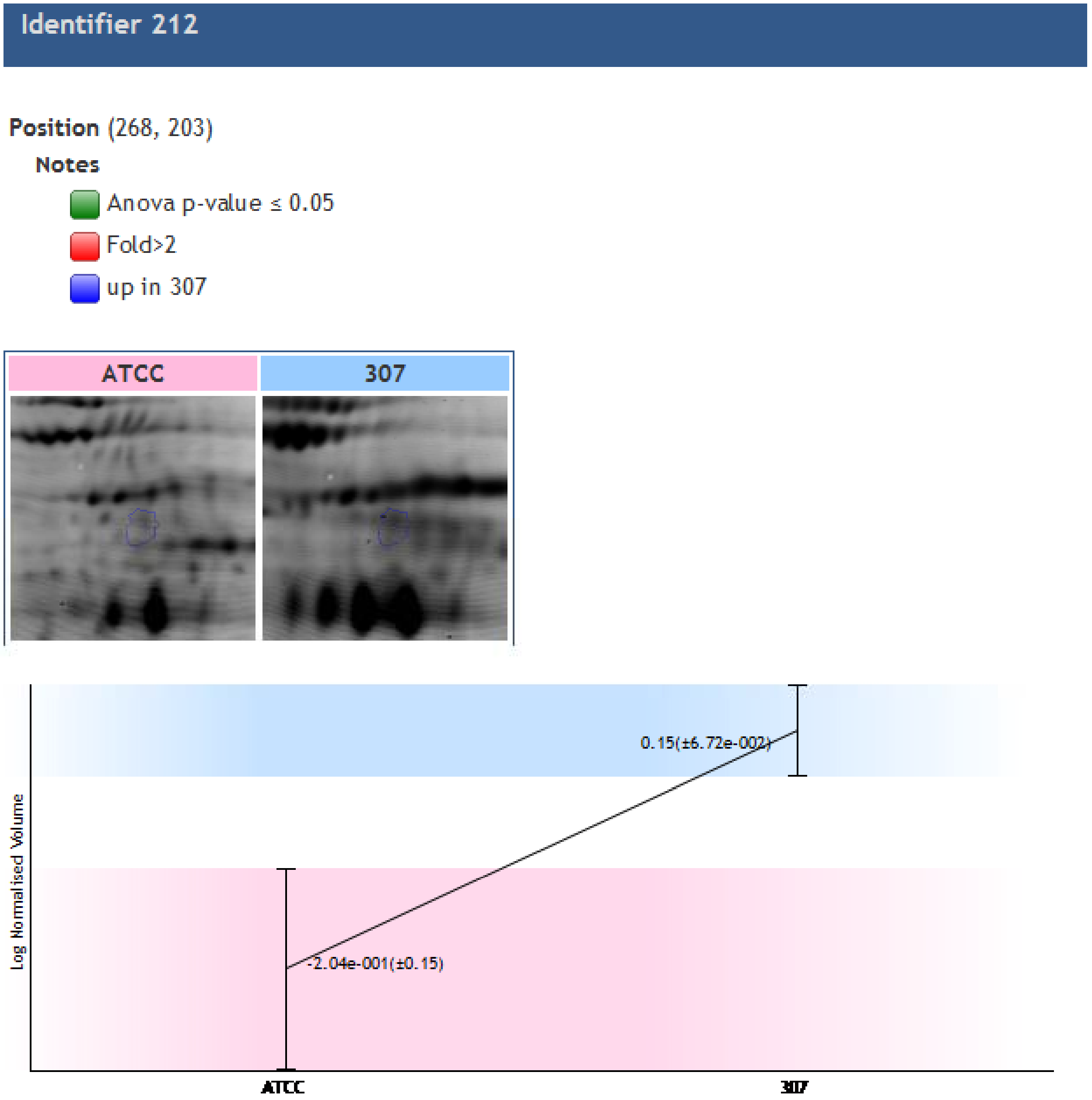

Figure S3.8 Representation of comparative Progenesis gel analysis of normalized gel image of native strain ATCC with high resistant strain RS 307 using Progenesis software. All the combined results are displayed for upregulated master spot no 212 in Progenesis of master gel which is same as that of master number 489 in decyder.

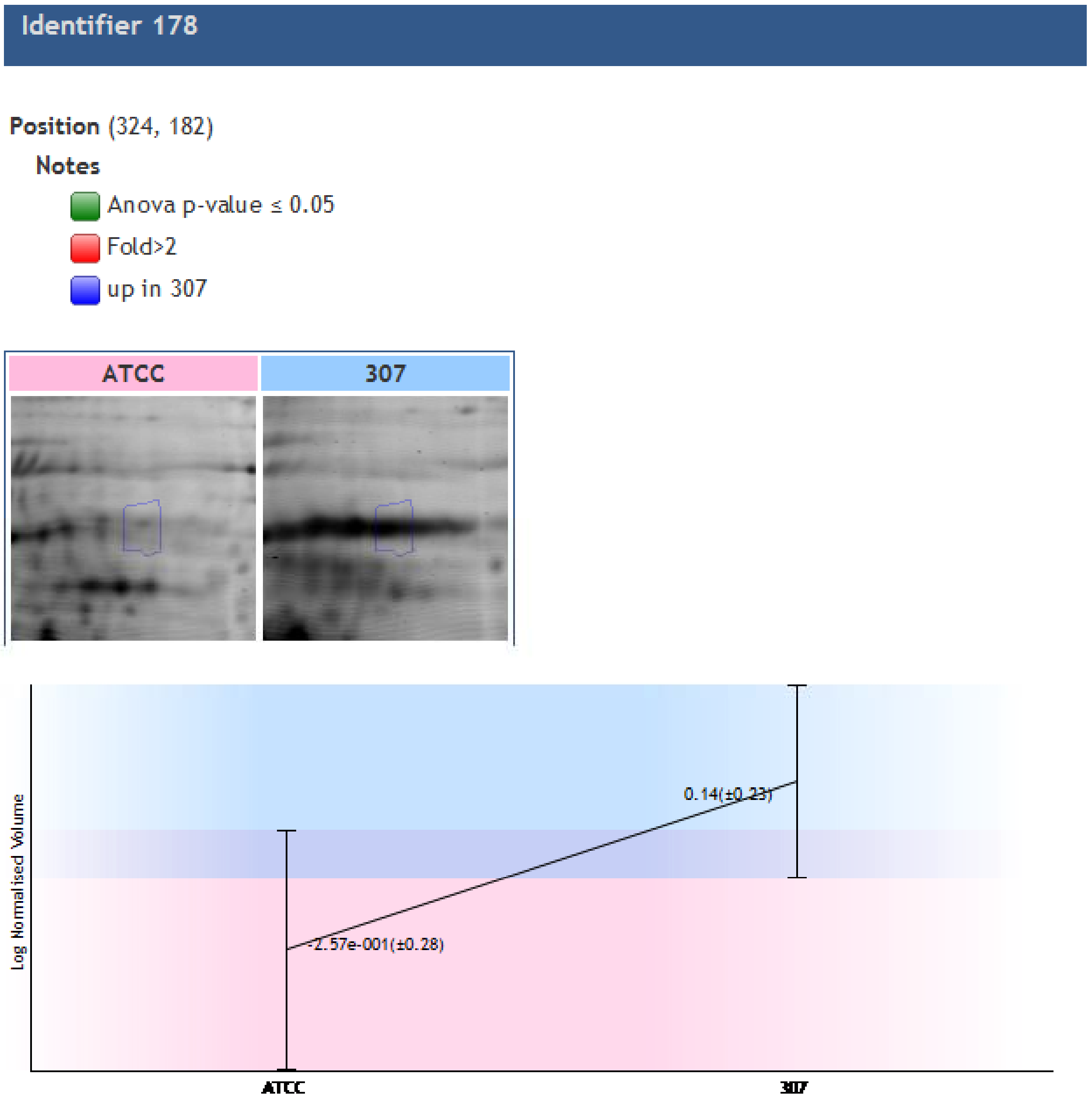

Figure S3.9 Representation of comparative Progenesis gel analysis of normalized gel image of native strain ATCC with high resistant strain RS 307 using Progenesis software. All the combined results are displayed for upregulated master spot no. 178 in Progenesis of master gel which is same as that of master number 436 in decyder.

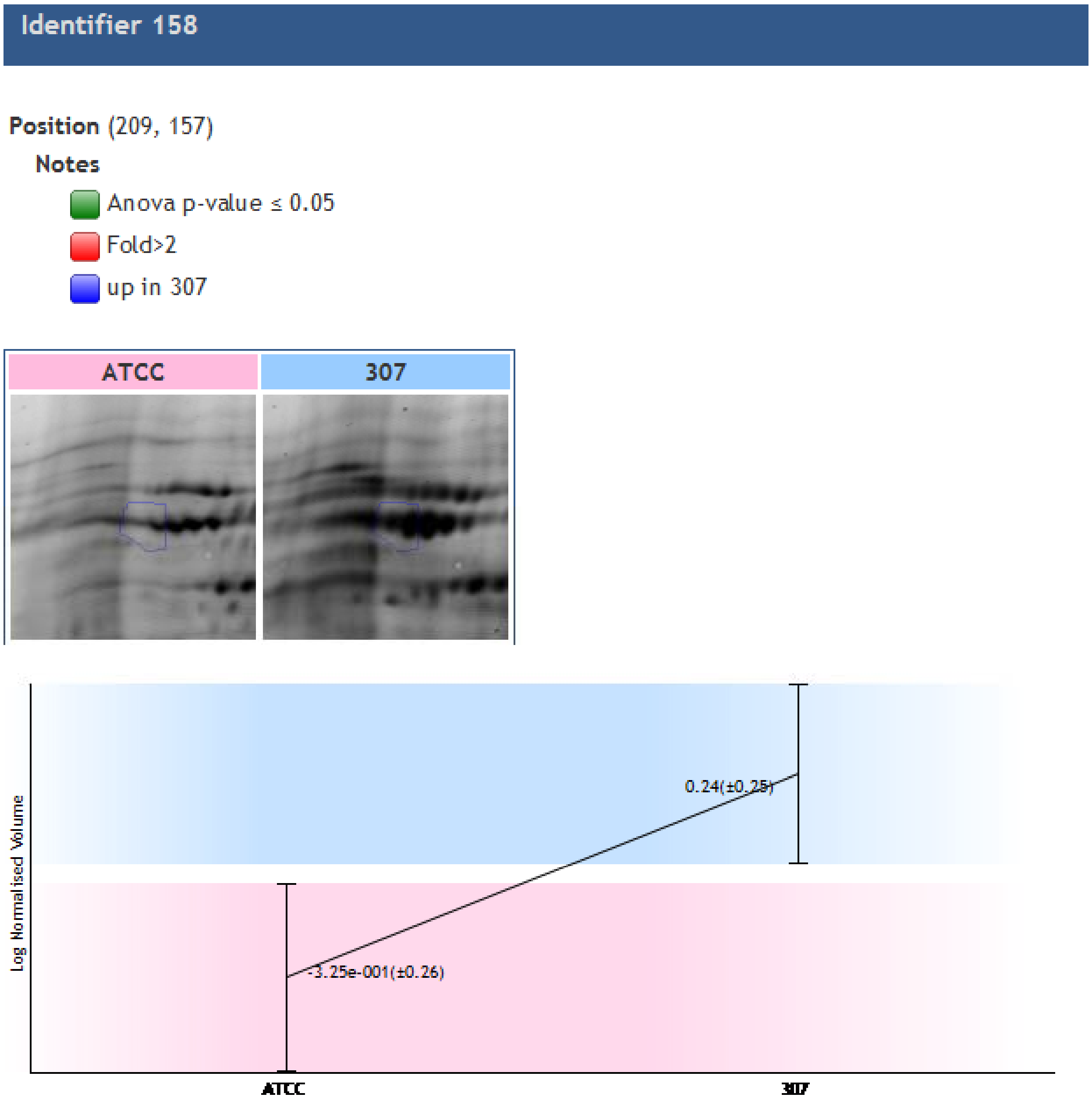

Figure S3.10 Representation of comparative Progenesis gel analysis of normalized gel image of native strain ATCC with high resistant strain RS 307 using Progenesis software. All the combined results are displayed for upregulated master spot no. 158 in Progenesis of master gel which is same as that of master number 365 in decyder.

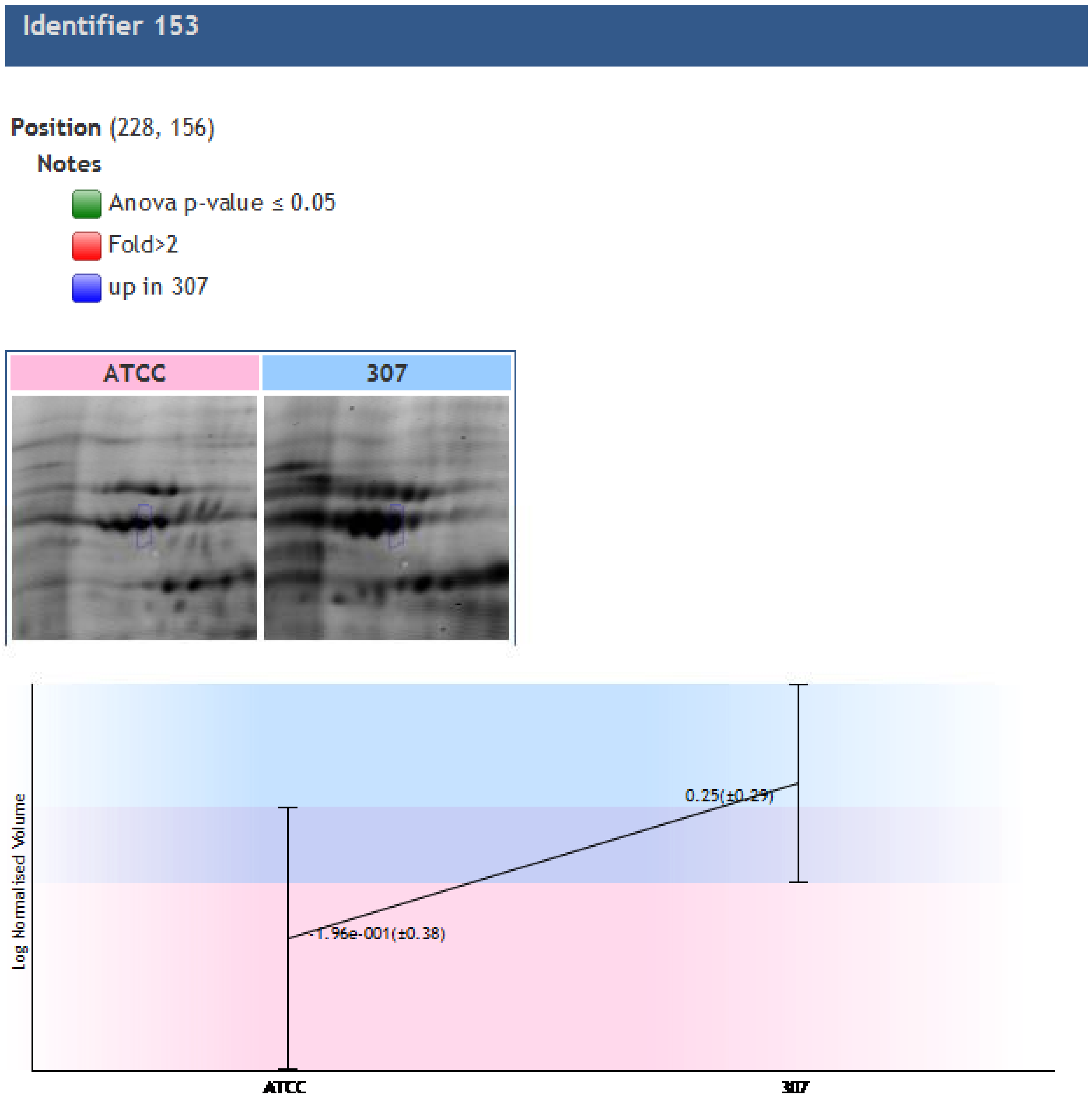

Figure S3.11 Representation of comparative Progenesis gel analysis of normalized gel image of native strain ATCC with high resistant strain RS 307 using Progenesis software. All the combined results are displayed for upregulated master spot no. 153 in Progenesis of master gel which is same as that of master number 366 in decyder.

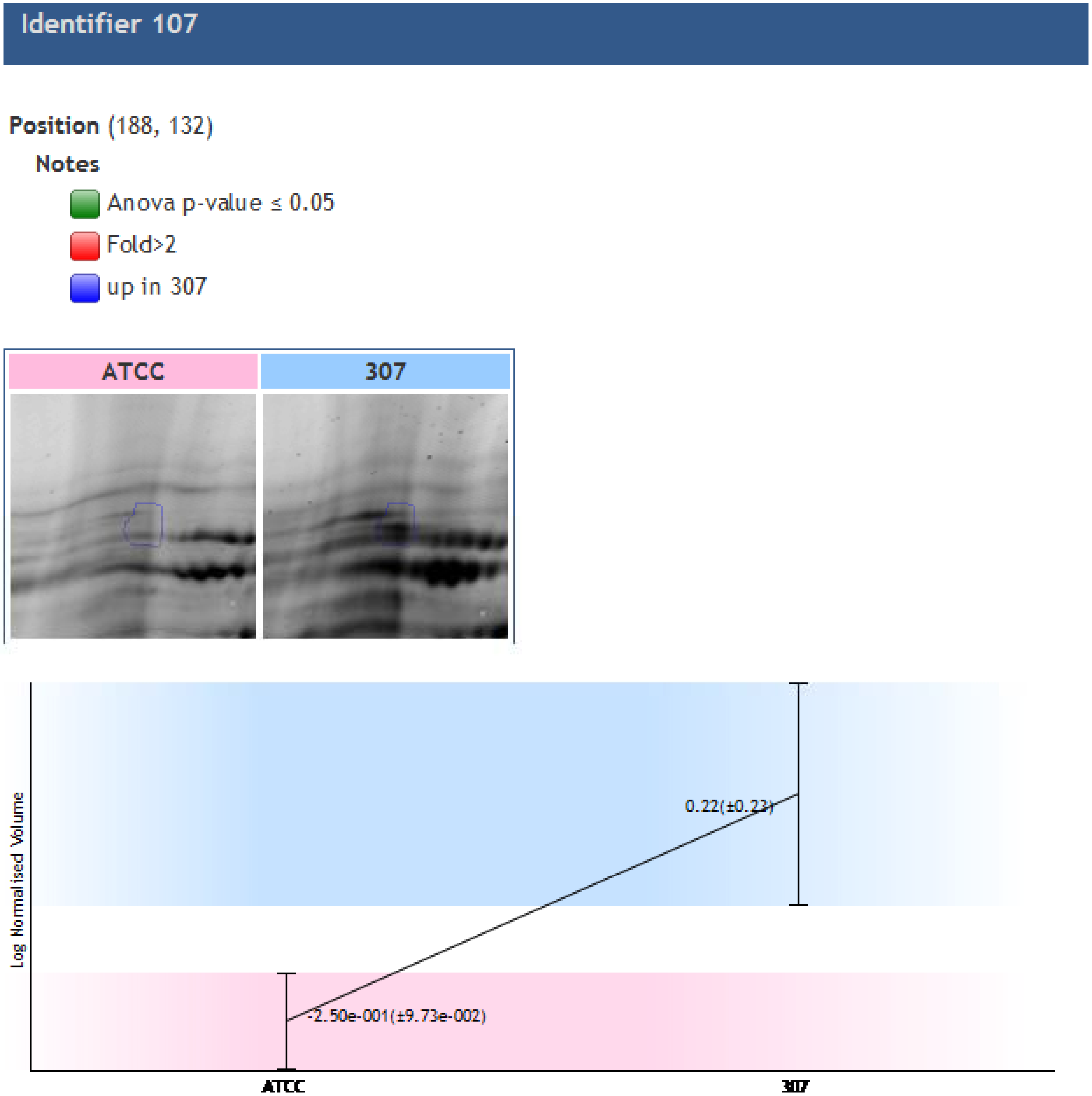

Figure S3.12 Representation of comparative Progenesis gel analysis of normalized gel image of native strain ATCC with high resistant strain RS 307 using Progenesis software. All the combined results are displayed for upregulated master spot no 107 in Progenesis of master gel which is same as that of master number 327 in decyder.

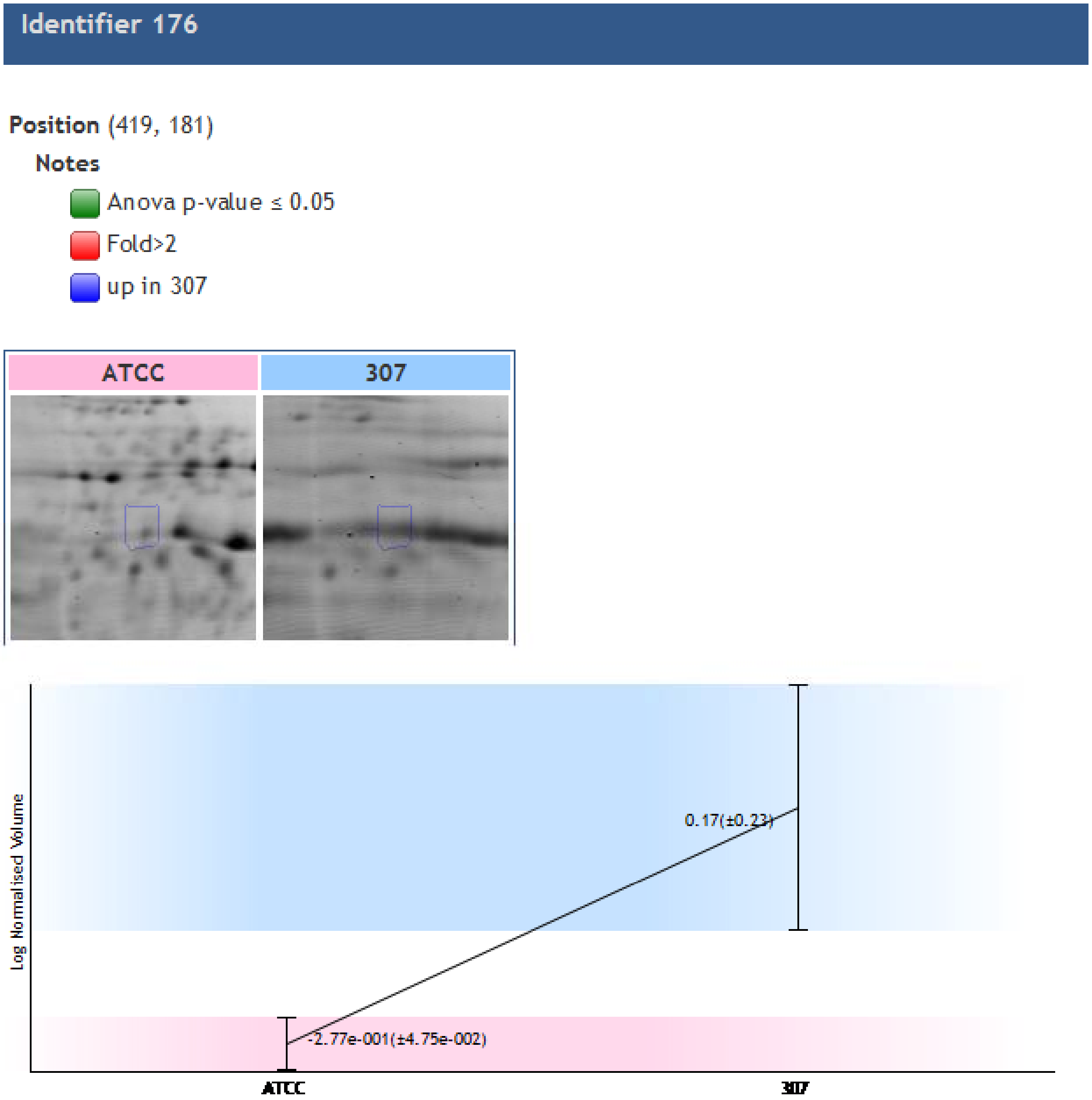

Figure S3.13 Representation of comparative Progenesis gel analysis of normalized gel image of native strain ATCC with high resistant strain RS 307 using Progenesis software. All the combined results are displayed for upregulated master spot no. 176 in Progenesis of master gel which is same as that of master number 432 in decyder.

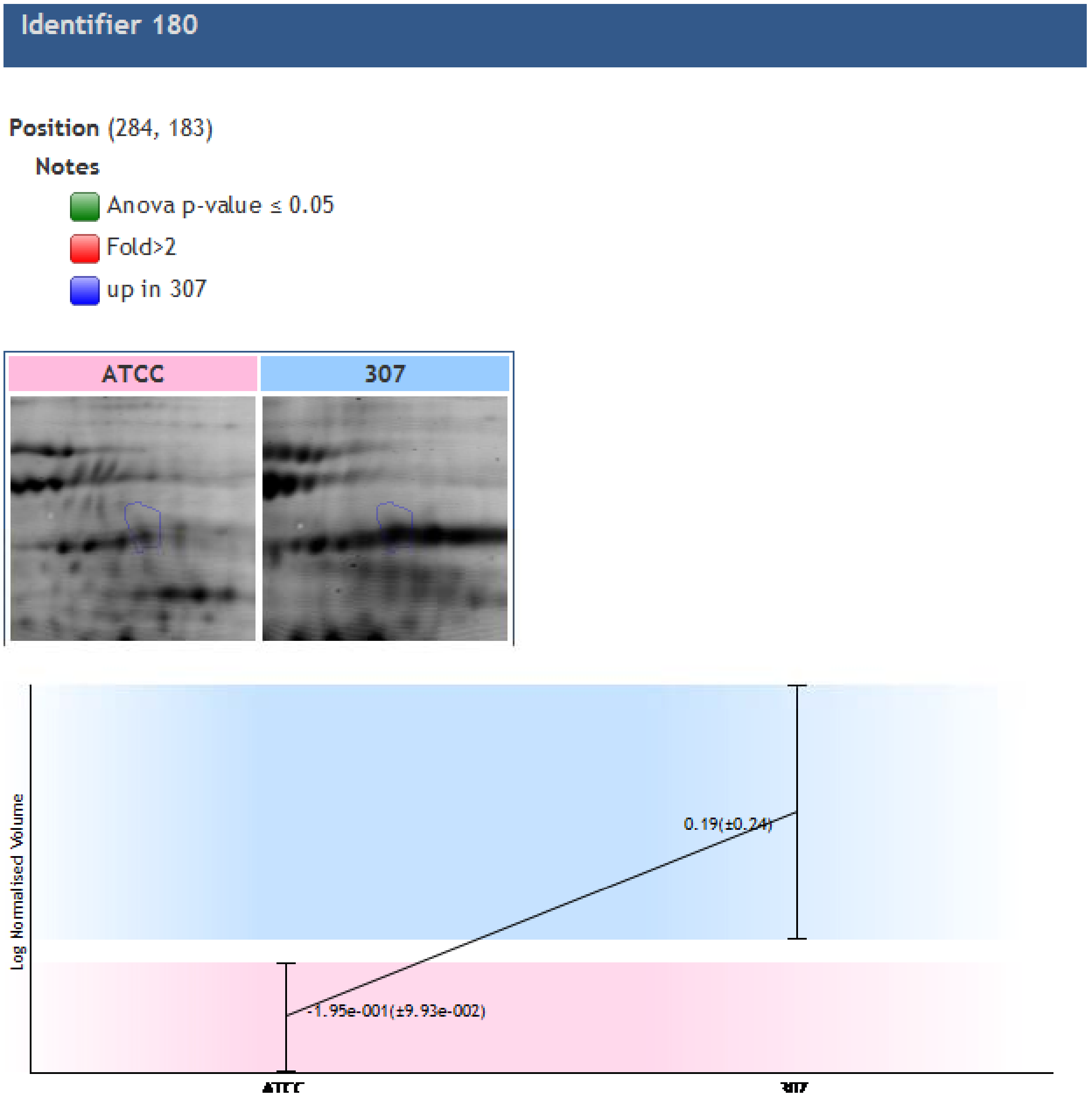

Figure S3.14 Representation of comparative Progenesis gel analysis of normalized gel image of native strain ATCC with high resistant strain RS 307 using Progenesis software. All the combined results are displayed for upregulated master spot no 180 in Progenesis of master gel which is same as that of master number 434 in decyder.

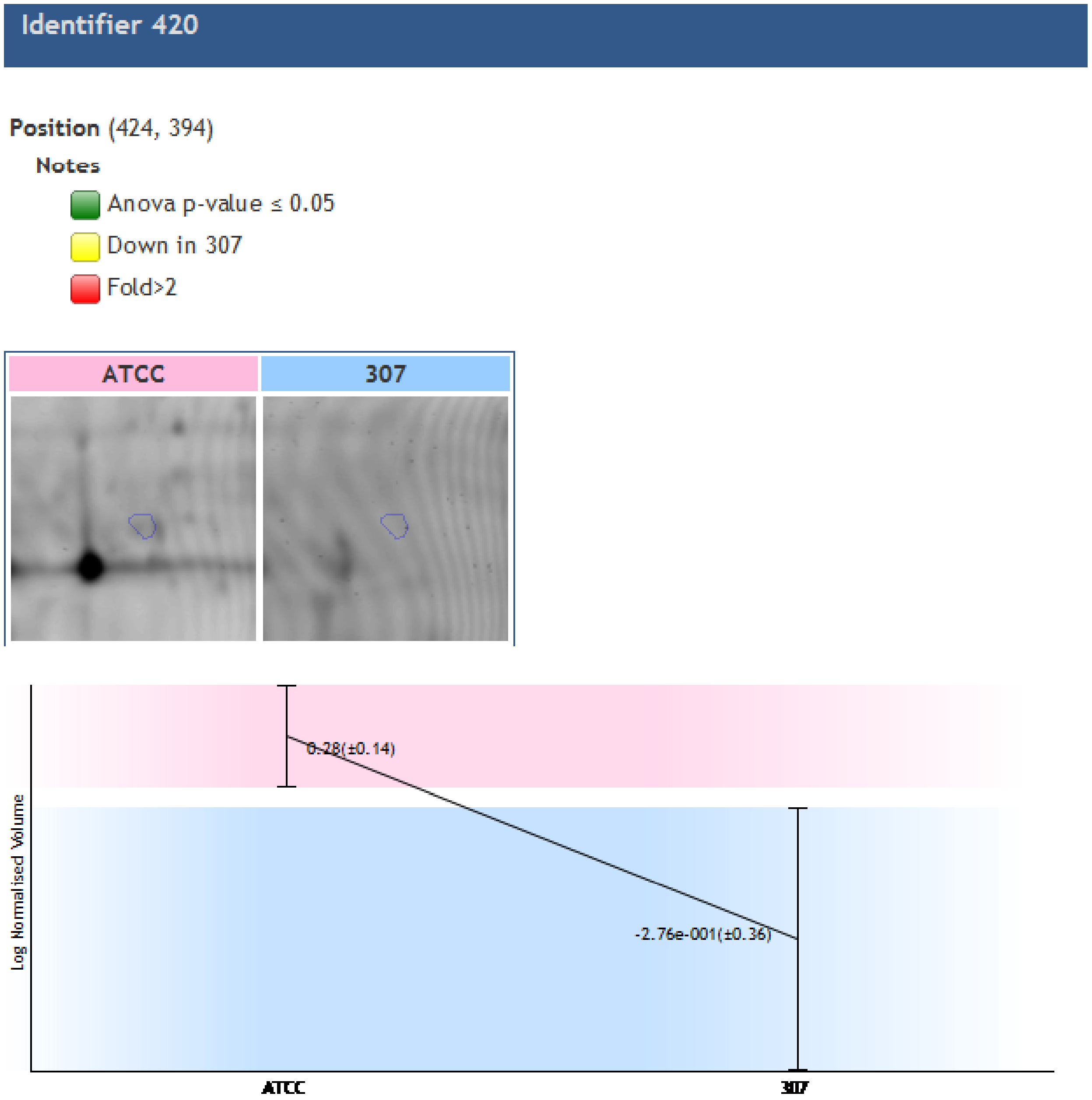

Figure S3.15 Representation of comparative Progenesis gel analysis of normalized gel image of native strain ATCC with high resistant strain RS 307 using Progenesis software. All the combined results are displayed for downregulated master spot no. 420 in Progenesis of master gel which is same as that of master number 945 in decyder.

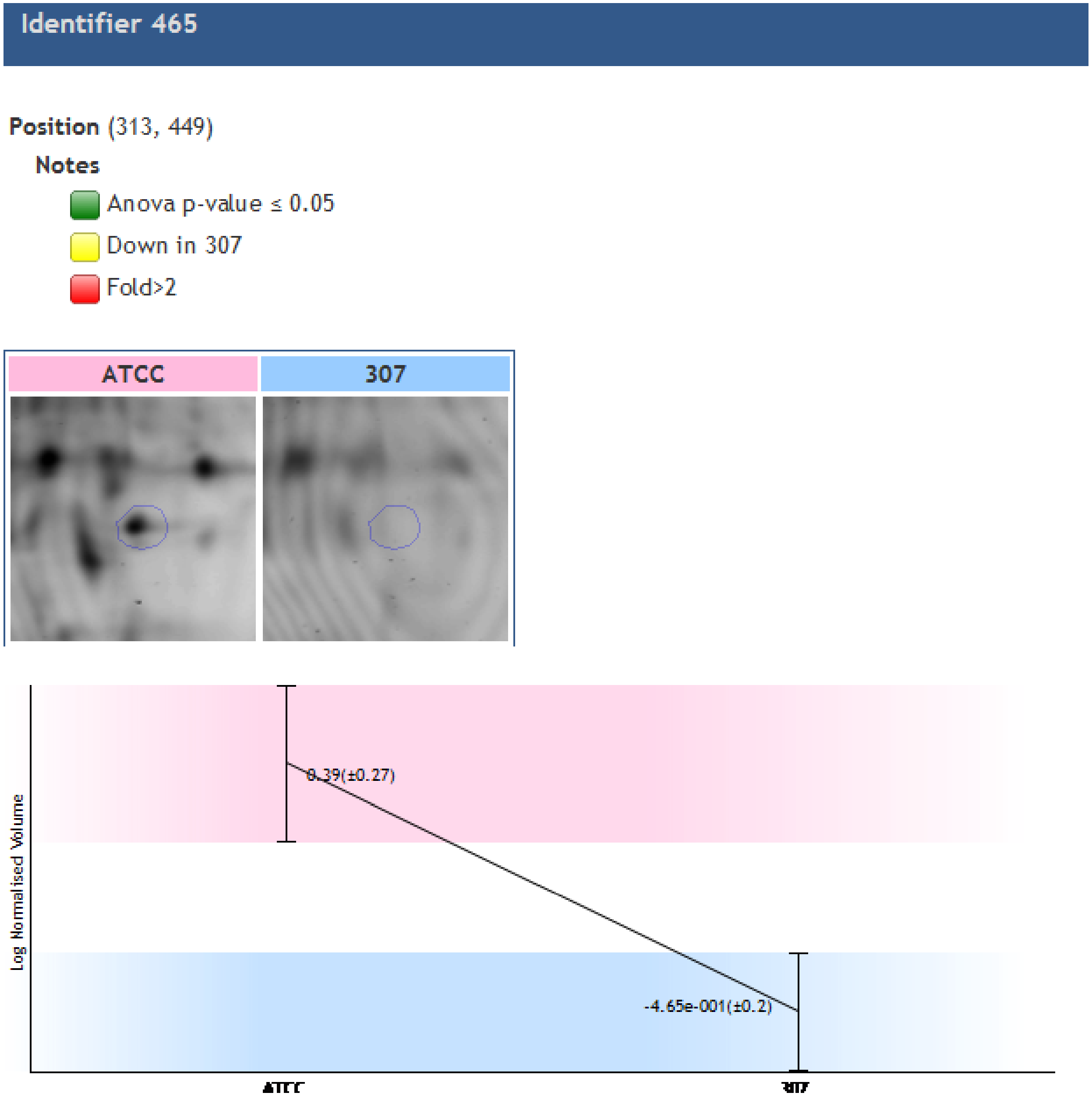

Figure S3.16 Representation of comparative Progenesis gel analysis of normalized gel image of native strain ATCC with high resistant strain RS 307 using Progenesis software. All the combined results are displayed for downregulated master spot no. 465 in Progenesis of master gel which is same as that of master number 1004 in decyder.

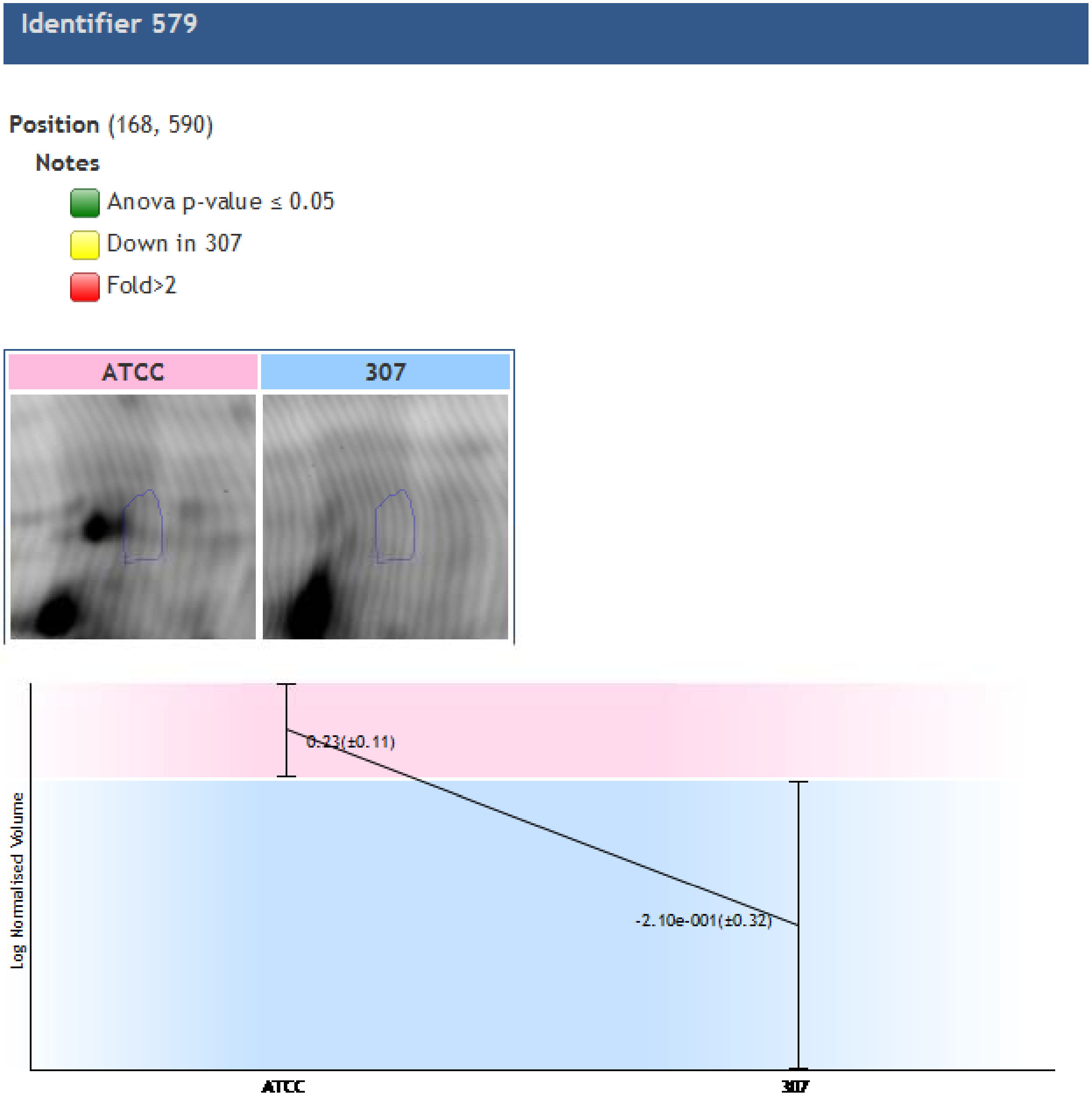

Figure S3.17 Representation of comparative Progenesis gel analysis of normalized gel image of native strain ATCC with high resistant strain RS 307 using Progenesis software. All the combined results are displayed for downregulated master spot no. 579 in Progenesis of master gel which is same as that of master number 1266 in decyder.

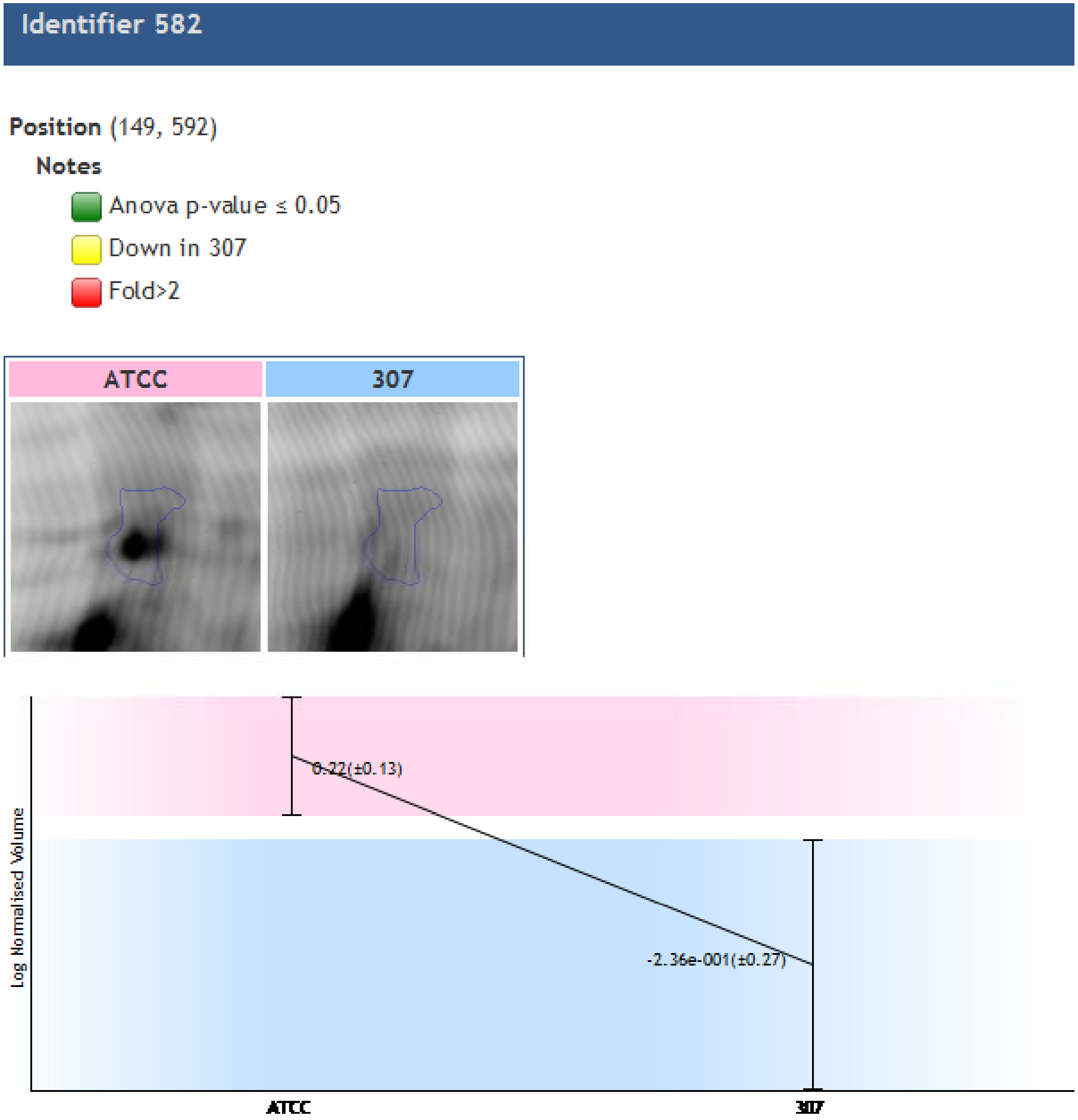

Figure S3.18 Representation of comparative Progenesis gel analysis of normalized gel image of native strain ATCC with high resistant strain RS 307 using Progenesis software. All the combined results are displayed for downregulated master spot no. 582 in Progenesis of master gel which is same as that of master number 1269 in decyder.
