# Supplementary material for: Comparative Proteomics of Inner Membrane Fraction from Carbapenem-Resistant Acinetobacter baumannii with a Reference Strain
Source: PLoS One. 2012 Jun 26;7(6):e39451. doi: 10.1371/journal.pone.0039451 (PMC3383706; doi:10.1371/journal.pone.0039451)
Supplement: Figure S4 — Mascot search results for differentially expressed proteins in inner membrane fraction of RS307. (PDF) [file pone.0039451.s004.pdf]

Mascot Search Results

Protein View

Match to: **gi|7258342** Score: **156**
beta-lactamase class C [Acinetobacter baumannii]
Found in search of C:\Temp\mas3C.tmp

Nominal mass (M_r): **43237**; Calculated pI value: **9.49**

NCBI BLAST search of gi|7258342 against nr
Unformatted sequence string for pasting into other applications

Taxonomy: Acinetobacter baumannii
Links to retrieve other entries containing this sequence from NCBI Entrez:
gi|37699616 from Acinetobacter baumannii

Fixed modifications: Carbamidomethyl (C)
Variable modifications: Oxidation (M)
Cleavage by Trypsin: cuts C-term side of KR unless next residue is P
Sequence Coverage: **10%**

Matched peptides shown in **Bold Red**

```
  1 MRFKKISCLL LSPLFIFSTS IYAGNTPKDQ EIKKLVDQNF KPLLEKYDVP
 51 GMAVGVIQNN KKYEMYYGLQ SVQDKKAVNS STIFELGSVS KLFTATAGGY
101 AKNKGKISFD DTPGKYWKEL KNTPIDQVNL LQLATYTSGN LALQFPDEVK
151 TDQQVLTFFK DWKPKNSIGE YRQYSNPSIG LFGKVVALSM NKPFDQVLEK
201 TIFPALGLKH SYVNVPKTQM QNYAFGYNQE NQPIRVNPGP LGAPAYGVKS
251 TLPDMLSFIH ANLNPQKYPA DIQRAINETH QGRYQVNTMY QALGWEEFSY
301 PATLQTLLDS NSEQIVMKPN KVTAISKEPS VKMYHKTGST NRFGTYVVFI
351 PKENIGLVML TNKRIPNEER IKAAYAVLNA IKK
```

Show predicted peptides also

Sort Peptides By ◉ Residue Number ○ Increasing Mass ○ Decreasing Mass

Start - End	Observed	Mr(expt)	Mr(calc)	Delta	Miss	Sequence
77 - 91	770.3636	1538.7126	1537.7988	0.9138	0	K.AVNSSTIFELGSVSK.L (Ions score 83)
201 - 209	480.2817	958.5488	958.5851	-0.0364	0	K.TIFPALGLK.H (Ions score 38)
250 - 267	681.3320	2040.9742	2041.0302	-0.0561	0	K.STLPDMLSFIHANLNPQK.Y Oxidation (M) (Ions score 34)

Error: try setting browser cache to automatic.

Error: try setting browser cache to automatic.

LOCUS CAB77444 383 aa linear BCT 15-APR-2005 DEFINITION beta-lactamase class C [Acinetobacter baumannii]. ACCESSION CAB77444 VERSION CAB77444.1 GI:7258342 DBSOURCE embl accession AJ009979.1 KEYWORDS . SOURCE Acinetobacter baumannii ORGANISM Acinetobacter baumannii Bacteria; Proteobacteria; Gammaproteobacteria; Pseudomonadales; Moraxellaceae; Acinetobacter; Acinetobacter calcoaceticus/baumannii complex. REFERENCE 1 AUTHORS Bou,G. and Mart inverted question markinez-Beltr inverted question markan,J. TITLE Cloning, nucleotide sequencing, and analysis of the gene encoding an AmpC beta-lactamase in Acinetobacter baumannii JOURNAL Antimicrob. Agents Chemother. 44 (2), 428-432 (2000) PUBMED 10639377 REFERENCE 2 (residues 1 to 383) AUTHORS Bou,G. TITLE Direct Submission JOURNAL Submitted (10-AUG-1999) Bou G., Microbiology, Hospital Ramon Y Cajal, Crta. Colmenar Viejo Km 9,1, Madrid/28034, SPAIN FEATURES Location/Qualifiers source 1..383 /organism="Acinetobacter baumannii" /isolate="RYC 52763/97" /db_xref="taxon:470" Protein 1..383 /product="beta-lactamase class C" /EC_number="3.5.2.6" Region 1..380 /region_name="AmpC" /note="Beta-lactamase class C and other penicillin binding proteins [Defense mechanisms]; COG1680" /db_xref="CDD:31866" Region 4..382 /region_name="Beta-lactamase" /note="Beta-lactamase; cl01009" /db_xref="CDD:194006" CDS 1..383 /gene="ampC" /coded_by="AJ009979.1:1..1152" /transl_table=11 /db_xref="GOA:Q9L4R5" /db_xref="HSSP:Q99QC1" /db_xref="InterPro:IPR001466" /db_xref="InterPro:IPR001586" /db_xref="InterPro:IPR012338" /db_xref="UniProtKB/TrEMBL:Q9L4R5"

Mascot: http://www.matrixscience.com/

Mascot Search Results

Protein View

Match to: **gi|57491188** Score: **127**
carbapenem-hydrolyzing oxacillinase OXA-71 [Acinetobacter baumannii]
Found in search of C:\Temp\masB0.tmp

Nominal mass (M_r): **30635**; Calculated pI value: **8.43**

NCBI BLAST search of gi|57491188 against nr
Unformatted sequence string for pasting into other applications

Taxonomy: Acinetobacter baumannii
Links to retrieve other entries containing this sequence from NCBI Entrez:
gi|58701180 from Acinetobacter baumannii

Fixed modifications: Carbamidomethyl (C)
Variable modifications: Oxidation (M)
Cleavage by Trypsin: cuts C-term side of KR unless next residue is P
Sequence Coverage: **8%**

Matched peptides shown in **Bold Red**

```
  1 MNIKALLLIT SAIFISACSP YIVTANPNHS ASKSDEKAEK IKNLFNEAHT
 51 TGVLVIQQGQ TQQSYGNDLA RASTEYVPAS TFKMLNALIG LEHHKTTTTE
101 VFKWDGQKRL FPEWEKDMTL GDAMKASAIP VYQDLARRIG LELMSKEVKR
151 VGYGNADIGT QVDNFWLVGP LKITPQQEAQ FAYKLANKTL PFSPKVQDEV
201 QSMLFIEEKN GNKIYAKSGW GWDVDPQVGW LTGWVVQPQG NIVAFSLNLE
251 MKKGIPSSVR KEITYKSLEQ LGIL
```

Show predicted peptides also

Sort Peptides By ◉ Residue Number ○ Increasing Mass ○ Decreasing Mass

Start - End	Observed	Mr(expt)	Mr(calc)	Delta	Miss	Sequence
150 - 172	840.7504	2519.2294	2518.2969	0.9325	1	K.RVGYGNADIGTQVDNFWLVGPLK.I (Ions score

41)

151 - 172 1182.5615 2363.1084 2362.1958 0.9126 0 R.VGYGNADIGTQVDNFWLVGPLK.I (Ions score 86)

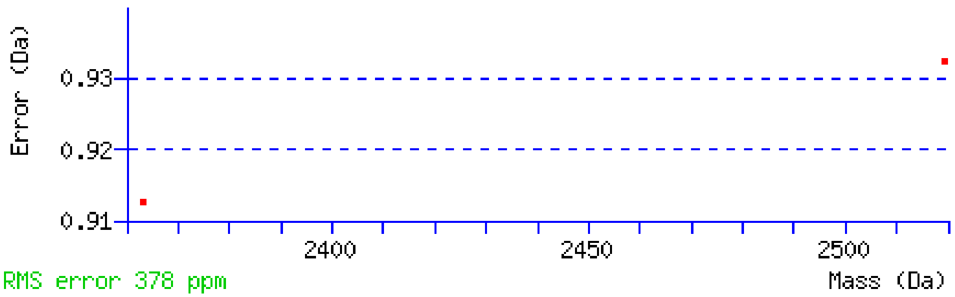

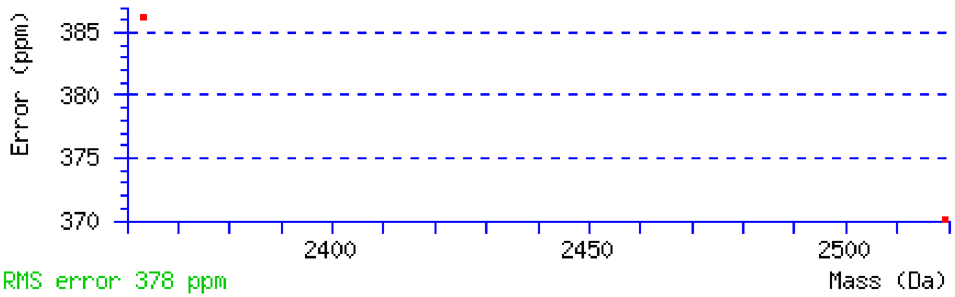

```
LOCUS       AAW51372                 274 aa            linear   BCT 29-SEP-2005
DEFINITION  carbapenem-hydrolyzing oxacillinase OXA-71 [Acinetobacter
            baumannii].
ACCESSION   AAW51372
VERSION     AAW51372.1  GI:57491188
DBSOURCE    accession AY859528.1
KEYWORDS    .
SOURCE      Acinetobacter baumannii
  ORGANISM  Acinetobacter baumannii
            Bacteria; Proteobacteria; Gammaproteobacteria; Pseudomonadales;
            Moraxellaceae; Acinetobacter; Acinetobacter calcoaceticus/baumannii
            complex.
REFERENCE   1  (residues 1 to 274)
  AUTHORS   Heritier,C., Poirel,L., Fournier,P.E., Claverie,J.M., Raoult,D. and
            Nordmann,P.
  TITLE     Characterization of the naturally occurring oxacillinase of
            Acinetobacter baumannii
  JOURNAL   Antimicrob. Agents Chemother. 49 (10), 4174-4179 (2005)
   PUBMED   16189095
REFERENCE   2  (residues 1 to 274)
  AUTHORS   Poirel,L. and Nordmann,P.
  TITLE     Direct Submission
  JOURNAL   Submitted (17-DEC-2004) Service de Bacteriologie-Virologie, Hopital
            de Bicetre, 78, rue du General Leclerc, Le Kremlin Bicetre 94275,
```

```
             France
COMMENT      Method: conceptual translation supplied by author.
FEATURES              Location/Qualifiers
     source           1..274
                      /organism="Acinetobacter baumannii"
                      /db_xref="taxon:470"
     Protein          1..274
                      /product="carbapenem-hydrolyzing oxacillinase OXA-71"
     Region           50..274
                      /region_name="Transpeptidase"
                      /note="Penicillin binding protein transpeptidase domain;
                      cl01039"
                      /db_xref="CDD:154162"
     CDS              1..274
                      /gene="bla-OXA-71"
                      /coded_by="AY859528.1:1..825"
                      /transl_table=11
```

Mascot: http://www.matrixscience.com/

Mascot Search Results

Protein View

Match to: **ATPB_ACIBC** Score: **860**
ATP synthase subunit beta OS=Acinetobacter baumannii (strain ACICU) GN=atpD PE=3 SV=1
Found in search of C:\Temp\mas2F.tmp

Nominal mass (M_r): **50300**; Calculated pI value: **5.03**

NCBI BLAST search of ATPB_ACIBC against nr
Unformatted sequence string for pasting into other applications

Taxonomy: Acinetobacter baumannii ACICU

Fixed modifications: Carbamidomethyl (C)
Variable modifications: Oxidation (M)
Cleavage by Trypsin: cuts C-term side of KR unless next residue is P
Sequence Coverage: **48%**

Matched peptides shown in **Bold Red**

```
  1 MSSGRIIQII GAVIDVEFER TSVPKIYDAL QVDGTETTLE VQQQLGDGVV
 51 RTIAMGSTEG LKRGLTVTST NAPISVPVGT ATLGRIMDVL GRPIDEAGPV
101 ATEERLPIHR QAPSYAEQAA STDLLETGIK VIDLLCPFAK GGKVGLFGGA
151 GVGKTVNMME LINNIAKAHS GLSVFAGVGE RTREGNDFYH EMKDSNVLDK
201 VAMVYGQMNE PPGNRLRVAL TGLTMAEYFR DEKDENGKGR DVLLFVDNIY
251 RYTLAGTEVS ALLGRMPSAV GYQPTLAEEM GVLQERITST KSGSITSIQA
301 VYVPADDLTD PSPATTFAHL DATVVLSRDI ASSGIYPAID PLDSTSRQLD
351 PLVVGQEHYE IARAVQNVLQ RYKELKDIIA ILGMDELAEE DKLVVYRARK
401 IQRFFSQPFH VAEVFTGAPG KLVPLKETIR GFKGLLAGEY DHIPEQAFYM
451 VGGIDEVIAK AEKL
```

Show predicted peptides also

Sort Peptides By   (•) Residue Number   ( ) Increasing Mass   ( ) Decreasing Mass

Start - End   Observed   Mr(expt)   Mr(calc)   Delta   Miss Sequence

26 - 51	950.1423	2847.4052	2846.4298	0.9753	0	K.IYDALQVDGTETTLEVQQQLGDGVVR.T	(Ions score 93)
64 - 85	704.7760	2111.3061	2111.1587	0.1475	0	R.GLTVTSTNAPISVPVGTATLGR.I	(Ions score 26)
64 - 85	1057.0977	2112.1809	2111.1587	1.0222	0	R.GLTVTSTNAPISVPVGTATLGR.I	(Ions score 27)
64 - 85	1057.1099	2112.2052	2111.1587	1.0466	0	R.GLTVTSTNAPISVPVGTATLGR.I	(Ions score 22)
86 - 105	729.0422	2184.1049	2183.0892	1.0157	0	R.IMDVLGRPIDEAGPVATEER.L	Oxidation (M) (Ions score 79)
131 - 140	588.3135	1174.6124	1174.6420	-0.0296	0	K.VIDLLCPFAK.G	(Ions score 32)
144 - 154	481.2877	960.5608	960.5393	0.0215	0	K.VGLFGGAGVGK.T	(Ions score 61)
168 - 181	693.3792	1384.7438	1385.7052	-0.9613	0	K.AHSGLSVFAGVGER.T	(Ions score 16)
168 - 181	693.8704	1385.7263	1385.7052	0.0211	0	K.AHSGLSVFAGVGER.T	(Ions score 79)
218 - 230	736.3869	1470.7593	1470.7541	0.0053	0	R.VALTGLTMAEYFR.D	(Ions score 90)
218 - 230	744.3923	1486.7700	1486.7490	0.0210	0	R.VALTGLTMAEYFR.D	Oxidation (M) (Ions score 55)
241 - 251	683.8805	1365.7465	1365.7292	0.0173	0	R.DVLLFVDNIYR.Y	(Ions score 57)
252 - 265	725.9000	1449.7855	1449.7827	0.0027	0	R.YTLAGTEVSALLGR.M	(Ions score 93)
266 - 286	780.3000	2337.8782	2337.0981	0.7801	0	R.MPSAVGYQPTLAEEMGVLQER.I	2 Oxidation (M) (Ions score 26)
266 - 286	780.3692	2338.0858	2337.0981	0.9877	0	R.MPSAVGYQPTLAEEMGVLQER.I	2 Oxidation (M) (Ions score 79)
329 - 347	989.5004	1976.9863	1976.9691	0.0172	0	R.DIASSGIYPAIDPLDSTSR.Q	(Ions score 71)
404 - 421	983.9978	1965.9810	1964.9785	1.0025	0	R.FFSQPFHVAEVFTGAPGK.L	(Ions score 76)
434 - 460	984.4859	2950.4359	2950.4422	-0.0064	0	K.GLLAGEYDHIPEQAFYMVGGIDEVIAK.A	Oxidation (M) (Ions score 21)

Error: try setting browser cache to automatic.

Error: try setting browser cache to automatic.

AC B2I102; DT 14-APR-2009, integrated into UniProtKB/Swiss-Prot. DT 10-JUN-2008, sequence version 1. DT 27-JUL-2011, entry version 26. DE RecName: Full=ATP synthase subunit beta; DE EC=3.6.3.14; DE AltName: Full=ATP synthase F1 sector subunit beta; DE AltName: Full=F-ATPase subunit beta; GN Name=atpD; OrderedLocusNames=ACICU_00178; OS Acinetobacter baumannii (strain ACICU). OC Bacteria; Proteobacteria; Gammaproteobacteria; Pseudomonadales; OC Moraxellaceae; Acinetobacter; OC Acinetobacter calcoaceticus/ baumannii complex. OX NCBI_TaxID=405416; RN [1] RP NUCLEOTIDE SEQUENCE [LARGE SCALE GENOMIC DNA]. RC STRAIN=ACICU; RX PubMed=18411315; DOI=10.1128/AAC.01643-07; RA Iacono M., Villa L., Fortini D., Bordoni R., Imperi F., Bonnal R.J., RA Sicheritz-Ponten T., De Bellis G., Visca P., Cassone A., Carattoli A.; RT "Whole-genome pyrosequencing of an epidemic multidrug-resistant RT Acinetobacter baumannii strain belonging to the European clone II RT group."; RL Antimicrob. Agents Chemother. 52:2616-2625(2008). CC -!- FUNCTION: Produces ATP from ADP in the presence of a proton CC gradient across the membrane. The catalytic sites are hosted CC primarily by the beta subunits (By similarity). CC -!- CATALYTIC ACTIVITY: ATP + H(2)O + H(+)(In) = ADP + phosphate + CC H(+)(Out). CC -!- SUBUNIT: F-type ATPases have 2 components, CF(1) - the catalytic CC core - and CF(0) - the membrane proton channel. CF(1) has five CC subunits: alpha(3), beta (3), gamma(1), delta(1), epsilon(1). CF(0) CC has three main subunits: a(1), b(2) and c(9-12). The alpha and CC beta chains form an alternating ring which encloses part of the CC gamma chain. CF(1) is attached to CF(0) by a central stalk formed CC by the gamma and epsilon chains, while a peripheral stalk is CC formed by the delta and b chains (By similarity). CC -!- SUBCELLULAR LOCATION: Cell inner membrane; Peripheral membrane CC protein (By similarity). CC -!- SIMILARITY: Belongs to the ATPase alpha/beta chains family. CC --- CC Copyrighted by the UniProt Consortium, see http://www.uniprot.org/terms CC Distributed under the Creative Commons Attribution-NoDerivs License CC --- DR EMBL; CP000863; ACC55490.1; -; Genomic_DNA. DR RefSeq; YP_001844837.1; NC_010611.1. DR ProteinModelPortal; B2I102; -. DR SMR; B2I102; 2-464. DR STRING; B2I102; -. DR GeneID; 6236208; -. DR GenomeReviews; CP000863_GR; ACICU_00178. DR KEGG; abc:ACICU_00178; -. DR HOGENOM; HBG565875; -. DR OMA; IGQEHYD; -. DR ProtClustDB; PRK12597; -. DR GO; GO:0005886; C:plasma membrane; IEA:UniProtKB-SubCell. DR GO; GO:0045261; C:proton-transporting ATP synthase complex, catalytic core F(1); IEA:UniProtKB-KW. DR GO; GO:0005524; F: ATP binding; IEA:UniProtKB-KW. DR GO; GO:0046933; F:hydrogen ion transporting ATP synthase activity, rotational mechanism; IEA:InterPro. DR GO; GO:0008553; F:hydrogen-exporting ATPase activity, phosphorylative mechanism; IEA:InterPro. DR GO; GO:0046961; F:proton-transporting ATPase activity, rotational mechanism; IEA:InterPro. DR GO; GO:0015991; P:ATP hydrolysis coupled proton transport; IEA: InterPro. DR GO; GO:0015986; P:ATP synthesis coupled proton transport; IEA:InterPro. DR HAMAP; MF_01347; ATP_synth_beta_bact; 1; -. DR InterPro; IPR020003; ATPase_a/bsu_AS. DR InterPro; IPR003593; ATPase_AAA +_core. DR InterPro; IPR005722; ATPase_F1-cplx_bsu. DR InterPro; IPR018118; ATPase_F1/A1-cplx_a/bsu_N. DR InterPro; IPR000793; ATPase_F1/V1/A1-cplx_a/bsu_C. DR InterPro; IPR004100; ATPase_F1/V1/A1-cplx_a/bsu_N. DR InterPro; IPR000194; ATPase_F1/V1/A1_a/bsu_nucl-bd. DR InterPro; IPR024034; ATPase_F1_bsu/V1_C. DR Gene3D; G3DSA:1.10.1140.10; G3DSA:1.10.1140.10; 1. DR PANTHER; PTHR15184:SF8; ATPase_F1_b; 1. DR Pfam; PF00006; ATP-synt_ab; 1. DR Pfam; PF00306; ATP-synt_ab_C; 1. DR Pfam; PF02874; ATP-synt_ab_N; 1. DR SMART; SM00382; AAA; 1. DR SUPFAM; SSF47917; ATPase_a/b_C; 1. DR SUPFAM; SSF50615; ATPase_a/b_N; 1. DR TIGRFAMs; TIGR01039; AtpD; 1. DR PROSITE; PS00152; ATPASE_ALPHA_BETA; 1. PE 3: Inferred from homology; KW ATP synthesis; ATP-binding; Cell inner membrane; Cell membrane; CF(1); KW Complete proteome; Hydrogen ion

transport; Hydrolase; Ion transport; KW Membrane; Nucleotide-binding; Transport. FT CHAIN 1 464 ATP synthase subunit beta. FT /FTId=PRO_1000143462. FT NP_BIND 148 155 ATP (By similarity). SQ SEQUENCE 464 AA; 50274 MW; 6D679361227BC1D6 CRC64; MSSGRIIQII GAVIDVEFER TSVPKIYDAL QVDGTETTLE VQQQLGDGVV RTIAMGSTEG LKRGLTVTST NAPISVPVGT ATLGRIMDVL GRPIDEAGPV ATEERLPIHR QAPSYAEQAA STDLLETGIK VIDLLCPFAK GGKVGLFGGA GVGKTVNMME LINNIAKAHS GLSVFAGVGE RTREGNDFYH EMKDSNVLDK VAMVYGQMNE PPGNRLRVAL TGLTMAEYFR DEKDENGKGR DVLLFVDNIY RYTLAGTEVS ALLGRMPSAV GYQPTLAEEM GVLQERITST KSGSITSIQA VYVPADDLTD PSPATTFAHL DATVVLSRDI ASSGIYPAID PLDSTSRQLD PLVVGQEHYE IARAVQNVLQ RYKELKDIIA ILGMDELAEE DKLVVYRARK IQRFFSQPFH VAEVFTGAPG KLVPLKETIR GFKGLLAGEY DHIPEQAFYM VGGIDEVIAK AEKL

Mascot: http://www.matrixscience.com/

Mascot Search Results

Protein View

Match to: **ATPA_ACIBC** Score: **371**
ATP synthase subunit alpha OS=Acinetobacter baumannii (strain ACICU) GN=atpA PE=3 SV=1
Found in search of C:\Temp\mas21.tmp

Nominal mass (M_r): **55534**; Calculated pI value: **5.29**

NCBI BLAST search of ATPA_ACIBC against nr
Unformatted sequence string for pasting into other applications

Taxonomy: Acinetobacter baumannii ACICU

Fixed modifications: Carbamidomethyl (C)
Variable modifications: Oxidation (M)
Cleavage by Trypsin: cuts C-term side of KR unless next residue is P
Sequence Coverage: **16%**

Matched peptides shown in **Bold Red**

```
  1 MQQLNPSEIS ALIKQRIGDL DTSATAKNEG TIVMVSDGIV RIHGLADAMY
 51 GEMIEFDGGL FGMALNLEQD SVGAVVLGNY LSLQEGQKAR CTGRVLEVPV
101 GPELLGRVVD ALGNPIDGKG PIDAKLTDAV EKVAPGVIWR QSVDQPVQTG
151 YKSVDTMIPV GRGQRELIIG DRQTGKTAMA IDAIIAQKNS GIKCVYVAIG
201 QKQSTIANVV RKLEETGAMA YTTVVAAAAA DPAAMQYLAP YSGCTMGEYF
251 RDRGEDALII YDDLSKQAVA YRQISLLLRR PPGREAYPGD VFYLHSRLLE
301 RASRVSAEYV EKFTNGAVTG KTGSLTALPI IETQAGDVSA FVPTNVISIT
351 DGQIFLETSL FNAGIRPAVN AGISVSRVGG SAQTKIIKKL SGGIRTALAQ
401 YRELAAFAQF ASDLDEATRK QLEHGQRVTE LMKQKQYAPY SIADQAVSVY
451 ASNEGYMADV EVKKIVDFDA ALIAYFRSEY APLMKQIDET GDYNKDIEAA
501 IKAGIESFKA TQTY
```

Show predicted peptides also

Sort Peptides By ◉ Residue Number ○ Increasing Mass ○ Decreasing Mass

Start - End	Observed	Mr(expt)	Mr(calc)	Delta	Miss	Sequence
1 - 14	794.4132	1586.8119	1586.8338	-0.0218	0	-.MQQLNPSEISALIK.Q Oxidation (M) (Ions score 41)
1 - 14	794.4291	1586.8436	1586.8338	0.0099	0	-.MQQLNPSEISALIK.Q Oxidation (M) (Ions score 50)
95 - 107	689.4188	1376.8230	1376.8028	0.0203	0	R.VLEVPVGPELLGR.V (Ions score 48)
95 - 107	689.4213	1376.8280	1376.8028	0.0252	0	R.VLEVPVGPELLGR.V (Ions score 33)
252 - 266	862.4392	1722.8639	1721.8472	1.0167	1	R.DRGEDALIIYDDLSK.Q (Ions score 76)
254 - 266	726.3824	1450.7502	1450.7191	0.0311	0	R.GEDALIIYDDLSK.Q (Ions score 24)
285 - 297	777.3899	1552.7652	1552.7310	0.0342	0	R.EAYPGDVFYLHSR.L (Ions score 80)
403 - 419	927.9584	1853.9022	1853.8795	0.0227	0	R.ELAAFAQFASDLDEATR.K (Ions score 59)
465 - 477	757.4214	1512.8282	1512.7977	0.0305	0	K.IVDFDAALIAYFR.S (Ions score 38)

Error: try setting browser cache to automatic.

Error: try setting browser cache to automatic.

AC B2I100; DT 14-APR-2009, integrated into UniProtKB/Swiss-Prot. DT 10-JUN-2008, sequence version 1. DT 27-JUL-2011, entry version 28. DE RecName: Full=ATP synthase subunit alpha; DE EC=3.6.3.14; DE AltName: Full=ATP synthase F1 sector subunit alpha; DE AltName: Full=F-ATPase subunit alpha; GN Name=atpA; OrderedLocusNames=ACICU_00176; OS Acinetobacter baumannii (strain ACICU). OC Bacteria; Proteobacteria; Gammaproteobacteria; Pseudomonadales; OC Moraxellaceae; Acinetobacter; OC Acinetobacter calcoaceticus/ baumannii complex. OX NCBI_TaxID=405416; RN [1] RP NUCLEOTIDE SEQUENCE [LARGE SCALE GENOMIC DNA]. RC STRAIN=ACICU; RX PubMed=18411315; DOI=10.1128/AAC.01643-07; RA Iacono M., Villa L., Fortini D., Bordoni R., Imperi F., Bonnal R.J., RA Sicheritz-Ponten T., De Bellis G., Visca P., Cassone A., Carattoli A.; RT "Whole-genome pyrosequencing of an epidemic multidrug-resistant RT Acinetobacter baumannii strain belonging to the European clone II RT group."; RL Antimicrob. Agents Chemother. 52:2616-2625(2008). CC -!- FUNCTION: Produces ATP from ADP in the presence of a proton CC gradient across the membrane. The alpha chain is a regulatory CC subunit (By similarity). CC -!- CATALYTIC ACTIVITY: ATP + H(2)O + H(+)(In) = ADP + phosphate + CC H(+)(Out). CC -!- SUBUNIT: F-type ATPases have 2 components, CF(1) - the catalytic CC core - and CF(0) - the membrane proton channel. CF(1) has five CC subunits: alpha(3), beta(3), gamma(1), delta(1), epsilon(1). CF(0) CC has three main subunits: a(1), b(2) and c(9-12). The alpha and CC beta chains form an alternating ring which encloses part of the CC gamma chain. CF(1) is attached to CF(0) by a

central stalk formed CC by the gamma and epsilon chains, while a peripheral stalk is CC formed by the delta and b chains (By similarity). CC -!- SUBCELLULAR LOCATION: Cell inner membrane; Peripheral membrane CC protein (By similarity). CC -!- SIMILARITY: Belongs to the ATPase alpha/beta chains family. CC --- CC Copyrighted by the UniProt Consortium, see http://www.uniprot.org/terms CC Distributed under the Creative Commons Attribution-NoDerivs License CC --- DR EMBL; CP000863; ACC55488.1; -; Genomic_DNA. DR RefSeq; YP_001844835.1; NC_010611.1. DR ProteinModelPortal; B2I100; -. DR SMR; B2I100; 26-511. DR STRING; B2I100; -. DR GeneID; 6236232; -. DR GenomeReviews; CP000863_GR; ACICU_00176. DR KEGG; abc:ACICU_00176; -. DR HOGENOM; HBG565875; -. DR OMA; GSDRDIK; -. DR ProtClustDB; PRK09281; -. DR GO; GO:0005886; C:plasma membrane; IEA:UniProtKB-SubCell. DR GO; GO:0045261; C:proton-transporting ATP synthase complex, catalytic core F(1); IEA:UniProtKB-KW. DR GO; GO:0005524; F:ATP binding; IEA:UniProtKB-KW. DR GO; GO:0046933; F:hydrogen ion transporting ATP synthase activity, rotational mechanism; IEA:InterPro. DR GO; GO:0046961; F:proton-transporting ATPase activity, rotational mechanism; IEA:InterPro. DR GO; GO:0015991; P:ATP hydrolysis coupled proton transport; IEA:InterPro. DR GO; GO:0015986; P:ATP synthesis coupled proton transport; IEA:InterPro. DR HAMAP; MF_01346; ATP_synth_alpha_bact; 1; -. DR InterPro; IPR020003; ATPase_a/bsu_AS. DR InterPro; IPR005294; ATPase_F1-cplx_asu. DR InterPro; IPR018118; ATPase_F1/A1-cplx_a/bsu_N. DR InterPro; IPR023366; ATPase_F1/A1-cplx_a_su_N. DR InterPro; IPR000793; ATPase_F1/V1/A1-cplx_a/bsu_C. DR InterPro; IPR004100; ATPase_F1/V1/A1-cplx_a/bsu_N. DR InterPro; IPR000194; ATPase_F1/V1/A1_a/bsu_nucl-bd. DR Gene3D; G3DSA:2.40.30.20; G3DSA:2.40.30.20; 1. DR PANTHER; PTHR15184:SF3; ATPase_F1_a; 1. DR Pfam; PF00006; ATP-synt_ab; 1. DR Pfam; PF00306; ATP-synt_ab_C; 1. DR Pfam; PF02874; ATP-synt_ab_N; 1. DR SUPFAM; SSF47917; ATPase_a/b_C; 1. DR SUPFAM; SSF50615; ATPase_a/b_N; 1. DR TIGRFAMs; TIGR00962; AtpA; 1. DR PROSITE; PS00152; ATPASE_ALPHA_BETA; 1. PE 3: Inferred from homology; KW ATP synthesis; ATP-binding; Cell inner membrane; Cell membrane; CF(1); KW Complete proteome; Hydrogen ion transport; Hydrolase; Ion transport; KW Membrane; Nucleotide-binding; Transport. FT CHAIN 1 514 ATP synthase subunit alpha. FT /FTId=PRO_1000143331. FT NP_BIND 170 177 ATP (By similarity). FT SITE 374 374 Required for activity (By similarity). SQ SEQUENCE 514 AA; 55397 MW; 63E4A57970B5A718 CRC64; MQQLNPSEIS ALIKQRIGDL DTSATAKNEG TIVMVSDGIV RIHGLADAMY GEMIEFDGGL FGMALNLEQD SVGAVVLGNY LSLQEGQKAR CTGRVLEVPV GPELLGRVVD ALGNPIDGKG PIDAKLTDAV EKVAPGVIWR QSVDQPVQTG YKSVDTMIPV GRGQRELIIG DRQTGKTAMA IDAIIAQKNS GIKCVYVAIG QKQSTIANVV RKLEETGAMA YTTVVAAAAA DPAAMQYLAP YSGCTMGEYF RDRGEDALII YDDLSKQAVA YRQISLLLRR PPGREAYPGD VFYLHSRLLE RASRVSAEYV EKFTNGAVTG KTGSLTALPI IETQAGDVSA FVPTNVISIT DGQIFLETSL FNAGIRPAVN AGISVSRVGG SAQTKIIKKL SGGIRTALAQ YRELAAFAQF ASDLDEATRK QLEHGQRVTE LMKQKQYAPY SIADQAVSVY ASNEGYMADV EVKKIVDFDA ALIAYFRSEY APLMKQIDET GDYNKDIEAA IKAGIESFKA TQTY

Mascot: http://www.matrixscience.com/

Mascot Search Results

Protein View

Match to: **ATPA_ACIBC** Score: **318**
ATP synthase subunit alpha OS=Acinetobacter baumannii (strain ACICU) GN=atpA PE=3 SV=1
Found in search of C:\Temp\mas37.tmp

Nominal mass (M_r): **55534**; Calculated pI value: **5.29**

NCBI BLAST search of ATPA_ACIBC against nr
Unformatted sequence string for pasting into other applications

Taxonomy: Acinetobacter baumannii ACICU

Fixed modifications: Carbamidomethyl (C)
Variable modifications: Oxidation (M)
Cleavage by Trypsin: cuts C-term side of KR unless next residue is P
Sequence Coverage: **14%**

Matched peptides shown in **Bold Red**

```
  1 MQQLNPSEIS ALIKQRIGDL DTSATAKNEG TIVMVSDGIV RIHGLADAMY
 51 GEMIEFDGGL FGMALNLEQD SVGAVVLGNY LSLQEGQKAR CTGRVLEVPV
101 GPELLGRVVD ALGNPIDGKG PIDAKLTDAV EKVAPGVIWR QSVDQPVQTG
151 YKSVDTMIPV GRGQRELIIG DRQTGKTAMA IDAIIAQKNS GIKCVYVAIG
201 QKQSTIANVV RKLEETGAMA YTTVVAAAAA DPAAMQYLAP YSGCTMGEYF
251 RDRGEDALII YDDLSKQAVA YRQISLLLRR PPGREAYPGD VFYLHSRLLE
301 RASRVSAEYV EKFTNGAVTG KTGSLTALPI IETQAGDVSA FVPTNVISIT
351 DGQIFLETSL FNAGIRPAVN AGISVSRVGG SAQTKIIKKL SGGIRTALAQ
401 YRELAAFAQF ASDLDEATRK QLEHGQRVTE LMKQKQYAPY SIADQAVSVY
451 ASNEGYMADV EVKKIVDFDA ALIAYFRSEY APLMKQIDET GDYNKDIEAA
501 IKAGIESFKA TQTY
```

Show predicted peptides also

Sort Peptides By ◉ Residue Number ○ Increasing Mass ○ Decreasing Mass

Start - End	Observed	Mr(expt)	Mr(calc)	Delta	Miss	Sequence
1 - 14	794.4136	1586.8127	1586.8338	-0.0211	0	-.MQQLNPSEISALIK.Q Oxidation (M) (Ions score 74)
95 - 107	689.3677	1376.7209	1376.8028	-0.0819	0	R.VLEVPVGPELLGR.V (Ions score 44)
252 - 266	862.3844	1722.7541	1721.8472	0.9070	1	R.DRGEDALIIYDDLSK.Q (Ions score 91)
403 - 419	928.4010	1854.7874	1853.8795	0.9079	0	R.ELAAFAQFASDLDEATR.K (Ions score 87)
465 - 477	757.3651	1512.7156	1512.7977	-0.0821	0	K.IVDFDAALIAYFR.S (Ions score 24)

Error: try setting browser cache to automatic.

Error: try setting browser cache to automatic.

AC B2I100; DT 14-APR-2009, integrated into UniProtKB/Swiss-Prot. DT 10-JUN-2008, sequence version 1. DT 27-JUL-2011, entry version 28. DE RecName: Full=ATP synthase subunit alpha; DE EC=3.6.3.14; DE AltName: Full=ATP synthase F1 sector subunit alpha; DE AltName: Full=F-ATPase subunit alpha; GN Name=atpA; OrderedLocusNames=ACICU_00176; OS Acinetobacter baumannii (strain ACICU). OC Bacteria; Proteobacteria; Gammaproteobacteria; Pseudomonadales; OC Moraxellaceae; Acinetobacter; OC Acinetobacter calcoaceticus/ baumannii complex. OX NCBI_TaxID=405416; RN [1] RP NUCLEOTIDE SEQUENCE [LARGE SCALE GENOMIC DNA]. RC STRAIN=ACICU; RX PubMed=18411315; DOI=10.1128/AAC.01643-07; RA Iacono M., Villa L., Fortini D., Bordoni R., Imperi F., Bonnal R.J., RA Sicheritz-Ponten T., De Bellis G., Visca P., Cassone A., Carattoli A.; RT "Whole-genome pyrosequencing of an epidemic multidrug-resistant RT Acinetobacter baumannii strain belonging to the European clone II RT group."; RL Antimicrob. Agents Chemother. 52:2616-2625(2008). CC -!- FUNCTION: Produces ATP from ADP in the presence of a proton CC gradient across the membrane. The alpha chain is a regulatory CC subunit (By similarity). CC -!- CATALYTIC ACTIVITY: ATP + H(2)O + H(+)(In) = ADP + phosphate + CC H(+)(Out). CC -!- SUBUNIT: F-type ATPases have 2 components, CF(1) - the catalytic CC core - and CF(0) - the membrane proton channel. CF(1) has five CC subunits: alpha(3), beta(3), gamma(1), delta(1), epsilon(1). CF(0) CC has three main subunits: a(1), b(2) and c(9-12). The alpha and CC beta chains form an alternating ring which encloses part of the CC gamma chain. CF(1) is attached to CF(0) by a central stalk formed CC by the gamma and epsilon chains, while a peripheral stalk is CC formed by the delta and b chains (By similarity). CC -!- SUBCELLULAR LOCATION: Cell inner membrane; Peripheral membrane CC protein (By similarity). CC -!- SIMILARITY: Belongs to the ATPase alpha/beta chains family. CC --- CC Copyrighted by the UniProt Consortium, see http://www.uniprot.org/terms CC Distributed under the Creative Commons Attribution-

NoDerivs License CC --- DR EMBL; CP000863; ACC55488.1; -; Genomic_DNA. DR RefSeq; YP_001844835.1; NC_010611.1. DR ProteinModelPortal; B2I100; -. DR SMR; B2I100; 26-511. DR STRING; B2I100; -. DR GeneID; 6236232; -. DR GenomeReviews; CP000863_GR; ACICU_00176. DR KEGG; abc:ACICU_00176; -. DR HOGENOM; HBG565875; -. DR OMA; GSDRDIK; -. DR ProtClustDB; PRK09281; -. DR GO; GO:0005886; C:plasma membrane; IEA:UniProtKB-SubCell. DR GO; GO:0045261; C:proton-transporting ATP synthase complex, catalytic core F(1); IEA:UniProtKB-KW. DR GO; GO:0005524; F:ATP binding; IEA:UniProtKB-KW. DR GO; GO:0046933; F:hydrogen ion transporting ATP synthase activity, rotational mechanism; IEA:InterPro. DR GO; GO:0046961; F:proton-transporting ATPase activity, rotational mechanism; IEA:InterPro. DR GO; GO:0015991; P:ATP hydrolysis coupled proton transport; IEA:InterPro. DR GO; GO:0015986; P:ATP synthesis coupled proton transport; IEA:InterPro. DR HAMAP; MF_01346; ATP_synth_alpha_bact; 1; -. DR InterPro; IPR020003; ATPase_a/bsu_AS. DR InterPro; IPR005294; ATPase_F1-cplx_asu. DR InterPro; IPR018118; ATPase_F1/A1-cplx_a/bsu_N. DR InterPro; IPR023366; ATPase_F1/A1-cplx_a_su_N. DR InterPro; IPR000793; ATPase_F1/V1/A1-cplx_a/bsu_C. DR InterPro; IPR004100; ATPase_F1/V1/A1-cplx_a/bsu_N. DR InterPro; IPR000194; ATPase_F1/V1/A1_a/bsu_nucl-bd. DR Gene3D; G3DSA:2.40.30.20; G3DSA:2.40.30.20; 1. DR PANTHER; PTHR15184:SF3; ATPase_F1_a; 1. DR Pfam; PF00006; ATP-synt_ab; 1. DR Pfam; PF00306; ATP-synt_ab_C; 1. DR Pfam; PF02874; ATP-synt_ab_N; 1. DR SUPFAM; SSF47917; ATPase_a/b_C; 1. DR SUPFAM; SSF50615; ATPase_a/b_N; 1. DR TIGRFAMs; TIGR00962; AtpA; 1. DR PROSITE; PS00152; ATPASE_ALPHA_BETA; 1. PE 3: Inferred from homology; KW ATP synthesis; ATP-binding; Cell inner membrane; Cell membrane; CF(1); KW Complete proteome; Hydrogen ion transport; Hydrolase; Ion transport; KW Membrane; Nucleotide-binding; Transport. FT CHAIN 1 514 ATP synthase subunit alpha. FT /FTId=PRO_1000143331. FT NP_BIND 170 177 ATP (By similarity). FT SITE 374 374 Required for activity (By similarity). SQ SEQUENCE 514 AA; 55397 MW; 63E4A57970B5A718 CRC64; MQQLNPSEIS ALIKQRIGDL DTSATAKNEG TIVMVSDGIV RIHGLADAMY GEMIEFDGGL FGMALNLEQD SVGAVVLGNY LSLQEGQKAR CTGRVLEVPV GPELLGRVVD ALGNPIDGKG PIDAKLTDAV EKVAPGVIWR QSVDQPVQTG YKSVDTMIPV GRGQRELIIG DRQTGKTAMA IDAIIAQKNS GIKCVYVAIG QKQSTIANVV RKLEETGAMA YTTVVAAAAA DPAAMQYLAP YSGCTMGEYF RDRGEDALII YDDLSKQAVA YRQISLLLRR PPGREAYPGD VFYLHSRLLE RASRVSAEYV EKFTNGAVTG KTGSLTALPI IETQAGDVSA FVPTNVISIT DGQIFLETSL FNAGIRPAVN AGISVSRVGG SAQTKIIKKL SGGIRTALAQ YRELAAFAQF ASDLDEATRK QLEHGQRVTE LMKQKQYAPY SIADQAVSVY ASNEGYMADV EVKKIVDFDA ALIAYFRSEY APLMKQIDET GDYNKDIEAA IKAGIESFKA TQTY

Mascot: http://www.matrixscience.com/

Mascot Search Results

Protein View

Match to: **gi|126642748** Score: **63**
dihydrolipoamide dehydrogenase [Acinetobacter baumannii ATCC 17978]
Found in search of C:\Temp\mas41.tmp

Nominal mass (M_r): **42487**; Calculated pI value: **5.64**

NCBI BLAST search of gi|126642748 against nr

Unformatted sequence string for pasting into other applications

Taxonomy: Acinetobacter baumannii ATCC 17978

Fixed modifications: Carbamidomethyl (C)
Variable modifications: Oxidation (M)
Cleavage by Trypsin: cuts C-term side of KR unless next residue is P
Sequence Coverage: **7%**

Matched peptides shown in **Bold Red**

```
  1 MHHLADHGIT TGEVNFDLAK LLARKDKIVD QLTGGIDQLL KGNGIEWLKG
 51 TGKLLAGKKV EFVPHEGETQ ILEPKYVILA SGSVPVNIPV APVDQDIIVD
101 STGALNFPEV PKRLGVIGAG VIGLELGSVW RRLGAEVVVF EAMDAFLPMA
151 DKALSKEYQK ILTKQGLDIR IGAKVSGTEV NGREVTVKYT QAGEDKEQTF
201 DKLIVCVGRK AYAEGLLAED SGIKLTERGL VEVNDHCATS VEGVYAIGDL
251 VRGPMLAHKA MEEGVMAVER IHGHAAQVNY DTIISVIYTH PEAAWVGLTE
301 EQAKEKGHEV KTGQFGFAVN GRALAAGEGA GFVKFVADAK TDRLLGMHVI
351 GPAASDIVHQ GMIALEFVSS VEDLQLMTFG TQHSLKLFMK LHLL
```

Show predicted peptides also

Sort Peptides By ◉ Residue Number ○ Increasing Mass ○ Decreasing Mass

Start - End	Observed	Mr(expt)	Mr(calc)	Delta	Miss	Sequence
28 - 41	756.9571	1511.8997	1511.8559	0.0438	0	K.IVDQLTGGIDQLLK.G (Ions score 18)

59 - 75 661.0493 1980.1261 1979.0364 1.0897 1 K.KVEFVPHEGETQILEPK.Y (Ions score 46)

Error: try setting browser cache to automatic.

Error: try setting browser cache to automatic.

LOCUS YP_001085732 394 aa linear BCT 29-MAY-2010 DEFINITION dihydrolipoamide dehydrogenase [Acinetobacter baumannii ATCC 17978]. ACCESSION YP_001085732 VERSION YP_001085732.1 GI:126642748 DBLINK Project: 58731 DBSOURCE REFSEQ: accession NC_009085.1 KEYWORDS . SOURCE Acinetobacter baumannii ATCC 17978 ORGANISM Acinetobacter baumannii ATCC 17978 Bacteria; Proteobacteria; Gammaproteobacteria; Pseudomonadales; Moraxellaceae; Acinetobacter; Acinetobacter calcoaceticus/baumannii complex. REFERENCE 1 (residues 1 to 394) AUTHORS Smith,M.G., Gianoulis,T.A., Pukatzki,S., Mekalanos,J.J., Ornston,L.N., Gerstein,M. and Snyder, M. TITLE New insights into Acinetobacter baumannii pathogenesis revealed by high-density pyrosequencing and transposon mutagenesis JOURNAL Genes Dev. 21 (5), 601-614 (2007) PUBMED 17344419 REFERENCE 2 (residues 1 to 394) CONSRTM NCBI Genome Project TITLE Direct Submission JOURNAL Submitted (05-MAR-2007) National Center for Biotechnology Information, NIH, Bethesda, MD 20894, USA REFERENCE 3 (residues 1 to 394) AUTHORS Smith,M.G., Gianoulis,T.A., Pukatzki,S., Mekalanos,J.J., Ornston,L.N., Gerstein,M. and Snyder,M. TITLE Direct Submission JOURNAL Submitted (27-NOV-2006) Department of Molecular, Cellular, and Developmental Biology, Yale University, 266 Whitney Ave, New Haven, CT 06520, USA COMMENT PROVISIONAL REFSEQ: This record has not yet been subject to final NCBI review. The reference sequence was derived from ABO13130. Source DNA and bacteria available from Michael Snyder (michael.snyder@yale.edu). Method: conceptual translation. FEATURES Location/Qualifiers source 1..394 /organism="Acinetobacter baumannii ATCC 17978" /strain="ATCC 17978" /db_xref="ATCC:17978" /db_xref="taxon:400667" Protein 1..394 /product="dihydrolipoamide dehydrogenase" /calculated_mol_wt=42269 Region 2..380 /region_name="PRK06467" /note="dihydrolipoamide dehydrogenase; Reviewed" /db_xref="CDD:180579" Region 113..194 /region_name="Pyr_redox" /note="Pyridine nucleotide-disulphide oxidoreductase; cl14644" /db_xref="CDD:197445" Region 285..379 /region_name="Pyr_redox_dim" /note="Pyridine nucleotide-disulphide oxidoreductase, dimerisation domain; pfam02852" /db_xref="CDD:190451" CDS 1..394 /locus_tag="A1S_2717" /coded_by="NC_009085.1:3153037..3154221" /note="E3 component of 2-oxoglutarate dehydrogenase complexGlycine oxidation system L-factor" /transl_table=11 /db_xref="GeneID:4919320"

Mascot: http://www.matrixscience.com/

Mascot Search Results

Protein View

Match to: **MDH_ACIBC** Score: **316**
Malate dehydrogenase OS=Acinetobacter baumannii (strain ACICU) GN=mdh PE=3 SV=1
Found in search of C:\Temp\mas93.tmp

Nominal mass (M_r): **35358**; Calculated pI value: **5.20**

NCBI BLAST search of MDH_ACIBC against nr
Unformatted sequence string for pasting into other applications

Taxonomy: Acinetobacter baumannii ACICU

Fixed modifications: Carbamidomethyl (C)
Variable modifications: Oxidation (M)
Cleavage by Trypsin: cuts C-term side of KR unless next residue is P
Sequence Coverage: **21%**

Matched peptides shown in **Bold Red**

```
  1 MKQPVRVAVT GAAGQIGYSL LFRIASGEML GKDQPVILQL LEVPVEKAQQ
 51 ALKGVMMELD DCAFPLLAGM IGTDDPKVAF KDADYALLVG SRPRGPGMER
101 ADLLKVNGEI FIGQGQALNE VASRDVKVLV VGNPANTNAY IAMKSAPDLP
151 AKNFTAMLRL DHNRALTQVA QKAGVAVADI EKLTVWGNHS PTMYADYRFA
201 TANGESLKDK INDPAWNKDV FLPTVGKRGA AIIEARGLSS AASAANAAID
251 HMRDWALGTN GKWVTMGVPS DGSYGIPEGV MFGFPVTTEN GEYKIVQGLE
301 IDEFSRERIN FTLNELEEER AAIADMVK
```

Show predicted peptides also

Sort Peptides By ◉ Residue Number ○ Increasing Mass ○ Decreasing Mass

Start - End	Observed	Mr(expt)	Mr(calc)	Delta	Miss	Sequence
33 - 47	860.4943	1718.9741	1718.9818	-0.0078	0	K.DQPVILQLLEVPVEK.A (Ions score 44)
82 - 94	716.8854	1431.7562	1431.7470	0.0092	0	K.DADYALLVGSRPR.G (Ions score 61)

237 - 253	829.9066	1657.7986	1657.7842	0.0145	0	R.GLSSAASAANAAIDHMR.D	Oxidation (M) (Ions score 59)
295 - 306	703.3758	1404.7371	1404.7249	0.0122	0	K.IVQGLEIDEFSR.E	(Ions score 73)
309 - 320	753.8813	1505.7481	1505.7361	0.0120	0	R.INFTLNELEEER.A	(Ions score 80)

Error: try setting browser cache to automatic.

Error: try setting browser cache to automatic.

AC B2HZ52; DT 14-APR-2009, integrated into UniProtKB/Swiss-Prot. DT 10-JUN-2008, sequence version 1. DT 27-JUL-2011, entry version 27. DE RecName: Full=Malate dehydrogenase; DE EC=1.1.1.37; GN Name=mdh; OrderedLocusNames=ACICU_03222; OS Acinetobacter baumannii (strain ACICU). OC Bacteria; Proteobacteria; Gammaproteobacteria; Pseudomonadales; OC Moraxellaceae; Acinetobacter; OC Acinetobacter calcoaceticus/baumannii complex. OX NCBI_TaxID=405416; RN [1] RP NUCLEOTIDE SEQUENCE [LARGE SCALE GENOMIC DNA]. RC STRAIN=ACICU; RX PubMed=18411315; DOI=10.1128/AAC.01643-07; RA Iacono M., Villa L., Fortini D., Bordoni R., Imperi F., Bonnal R.J., RA Sicheritz-Ponten T., De Bellis G., Visca P., Cassone A., Carattoli A.; RT "Whole-genome pyrosequencing of an epidemic multidrug-resistant RT Acinetobacter baumannii strain belonging to the European clone II RT group."; RL Antimicrob. Agents Chemother. 52:2616-2625(2008). CC -!- FUNCTION: Catalyzes the reversible oxidation of malate to CC oxaloacetate (By similarity). CC -!- CATALYTIC ACTIVITY: (S)-malate + NAD(+) = oxaloacetate + NADH. CC -!- SUBUNIT: Homodimer (By similarity). CC -!- SIMILARITY: Belongs to the LDH/MDH superfamily. MDH type 2 family. CC --- CC Copyrighted by the UniProt Consortium, see http://www.uniprot.org/terms CC Distributed under the Creative Commons Attribution-NoDerivs License CC --- DR EMBL; CP000863; ACC58534.1; -; Genomic_DNA. DR RefSeq; YP_001847881.1; NC_010611.1. DR ProteinModelPortal; B2HZ52; -. DR SMR; B2HZ52; 2-327. DR STRING; B2HZ52; -. DR GeneID; 6234431; -. DR GenomeReviews; CP000863_GR; ACICU_03222. DR KEGG; abc:ACICU_03222; -. DR HOGENOM; HBG289884; -. DR OMA; MIIWGNH; -. DR ProtClustDB; PRK05442; -. DR GO; GO:0005488; F:binding; IEA:InterPro. DR GO; GO:0030060; F:L-malate dehydrogenase activity; IEA:EC. DR GO; GO:0044262; P:cellular carbohydrate metabolic process; IEA:InterPro. DR GO; GO:0006108; P:malate metabolic process; IEA:InterPro. DR GO; GO:0006099; P:tricarboxylic acid cycle; IEA:UniProtKB-KW. DR HAMAP; MF_01517; Malate_dehydrog_2; 1; -. DR InterPro; IPR001557; L-lactate/malate_DH. DR InterPro; IPR022383; Lactate/malate_DH_C. DR InterPro; IPR001236; Lactate/malate_DH_N. DR InterPro; IPR015955; Lactate_DH/Glyco_Ohase_4_C. DR InterPro; IPR010945; Malate_DH_type2. DR InterPro; IPR016040; NAD(P)-bd_dom. DR Gene3D; G3DSA:3.90.110.10; lact_mal_DH; 1. DR Gene3D;

G3DSA:3.40.50.720; NAD(P)-bd; 1. DR PANTHER; PTHR23382; MDH_SF1; 1. DR Pfam; PF02866; Ldh_1_C; 1. DR Pfam; PF00056; Ldh_1_N; 1. DR PIRSF; PIRSF000102; Lac_mal_DH; 1. DR SUPFAM; SSF56327; Lactate_DH/ Glyco_hydro_4_C; 1. DR TIGRFAMs; TIGR01759; MalateDH-SF1; 1. DR PROSITE; PS00068; MDH; FALSE_NEG. PE 3: Inferred from homology; KW Complete proteome; NAD; Oxidoreductase; Tricarboxylic acid cycle. FT CHAIN 1 328 Malate dehydrogenase. FT /FTId=PRO_1000191605. FT NP_BIND 11 17 NAD (By similarity). FT NP_BIND 131 133 NAD (By similarity). FT ACT_SITE 189 189 Proton acceptor (By similarity). FT BINDING 94 94 Substrate (By similarity). FT BINDING 100 100 Substrate (By similarity). FT BINDING 107 107 NAD (By similarity). FT BINDING 114 114 NAD (By similarity). FT BINDING 133 133 Substrate (By similarity). FT BINDING 164 164 Substrate (By similarity). SQ SEQUENCE 328 AA; 35323 MW; 4A3ABDD04F615F6E CRC64; MKQPVRVAVT GAAGQIGYSL LFRIASGEML GKDQPVILQL LEVPVEKAQQ ALKGVMMELD DCAFPLLAGM IGTDDPKVAF KDADYALLVG SRPRGPGMER ADLLKVNGEI FIGQGQALNE VASRDVKVLV VGNPANTNAY IAMKSAPDLP AKNFTAMLRL DHNRALTQVA QKAGVAVADI EKLTVWGNHS PTMYADYRFA TANGESLKDK INDPAWNKDV FLPTVGKRGA AIIEARGLSS AASAANAAID HMRDWALGTN GKWVTMGVPS DGSYGIPEGV MFGFPVTTEN GEYKIVQGLE IDEFSRERIN FTLNELEEER AAIADMVK

Mascot: http://www.matrixscience.com/

Mascot Search Results

Protein View

Match to: **gi|260549007** Score: **276**
pyruvate/2-oxoglutarate dehydrogenase complex [Acinetobacter sp. RUH2624]
Found in search of C:\Temp\mas35.tmp

Nominal mass (M_r): **51197**; Calculated pI value: **5.96**

NCBI BLAST search of gi|260549007 against nr
Unformatted sequence string for pasting into other applications

Taxonomy: Acinetobacter sp. RUH2624
Links to retrieve other entries containing this sequence from NCBI Entrez:
gi|260408175 from Acinetobacter sp. RUH2624

Fixed modifications: Carbamidomethyl (C)
Variable modifications: Oxidation (M)
Cleavage by Trypsin: cuts C-term side of KR unless next residue is P
Sequence Coverage: **15%**

Matched peptides shown in **Bold Red**

```
  1 MSQQFDLVVI GGGPGGYEAA IRAAQLGFKV ACIEKRIHNG KPSLGGTCLN
 51 VGCIPSKALL DSSHRYEDTV HHLADHGITT GEVNFDLAKL LARKDKIVDQ
101 LTGGIDQLLK GNGIEWLKGT GKLLAGKKVE FVSHEGETQV LEPKYVILAS
151 GSVPVNIPVA PVDQDIIVDS TGALNFPEVP KRLGVIGAGV IGLELGSVWR
201 RLGAEVVVFE AMDAFLPMAD KALAKEYQKL LTKQGLDIRV GAKVSGTEVN
251 GREVTVKYTQ GGEEKTQTFD KLIVCVGRKA YAEGLLAEDS GIKLTERGLV
301 EVNDHCATSV EGVYAIGDLV RGPMLAHKAM EEGVMAVERI HGHAAQVNYD
351 TIISVIYTHP EAAWVGLTEE QAKEKGHEVK TGQFGFAVNG RALAAGEGAG
401 FVKFVADAKT DRLLGMHVIG PAASDIVHQG MIALEFVSSV EDLQLMTFGH
451 PTFSEVVHEA ALAVDGRAIH AIQRKRK
```

Show predicted peptides also

Sort Peptides By ◉ Residue Number ○ Increasing Mass ○ Decreasing Mass

Start - End	Observed	Mr(expt)	Mr(calc)	Delta	Miss	Sequence
2 - 22	1068.0567	2134.0988	2133.0855	1.0133	0	M.SQQFDLVVIGGGPGGYEAAIR.A (Ions score 59)
66 - 89	895.0897	2682.2471	2681.2722	0.9750	0	R.YEDTVHHLADHGITTGEVNFDLAK.L (Ions score 57)
95 - 110	878.4802	1754.9459	1754.9778	-0.0319	1	K.DKIVDQLTGGIDQLLK.G (Ions score 57)
97 - 110	757.4523	1512.8901	1511.8559	1.0342	0	K.IVDQLTGGIDQLLK.G (Ions score 33)
279 - 293	782.4311	1562.8477	1563.8144	-0.9667	1	R.KAYAEGLLAEDSGIK.L (Ions score 22)
280 - 293	718.9245	1435.8345	1435.7194	0.1151	0	K.AYAEGLLAEDSGIK.L (Ions score 49)

Error: try setting browser cache to automatic.

Error: try setting browser cache to automatic.

LOCUS ZP_05823229 477 aa linear BCT 09-JUN-2010 DEFINITION pyruvate/2-oxoglutarate dehydrogenase complex [Acinetobacter sp. RUH2624]. ACCESSION ZP_05823229 VERSION ZP_05823229.1 GI:260549007 DBLINK Project: 40849 DBSOURCE REFSEQ: accession NZ_GG704495.1 KEYWORDS . SOURCE Acinetobacter sp. RUH2624 ORGANISM Acinetobacter sp. RUH2624 Bacteria; Proteobacteria; Gammaproteobacteria; Pseudomonadales; Moraxellaceae; Acinetobacter; Acinetobacter calcoaceticus/baumannii complex. REFERENCE 1 (residues 1 to 477) AUTHORS Ward, D., Feldgarden,M., Earl,A., Young,S.K., Zeng,Q., Koehrsen,M., Alvarado,L., Berlin,A., Bochicchio,J., Borenstein,D., Chapman,S., Chen,Z., Engels,R., Freedman,E., Gellesch,M., Goldberg,J., Griggs,A., Gujja,S., Heilman,E., Heiman,D., Hepburn,T., Howarth,C., Jen,D., Larson,L., Lewis,B., Mehta,T., Park,D., Pearson,M., Roberts,A., Saif,S., Shea,T., Shenoy,N., Sisk,P., Stolte,C., Sykes,S., Thomson,T., Walk,T., White,J., Yandava,C., Dijkshoorn,L., Haas,B., Nusbaum,C. and Birren,B. CONSRTM The Broad Institute Genome Sequencing Platform TITLE The Genome Sequence of Acinetobacter sp. strain RUH2624 JOURNAL Unpublished REFERENCE 2 (residues 1 to 477) AUTHORS Ward,D., Feldgarden,M., Earl,A., Young,S.K., Zeng,Q., Koehrsen,M., Alvarado, L., Berlin,A., Bochicchio,J., Borenstein,D., Chapman,S., Chen,Z., Engels,R., Freedman,E., Gellesch,M., Goldberg,J., Griggs,A., Gujja,S., Heilman,E., Heiman,D., Hepburn,T., Howarth,C., Jen,D., Larson,L., Lewis, B., Mehta,T., Park,D., Pearson,M., Roberts,A., Saif,S., Shea,T., Shenoy,N., Sisk,P., Stolte,C., Sykes,S., Thomson,T., Walk,T., White,J., Yandava,C., Dijkshoorn,L., Haas,B., Nusbaum,C. and Birren,B. CONSRTM The Broad Institute Genome Sequencing Platform TITLE Direct Submission JOURNAL Submitted (19-AUG-2009) Broad Institute of MIT and Harvard, 7 Cambridge Center, Cambridge, MA 02142, USA COMMENT PREDICTED REFSEQ: This

record has not been reviewed and the function is unknown. The reference sequence was derived from EEX01646. Method: conceptual translation. FEATURES Location/Qualifiers source 1..477 /organism="Acinetobacter sp. RUH2624" /strain="RUH2624" /db_xref="taxon:575564" Protein 1..477 /product="pyruvate/2-oxoglutarate dehydrogenase complex" /calculated_mol_wt=50813 Region 1..477 /region_name="PRK06467" /note="dihydrolipoamide dehydrogenase; Reviewed" /db_xref="CDD:180579" Region 5..>154 /region_name="Pyr_redox" /note="Pyridine nucleotide-disulphide oxidoreductase; cl14644" /db_xref="CDD:197445" Region 182..263 /region_name="Pyr_redox" /note="Pyridine nucleotide-disulphide oxidoreductase; cl14644" /db_xref="CDD:197445" Region 354..461 /region_name="Pyr_redox_dim" /note="Pyridine nucleotide-disulphide oxidoreductase, dimerisation domain; pfam02852" /db_xref="CDD:190451" CDS 1..477 /locus_tag="HMPREF0014_00243" /coded_by="NZ_GG704495.1:262130..263563" /transl_table=11

Mascot: http://www.matrixscience.com/

Mascot Search Results

Protein View

Match to: **gi|169632322** Score: **90**
coproporphyrinogen III oxidase [Acinetobacter baumannii SDF]
Found in search of C:\Temp\mas82.tmp

Nominal mass (M_r): **38638**; Calculated pI value: **5.27**

NCBI BLAST search of gi|169632322 against nr
Unformatted sequence string for pasting into other applications

Taxonomy: Acinetobacter baumannii SDF
Links to retrieve other entries containing this sequence from NCBI Entrez:
gi|169151114 from Acinetobacter baumannii

Fixed modifications: Carbamidomethyl (C)
Variable modifications: Oxidation (M)
Cleavage by Trypsin: cuts C-term side of KR unless next residue is P
Sequence Coverage: **6%**

Matched peptides shown in **Bold Red**

```
  1 MDLCFDVSNL FRSSLRVIMQ HPTSTDIQRV REFLLDLQAR ICAGLEQQEK
 51 AGGGTAEFII DDWERPEGGG GRSRVLQNGT VIEKGGVMFS HINISKLPPS
101 ATERHPQIAG AKAQALGVSL VIHPKNPNIP TSHANVRLFV AEREGQDPIW
151 WFGGGFDLTP FYPDDQDVLN WHQAAYDLCK PFGDNVYAEH KKWCDDYFYL
201 KHGDEQRGVG GLFFDDLNCW DFETCFKYIQ AVGNGYLNAI LPIFEKHREQ
251 PYTEAQREFQ LYRRGRYVEY NLVYDRGTLF GLQTGGRIES ILVSLPNLAA
301 WSYRPEWDED SPEKRLTDYY LKPRDWLGLE EKVA
```

Show predicted peptides also

Sort Peptides By ◉ Residue Number ○ Increasing Mass ○ Decreasing Mass

Start - End Observed Mr(expt) Mr(calc) Delta Miss Sequence

85 - 96	653.3461	1304.6777	1304.6547	0.0230	0	K.GGVMFSHINISK.L	Oxidation (M) (Ions score 50)
277 - 287	553.8227	1105.6308	1105.5880	0.0428	0	R.GTLFGLQTGGR.I	(Ions score 40)

Error: try setting browser cache to automatic.

Error: try setting browser cache to automatic.

LOCUS YP_001706058 334 aa linear BCT 17-MAY-2011 DEFINITION coproporphyrinogen III oxidase [Acinetobacter baumannii SDF]. ACCESSION YP_001706058 VERSION YP_001706058.1 GI:169632322 DBLINK Project: 61601 DBSOURCE REFSEQ: accession NC_010400.1 KEYWORDS . SOURCE Acinetobacter baumannii SDF ORGANISM Acinetobacter baumannii SDF Bacteria; Proteobacteria; Gammaproteobacteria; Pseudomonadales; Moraxellaceae; Acinetobacter; Acinetobacter calcoaceticus/baumannii complex. REFERENCE 1 (residues 1 to 334) AUTHORS Vallenet,D., Nordmann,P., Barbe,V., Poirel,L., Mangenot,S., Bataille,E., Dossat,C., Gas,S., Kreimeyer,A., Lenoble,P., Oztas,S., Poulain,J., Segurens,B., Robert,C., Abergel,C., Claverie,J.M., Raoult,D., Medigue, C., Weissenbach,J. and Cruveiller,S. TITLE Comparative analysis of Acinetobacters: three genomes for three lifestyles JOURNAL PLoS ONE 3 (3), E1805 (2008) PUBMED 18350144 REMARK Publication Status: Online-Only REFERENCE 2 (residues 1 to 334) CONSRTM NCBI Genome Project TITLE Direct Submission JOURNAL Submitted (04-MAR-2008) National Center for Biotechnology Information, NIH, Bethesda, MD 20894, USA REFERENCE 3 (residues 1 to 334) AUTHORS Genoscope -,C.E.A. TITLE Direct Submission JOURNAL Submitted (25-FEB-2008) Genoscope - Centre National de Sequencage : BP 191 91006 EVRY cedex - FRANCE (E-mail : seqref@genoscope. cns.fr - Web : www.genoscope.cns.fr) COMMENT PROVISIONAL REFSEQ: This record has not yet been subject to final NCBI review. The reference sequence was derived from CAO99780. Annotation data relative to BLAST similarities, COG assignations, enzymatic function prediction (PRIAM software), TMHMM and SignalP predictions, and synteny results (Syntonizer software) are available in BaumannoScope database via the MaGe annotation system http://www.genoscope.cns.fr/agc/mage/baumannoscope. Method: conceptual translation. FEATURES Location/Qualifiers source 1..334 /organism="Acinetobacter baumannii SDF" /strain="SDF" /db_xref="taxon:509170" Protein 1..334 /product="coproporphyrinogen III oxidase" /EC_number="1.3.3.3" /function="1.5.3.12 : heme, porphyrine" /calculated_mol_wt=38189 Region 28..327 /region_name="Coprogen_oxidas" /note="Coproporphyrinogen III oxidase; cl00433" /db_xref="CDD:193817" CDS 1..334 /gene="hemF" /locus_tag="ABSDF0387" /coded_by="complement(NC_010400.1:365545..366549)" /inference="ab initio prediction:AMIGene:2.0" /note="catalyzes the conversion of the propionic acid groups of rings I and III to vinyl groups during heme synthesis" /transl_table=11 /db_xref="GeneID:5986562"

Mascot: http://www.matrixscience.com/

{MATRIX SCIENCE} Mascot Search Results

Protein View

```
Match to: RL10_ACIB3 Score: 151
50S ribosomal protein L10 OS=Acinetobacter baumannii (strain AB307-0294) GN=rplJ PE=3 SV=1
Found in search of C:\DOCUME~1\ADMINI~1\LOCALS~1\Temp\mas5.tmp

Nominal mass (Mr): 18079; Calculated pI value: 5.62
NCBI BLAST search of RL10_ACIB3 against nr
Unformatted sequence string for pasting into other applications

Taxonomy: Acinetobacter baumannii AB307-0294

Fixed modifications: Carbamidomethyl (C)
Variable modifications: Oxidation (M)
Cleavage by Trypsin: cuts C-term side of KR unless next residue is P
Sequence Coverage: 16%

Matched peptides shown in Bold Red

    1 MALLIEDKKQ IVAEVSEVAS KAFAAVVADY QGLSVEQLTT LRVEARKLGV
   51 TTRIVRNTLA KRAFQGTQFD ILNDNLVGPT ILGFSTSEDD MGAAARLFEE
  101 FAKTNKAFEL KAAAFDGKVY QGADVSVIAN LPNQEKALTM LASVLQAPIS
  151 KLGRLITALK EKNESEAA
```

Show predicted peptides also

Sort Peptides By ◉ Residue Number ○ Increasing Mass ○ Decreasing Mass

Start - End	Observed	Mr(expt)	Mr(calc)	Delta	Miss	Sequence
9 - 21	694.4112	1386.8079	1386.7718	0.0360	1	K.KQIVAEVSEVASK.A (Ions score 81)
137 - 151	772.4795	1542.9445	1541.8851	1.0595	0	K.ALTMLASVLQAPISK.L (Ions score 70)

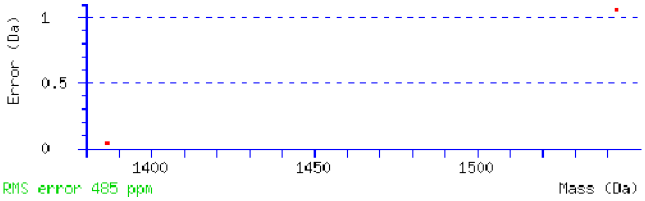

Error Distribution (ppm)

```
ID   RL10_ACIB3              Reviewed;         168 AA.
AC   B7H1K0;
DT   14-APR-2009, integrated into UniProtKB/Swiss-Prot.
DT   10-FEB-2009, sequence version 1.
DT   02-MAR-2010, entry version 10.
DE   RecName: Full=50S ribosomal protein L10;
GN   Name=rplJ; OrderedLocusNames=ABBFA_003250;
OS   Acinetobacter baumannii (strain AB307-0294).
OC   Bacteria; Proteobacteria; Gammaproteobacteria; Pseudomonadales;
OC   Moraxellaceae; Acinetobacter.
OX   NCBI_TaxID=557600;
RN   [1]
RP   NUCLEOTIDE SEQUENCE [LARGE SCALE GENOMIC DNA].
RX   PubMed=18931120; DOI=10.1128/JB.00834-08;
RA   Adams M.D., Goglin K., Molyneaux N., Hujer K.M., Lavender H.,
RA   Jamison J.J., MacDonald I.J., Martin K.M., Russo T., Campagnari A.A.,
RA   Hujer A.M., Bonomo R.A., Gill S.R.;
RT   "Comparative genome sequence analysis of multidrug-resistant
RT   Acinetobacter baumannii.";
RL   J. Bacteriol. 190:8053-8064(2008).
CC   -!- SIMILARITY: Belongs to the ribosomal protein L10P family.
CC   -----------------------------------------------------------------------
CC   Copyrighted by the UniProt Consortium, see http://www.uniprot.org/terms
CC   Distributed under the Creative Commons Attribution-NoDerivs License
CC   -----------------------------------------------------------------------
DR   EMBL; CP001172; ACJ56249.1; -; Genomic_DNA.
DR   RefSeq; YP_002327131.1; -.
DR   SMR; B7H1K0; 1-158.
DR   GeneID; 7059301; -.
DR   GenomeReviews; CP001172_GR; ABBFA_003250.
DR   KEGG; abb:ABBFA_003250; -.
DR   HOGENOM; HBG597359; -.
```

```
DR   OMA; AKANDKF; -.
DR   ProtClustDB; PRK00099; -.
DR   GO; GO:0005840; C:ribosome; IEA:UniProtKB-K .
DR   GO; GO:0003735; F:structural constituent of ribosome; IEA:InterPro.
DR   GO; GO:0042254; P:ribosome biogenesis; IEA:InterPro.
DR   GO; GO:0006412; P:translation; IEA:HAMAP.
DR   HAMAP; MF_00362; Ribosomal_L10; 1; -.
DR   InterPro; IPR001790; Ribosomal_L10.
DR   InterPro; IPR002363; Ribosomal_L10_eubac_CS.
DR   Pfam; PF00466; Ribosomal_L10; 1.
DR   PROSITE; PS01109; RIBOSOMAL_L10; 1.
PE   3: Inferred from homology;
K    Complete proteome; Ribonucleoprotein; Ribosomal protein.
FT   CHAIN         1    168       50S ribosomal protein L10.
FT                                /FTId=PRO_1000120899.
SQ   SEQUENCE   168 AA;  18090 M ;  C4B9A576F0BA5EA3 CRC64;
     MALLIEDKKQ IVAEVSEVAS KAFAAVVADY QGLSVEQLTT LRVEARKLGV TTRIVRNTLA
     KRAFQGTQFD ILNDNLVGPT ILGFSTSEDD MGAAARLFEE FAKTNKAFEL KAAAFDGKVY
     QGADVSVIAN LPNQEKALTM LASVLQAPIS KLGRLITALK EKNESEAA
```

Mascot http://www.matri science.com/

Mascot Search Results

Protein View

Match to: **RS1_ECO57** Score: **117**
30S ribosomal protein S1 OS=Escherichia coli O157:H7 GN=rpsA PE=3 SV=1
Found in search of C:\Temp\mas32.tmp

Nominal mass (M_r): **61235**; Calculated pI value: **4.89**

NCBI BLAST search of RS1_ECO57 against nr
Unformatted sequence string for pasting into other applications

Taxonomy: Escherichia coli O157:H7

Fixed modifications: Carbamidomethyl (C)
Variable modifications: Oxidation (M)
Cleavage by Trypsin: cuts C-term side of KR unless next residue is P
Sequence Coverage: **2%**

Matched peptides shown in **Bold Red**

```
  1 MTESFAQLFE ESLKEIETRP GSIVRGVVVA IDKDVVLVDA GLKSESAIPA
 51 EQFKNAQGEL EIQVGDEVDV ALDAVEDGFG ETLLSREKAK RHEAWITLEK
101 AYEDAETVTG VINGKVKGGF TVELNGIRAF LPGSLVDVRP VRDTLHLEGK
151 ELEFKVIKLD QKRNNVVVSR RAVIESENSA ERDQLLENLQ EGMEVKGIVK
201 NLTDYGAFVD LGGVDGLLHI TDMAWKRVKH PSEIVNVGDE ITVKVLKFDR
251 ERTRVSLGLK QLGEDPWVAI AKRYPEGTKL TGRVTNLTDY GCFVEIEEGV
301 EGLVHVSEMD WTNKNIHPSK VVNVGDVVEV MVLDIDEERR RISLGLKQCK
351 ANPWQQFAET HNKGDRVEGK IKSITDFGIF IGLDGGIDGL VHLSDISWNV
401 AGEEAVREYK KGDEIAAVVL QVDAERERIS LGVKQLAEDP FNNWVALNKK
451 GAIVTGKVTA VDAKGATVEL ADGVEGYLRA SEASRDRVED ATLVLSVGDE
501 VEAKFTGVDR KNRAISLSVR AKDEADEKDA IATVNKQEDA NFSNNAMAEA
551 FKAAKGE
```

Show predicted peptides also

Sort Peptides By ◉ Residue Number ○ Increasing Mass ○ Decreasing Mass

Start - End	Observed	Mr(expt)	Mr(calc)	Delta	Miss	Sequence
1 - 14	838.3633	1674.7120	1674.7811	-0.0690	0	-.MTESFAQLFEESLK.E Oxidation (M) (Ions score 39)
2 - 14	764.8559	1527.6972	1527.7457	-0.0485	0	M.TESFAQLFEESLK.E (Ions score 77)

Error: try setting browser cache to automatic.

Error: try setting browser cache to automatic.

AC P0AG69; P02349; P77352; DT 21-JUL-1986, integrated into UniProtKB/Swiss-Prot. DT 20-DEC-2005, sequence version 1. DT 31-MAY-2011, entry version 39. DE RecName: Full=30S ribosomal protein S1; GN Name=rpsA; OrderedLocusNames=Z1257, ECs0994; OS Escherichia coli O157:H7. OC Bacteria; Proteobacteria; Gammaproteobacteria; Enterobacteriales; OC Enterobacteriaceae; Escherichia. OX NCBI_TaxID=83334; RN [1] RP NUCLEOTIDE SEQUENCE [LARGE SCALE GENOMIC DNA]. RC STRAIN=O157:H7 / EDL933 / ATCC 700927 / EHEC; RX MEDLINE=21074935; PubMed=11206551; DOI=10.1038/35054089; RA Perna N.T., Plunkett G. III, Burland V., Mau B., Glasner J.D., RA Rose D.J., Mayhew G.F., Evans P.S., Gregor J., Kirkpatrick H.A., RA Posfai G., Hackett J., Klink S., Boutin A., Shao Y., Miller L., RA Grotbeck E.J., Davis N.W., Lim A., Dimalanta E.T., Potamousis K., RA Apodaca J., Anantharaman T.S., Lin J., Yen G., Schwartz D.C., RA Welch R.A., Blattner F. R.; RT "Genome sequence of enterohaemorrhagic Escherichia coli O157:H7."; RL Nature 409:529-533(2001). RN [2] RP NUCLEOTIDE SEQUENCE [LARGE SCALE GENOMIC DNA]. RC STRAIN=O157:H7 / Sakai / RIMD 0509952 / EHEC; RX MEDLINE=21156231; PubMed=11258796; DOI=10.1093/dnares/8.1.11; RA Hayashi T., Makino K., Ohnishi M., Kurokawa K., Ishii K., Yokoyama K., RA Han C.-G., Ohtsubo E., Nakayama K., Murata T., Tanaka M., Tobe T., RA Iida T., Takami H., Honda T., Sasakawa C., Ogasawara N., Yasunaga T., RA Kuhara S., Shiba T., Hattori M., Shinagawa H.; RT "Complete genome sequence of enterohemorrhagic Escherichia coli RT O157:H7 and genomic comparison with a laboratory strain K-12."; RL DNA Res. 8:11-22(2001). CC -!- FUNCTION: Binds mRNA; thus facilitating recognition of the CC initiation point. It is needed to translate mRNA with a short CC Shine-Dalgarno (SD) purine-rich sequence (By similarity). CC -!- SUBUNIT: Part of the 30S ribosomal subunit. Some nascent CC polypeptide chains are able to cross-link to this protein in situ. CC Can be cross-linked to mRNA in the ribosome (By similarity). CC -!- PTM: Phosphorylated; probably on a serine (By similarity). CC -!- SIMILARITY: Belongs to the ribosomal protein S1P family. CC -!- SIMILARITY: Contains 6 S1 motif domains. CC --- CC Copyrighted by the UniProt Consortium, see http://www.uniprot.org/terms CC Distributed under the Creative Commons Attribution-NoDerivs License CC

-- DR EMBL; AE005174; AAG55396.1; -; Genomic_DNA. DR EMBL; BA000007; BAB34417.1; -; Genomic_DNA. DR PIR; B90753; B90753. DR PIR; H85616; H85616. DR RefSeq; NP_286786.1; NC_002655.2. DR RefSeq; NP_309021.1; NC_002695.1. DR ProteinModelPortal; P0AG69; -. DR SMR; P0AG69; 16-89, 103-180, 184-262, 270-350, 360-521. DR PRIDE; P0AG69; -. DR EnsemblBacteria; EBESCT00000026434; EBESCP00000025327; EBESCG00000025487. DR EnsemblBacteria; EBESCT00000056562; EBESCP00000054390; EBESCG00000055610. DR GeneID; 917736; -. DR GeneID; 958873; -. DR GenomeReviews; AE005174_GR; Z1257. DR GenomeReviews; BA000007_GR; ECs0994. DR KEGG; ece:Z1257; -. DR KEGG; ecs:ECs0994; -. DR GeneTree; EBGT00050000010054; -. DR HOGENOM; HBG635192; -. DR OMA; SITDYGA; -. DR ProtClustDB; PRK06299; -. DR BioCyc; ECOL83334:ECS0994-MONOMER; -. DR GO; GO:0005840; C:ribosome; IEA: UniProtKB-KW. DR GO; GO:0003723; F:RNA binding; IEA:UniProtKB-KW. DR GO; GO:0003735; F:structural constituent of ribosome; IEA:InterPro. DR GO; GO:0006412; P:translation; IEA:InterPro. DR InterPro; IPR012340; NA-bd_OB-fold. DR InterPro; IPR016027; NA-bd_OB-fold-like. DR InterPro; IPR018032; Rbsml-S1_synth_mod. DR InterPro; IPR003029; Rbsml_prot_S1_RNA-bd_dom. DR InterPro; IPR000110; Ribosomal_S1. DR InterPro; IPR022967; RNA-binding_domain_S1. DR Gene3D; G3DSA:2.40.50.140; OB_NA_bd_sub; 6. DR Pfam; PF00575; S1; 6. DR PRINTS; PR00681; RIBOSOMALS1. DR SMART; SM00316; S1; 6. DR SUPFAM; SSF50249; Nucleic_acid_OB; 6. DR TIGRFAMs; TIGR00717; RpsA; 1. DR PROSITE; PS50126; S1; 6. PE 3: Inferred from homology; KW Acetylation; Complete proteome; Phosphoprotein; Repeat; KW Ribonucleoprotein; Ribosomal protein; RNA-binding. FT CHAIN 1 557 30S ribosomal protein S1. FT /FTId=PRO_0000196034. FT DOMAIN 21 87 S1 motif 1. FT DOMAIN 105 171 S1 motif 2. FT DOMAIN 192 260 S1 motif 3. FT DOMAIN 277 347 S1 motif 4. FT DOMAIN 364 434 S1 motif 5. FT DOMAIN 451 520 S1 motif 6. FT MOD_RES 229 229 N6-acetyllysine (By similarity). FT MOD_RES 279 279 N6-acetyllysine (By similarity). FT MOD_RES 363 363 N6-acetyllysine (By similarity). SQ SEQUENCE 557 AA; 61158 MW; 0ABCDEB9E510C267 CRC64; MTESFAQLFE ESLKEIETRP GSIVRGVVVA IDKDVVLVDA GLKSESAIPA EQFKNAQGEL EIQVGDEVDV ALDAVEDGFG ETLLSREKAK RHEAWITLEK AYEDAETVTG VINGKVKGGF TVELNGIRAF LPGSLVDVRP VRDTLHLEGK ELEFKVIKLD QKRNNVVVSR RAVIESENSA ERDQLLENLQ EGMEVKGIVK NLTDYGAFVD LGGVDGLLHI TDMAWKRVKH PSEIVNVGDE ITVKVLKFDR ERTRVSLGLK QLGEDPWVAI AKRYPEGTKL TGRVTNLTDY GCFVEIEEGV EGLVHVSEMD WTNKNIHPSK VVNVGDVVEV MVLDIDEERR RISLGLKQCK ANPWQQFAET HNKGDRVEGK IKSITDFGIF IGLDGGIDGL VHLSDISWNV AGEEAVREYK KGDEIAAVVL QVDAERERIS LGVKQLAEDP FNNWVALNKK GAIVTGKVTA VDAKGATVEL ADGVEGYLRA SEASRDRVED ATLVLSVGDE VEAKFTGVDR KNRAISLSVR AKDEADEKDA IATVNKQEDA NFSNNAMAEA FKAAKGE

Mascot: http://www.matrixscience.com/

Mascot Search Results

Protein View

Match to: **EFTU_ACIBT** Score: **138**
Elongation factor Tu OS=Acinetobacter baumannii (strain ATCC 17978 / NCDC KC 755) GN=tuf1 PE=3 SV=2
Found in search of C:\Temp\mas4.tmp

Nominal mass (M_r): **43150**; Calculated pI value: **5.21**

NCBI BLAST search of EFTU_ACIBT against nr

Unformatted sequence string for pasting into other applications

Taxonomy: Acinetobacter baumannii ATCC 17978

Fixed modifications: Carbamidomethyl (C)
Variable modifications: Oxidation (M)
Cleavage by Trypsin: cuts C-term side of KR unless next residue is P
Sequence Coverage: **11%**

Matched peptides shown in **Bold Red**

```
  1 MAKAKFERNK PHVNVGTIGH VDHGKTTLTA AIATICAKTY GGEAKDYSQI
 51 DSAPEEKARG ITINTSHVEY DSPTRHYAHV DCPGHADYVK NMITGAAQMD
101 GAILVCAATD GPMPQTREHI LLSRQVGVPY IIVFLNKCDL VDDEELLELV
151 EMEVRELLST YDFPGDDTPV IRGSALAALN GEAGPYGEES VLALVAALDS
201 YIPEPERAID KAFLMPIEDV FSISGRGTVV TGRVEAGIIK VGEEVEIVGI
251 KDTVKTTVTG VEMFRKLLDE GRAGENCGIL LRGTKREEVQ RGQVLAKPGT
301 IKPHTKFDAE VYVLSKEEGG RHTPFLNGYR PQFYFRTTDV TGAIQLKEGV
351 EMVMPGDNVE MSVELIHPIA MDPGLRFAIR EGGRTVGAGV VAKVTA
```

Show predicted peptides also

Sort Peptides By ◉ Residue Number ○ Increasing Mass ○ Decreasing Mass

Start - End	Observed	Mr(expt)	Mr(calc)	Delta	Miss	Sequence
26 - 38	667.8793	1333.7440	1333.7275	0.0165	0	K.TTLTAAIATICAK.T (Ions score 55)

156 - 172	969.4954	1936.9762	1936.9418	0.0343	0	R.ELLSTYDFPGDDTPVIR.G	(Ions score 22)
156 - 172	969.9799	1937.9452	1936.9418	1.0033	0	R.ELLSTYDFPGDDTPVIR.G	(Ions score 33)
156 - 172	970.0003	1937.9860	1936.9418	1.0442	0	R.ELLSTYDFPGDDTPVIR.G	(Ions score 37)
212 - 226	841.4379	1680.8612	1680.8545	0.0066	0	K.AFLMPIEDVFSISGR.G	(Ions score 28)
212 - 226	849.4418	1696.8690	1696.8494	0.0196	0	K.AFLMPIEDVFSISGR.G	Oxidation (M) (Ions score 48)

Error: try setting browser cache to automatic.

Error: try setting browser cache to automatic.

AC A3M1F6; DT 20-MAY-2008, integrated into UniProtKB/Swiss-Prot. DT 20-MAY-2008, sequence version 2. DT 31-MAY-2011, entry version 36. DE RecName: Full=Elongation factor Tu; DE Short=EF-Tu; GN Name=tuf1; OrderedLocusNames=A1S_0279; GN and GN Name=tuf2; OrderedLocusNames=A1S_0869; OS Acinetobacter baumannii (strain ATCC 17978 / NCDC KC 755). OC Bacteria; Proteobacteria; Gammaproteobacteria; Pseudomonadales; OC Moraxellaceae; Acinetobacter; OC Acinetobacter calcoaceticus/baumannii complex. OX NCBI_TaxID=400667; RN [1] RP NUCLEOTIDE SEQUENCE [LARGE SCALE GENOMIC DNA]. RC STRAIN=ATCC 17978 / NCDC KC 755; RX PubMed=17344419; DOI=10.1101/gad.1510307; RA Smith M.G., Gianoulis T.A., Pukatzki S., Mekalanos J.J., Ornston L.N., RA Gerstein M., Snyder M.; RT "New insights into Acinetobacter baumannii pathogenesis revealed by RT high-density pyrosequencing and transposon mutagenesis."; RL Genes Dev. 21:601-614(2007). CC -!- FUNCTION: This protein promotes the GTP-dependent binding of CC aminoacyl-tRNA to the A-site of ribosomes during protein CC biosynthesis (By similarity). CC -!- SUBUNIT: Monomer (By similarity). CC -!- SUBCELLULAR LOCATION: Cytoplasm (By similarity). CC -!- SIMILARITY: Belongs to the GTP-binding elongation factor family. CC EF-Tu/EF-1A subfamily. CC -!- SEQUENCE CAUTION: CC Sequence=ABO11301.2; Type=Miscellaneous discrepancy; Note=This prediction is not full-length and may not be functional; CC --- CC Copyrighted by the UniProt Consortium, see http://www.uniprot.org/terms CC Distributed under the Creative Commons Attribution-NoDerivs License CC --- DR EMBL; CP000521; ABO10750.2; -; Genomic_DNA. DR EMBL; CP000521; ABO11301.2; ALT_SEQ; Genomic_DNA. DR RefSeq; YP_001083352.2; NC_009085.1. DR RefSeq; YP_001083903.2; NC_009085.1. DR ProteinModelPortal; A3M1F6; -. DR SMR; A3M1F6; 5-394. DR STRING; A3M1F6; -. DR GeneID; 4917140; -. DR GeneID; 4919042; -. DR GenomeReviews; CP000521_GR; A1S_0279. DR GenomeReviews; CP000521_GR; A1S_0869. DR KEGG; acb:A1S_0279; -. DR KEGG; acb: A1S_0869; -. DR eggNOG; COG0050; -. DR HOGENOM; HBG307581; -. DR GO; GO:0005737; C:cytoplasm; IEA:

UniProtKB-SubCell. DR GO; GO:0005525; F:GTP binding; IEA:UniProtKB-KW. DR GO; GO:0003924; F:GTPase activity; IEA:InterPro. DR GO; GO:0003746; F:translation elongation factor activity; IEA:UniProtKB-KW. DR HAMAP; MF_00118_B; EF_Tu_B; 1; -. DR InterPro; IPR000795; ProtSyn_GTP-bd. DR InterPro; IPR005225; Small_GTP-bd_dom. DR InterPro; IPR009001; Transl_elong_EF1A/Init_IF2_C. DR InterPro; IPR004161; Transl_elong_EFTu/EF1A_2. DR InterPro; IPR004541; Transl_elong_EFTu/EF1A_bac/org. DR InterPro; IPR004160; Transl_elong_EFTu/EF1A_C. DR InterPro; IPR009000; Transl_elong_init/rib_B-barrel. DR PANTHER; PTHR23115:SF31; Transl_elong_EFTu/EF1A_bac/org; 1. DR Pfam; PF00009; GTP_EFTU; 1. DR Pfam; PF03144; GTP_EFTU_D2; 1. DR Pfam; PF03143; GTP_EFTU_D3; 1. DR PRINTS; PR00315; ELONGATNFCT. DR SUPFAM; SSF50465; Elong_init_C; 1. DR SUPFAM; SSF50447; Translat_factor; 1. DR TIGRFAMs; TIGR00485; EF-Tu; 1. DR TIGRFAMs; TIGR00231; Small_GTP; 1. DR PROSITE; PS00301; EFACTOR_GTP; 1. PE 3: Inferred from homology; KW Complete proteome; Cytoplasm; Elongation factor; GTP-binding; KW Nucleotide-binding; Protein biosynthesis. FT CHAIN 1 396 Elongation factor Tu. FT /FTId=PRO_0000337301. FT NP_BIND 19 26 GTP (By similarity). FT NP_BIND 81 85 GTP (By similarity). FT NP_BIND 136 139 GTP (By similarity). SQ SEQUENCE 396 AA; 42892 MW; 56B9D9300174DF8A CRC64; MAKAKFERNK PHVNVGTIGH VDHGKTTLTA AIATICAKTY GGEAKDYSQI DSAPEEKARG ITINTSHVEY DSPTRHYAHV DCPGHADYVK NMITGAAQMD GAILVCAATD GPMPQTREHI LLSRQVGVPY IIVFLNKCDL VDDEELLELV EMEVRELLST YDFPGDDTPV IRGSALAALN GEAGPYGEES VLALVAALDS YIPEPERAID KAFLMPIEDV FSISGRGTVV TGRVEAGIIK VGEEVEIVGI KDTVKTTVTG VEMFRKLLDE GRAGENCGIL LRGTKREEVQ RGQVLAKPGT IKPHTKFDAE VYVLSKEEGG RHTPFLNGYR PQFYFRTTDV TGAIQLKEGV EMVMPGDNVE MSVELIHPIA MDPGLRFAIR EGGRTVGAGV VAKVTA

Mascot: http://www.matrixscience.com/

Mascot Search Results

Protein View

Match to: **EFTU_ACIAD** Score: **162**
Elongation factor Tu OS=Acinetobacter sp. (strain ADP1) GN=tuf1 PE=3 SV=1
Found in search of C:\Temp\mas4C.tmp

Nominal mass (M_r): **43213**; Calculated pI value: **5.20**

NCBI BLAST search of EFTU_ACIAD against nr
Unformatted sequence string for pasting into other applications

Taxonomy: Acinetobacter sp. ADP1

Fixed modifications: Carbamidomethyl (C)
Variable modifications: Oxidation (M)
Cleavage by Trypsin: cuts C-term side of KR unless next residue is P
Sequence Coverage: **8%**

Matched peptides shown in **Bold Red**

```
  1 MAKAKFERNK PHVNVGTIGH VDHGKTTLTA AIATICAKTY GGEAKDYSQI
 51 DSAPEEKARG ITINTSHVEY DSPIRHYAHV DCPGHADYVK NMITGAAQMD
101 GAILVCAATD GPMPQTREHI LLSRQVGVPY IVVFLNKCDL VDDEELLELV
151 EMEVRELLST YDFPGDDTPV IRGSALKALE GDAGQYGESS VLALVEALDT
201 YIPEPERAID KAFLMPIEDV FSISGRGTVV TGRVEAGIVK VGESVEIVGI
251 RDTQTTTVTG VEMFRKLLDE GRAGENCGVL LRGTKREDVQ RGQVLAKPGT
301 IKPHTKFDAE VYVLSKEEGG RHTPFLNGYR PQFYFRTTDV TGAIQLKEGV
351 EMVMPGDNVE MSVELIHPIA MDPGLRFAIR EGGRTVGAGV VAKVTA
```

Show predicted peptides also

Sort Peptides By ◉ Residue Number ○ Increasing Mass ○ Decreasing Mass

Start - End	Observed	Mr(expt)	Mr(calc)	Delta	Miss	Sequence	
156 - 172	969.4194	1936.8243	1936.9418	-0.1176	0	R.ELLSTYDFPGDDTPVIR.G	(Ions score 91)

212 - 226 841.3946 1680.7747 1680.8545 -0.0799 0 K.AFLMPIEDVFSISGR.G (Ions score 70)
212 - 226 849.3956 1696.7766 1696.8494 -0.0728 0 K.AFLMPIEDVFSISGR.G Oxidation (M) (Ions score 63)

Error: try setting browser cache to automatic.

Error: try setting browser cache to automatic.

AC Q6FF97; Q6FDS5; DT 20-MAY-2008, integrated into UniProtKB/Swiss-Prot. DT 19-JUL-2004, sequence version 1. DT 31-MAY-2011, entry version 55. DE RecName: Full=Elongation factor Tu; DE AltName: Full=EF-Tu 1; GN Name=tuf1; Synonyms=tufB; OrderedLocusNames=ACIAD0299; GN and GN Name=tuf2; Synonyms=tufA; OrderedLocusNames=ACIAD0885; OS Acinetobacter sp. (strain ADP1). OC Bacteria; Proteobacteria; Gammaproteobacteria; Pseudomonadales; OC Moraxellaceae; Acinetobacter. OX NCBI_TaxID=62977; RN [1] RP NUCLEOTIDE SEQUENCE [LARGE SCALE GENOMIC DNA]. RC STRAIN=ADP1; RX PubMed=15514110; DOI=10.1093/nar/gkh910; RA Barbe V., Vallenet D., Fonknechten N., Kreimeyer A., Oztas S., RA Labarre L., Cruveiller S., Robert C., Duprat S., Wincker P., RA Ornston L.N., Weissenbach J., Marliere P., Cohen G.N., Medigue C.; RT "Unique features revealed by the genome sequence of Acinetobacter sp. RT ADP1, a versatile and naturally transformation competent bacterium."; RL Nucleic Acids Res. 32:5766-5779(2004). CC -!- FUNCTION: This protein promotes the GTP-dependent binding of CC aminoacyl-tRNA to the A-site of ribosomes during protein CC biosynthesis (By similarity). CC -!- SUBUNIT: Monomer (By similarity). CC -!- SUBCELLULAR LOCATION: Cytoplasm (By similarity). CC -!- SIMILARITY: Belongs to the GTP-binding elongation factor family. CC EF-Tu/EF-1A subfamily. CC -!- SEQUENCE CAUTION: CC Sequence=CAG67783.1; Type=Erroneous initiation; CC --- CC Copyrighted by the UniProt Consortium, see http://www.uniprot.org/terms CC Distributed under the Creative Commons Attribution-NoDerivs License CC --- DR EMBL; CR543861; CAG67260.1; -; Genomic_DNA. DR EMBL; CR543861; CAG67783.1; ALT_INIT; Genomic_DNA. DR RefSeq; YP_045082.1; NC_005966.1. DR RefSeq; YP_045605.2; NC_005966.1. DR HSSP; P0A6N1; 2BVN. DR ProteinModelPortal; Q6FF97; -. DR SMR; Q6FF97; 5-394. DR STRING; Q6FF97; -. DR GeneID; 2880795; -. DR GeneID; 2880796; -. DR GenomeReviews; CR543861_GR; ACIAD0299. DR GenomeReviews; CR543861_GR; ACIAD0885. DR KEGG; aci:ACIAD0299; -. DR KEGG; aci:ACIAD0885; -. DR eggNOG; COG0050; -. DR HOGENOM; HBG307581; -. DR PhylomeDB; Q6FF97; -. DR BioCyc; ASP62977:ACIAD0299-MONOMER; -. DR GO; GO:0005737; C:cytoplasm; IEA:UniProtKB-SubCell. DR GO; GO:0005525; F:GTP binding; IEA:UniProtKB-KW. DR GO; GO:0003924; F:GTPase activity; IEA:InterPro. DR GO; GO:0003746; F:translation elongation factor activity; IEA:UniProtKB-KW. DR HAMAP; MF_00118_B; EF_Tu_B; 1; -. DR InterPro; IPR000795; ProtSyn_GTP-bd. DR InterPro; IPR005225;

Small_GTP-bd_dom. DR InterPro; IPR009001; Transl_elong_EF1A/Init_IF2_C. DR InterPro; IPR004161; Transl_elong_EFTu/EF1A_2. DR InterPro; IPR004541; Transl_elong_EFTu/EF1A_bac/org. DR InterPro; IPR004160; Transl_elong_EFTu/EF1A_C. DR InterPro; IPR009000; Transl_elong_init/rib_B-barrel. DR PANTHER; PTHR23115: SF31; Transl_elong_EFTu/EF1A_bac/org; 1. DR Pfam; PF00009; GTP_EFTU; 1. DR Pfam; PF03144; GTP_EFTU_D2; 1. DR Pfam; PF03143; GTP_EFTU_D3; 1. DR PRINTS; PR00315; ELONGATNFCT. DR SUPFAM; SSF50465; Elong_init_C; 1. DR SUPFAM; SSF50447; Translat_factor; 1. DR TIGRFAMs; TIGR00485; EF-Tu; 1. DR TIGRFAMs; TIGR00231; Small_GTP; 1. DR PROSITE; PS00301; EFACTOR_GTP; 1. PE 3: Inferred from homology; KW Complete proteome; Cytoplasm; Elongation factor; GTP-binding; KW Nucleotide-binding; Protein biosynthesis. FT CHAIN 1 396 Elongation factor Tu. FT /FTId=PRO_0000337302. FT NP_BIND 19 26 GTP (By similarity). FT NP_BIND 81 85 GTP (By similarity). FT NP_BIND 136 139 GTP (By similarity). SQ SEQUENCE 396 AA; 42955 MW; 51C4442B84266E7B CRC64; MAKAKFERNK PHVNVGTIGH VDHGKTTLTA AIATICAKTY GGEAKDYSQI DSAPEEKARG ITINTSHVEY DSPIRHYAHV DCPGHADYVK NMITGAAQMD GAILVCAATD GPMPQTREHI LLSRQVGVPY IVVFLNKCDL VDDEELLELV EMEVRELLST YDFPGDDTPV IRGSALKALE GDAGQYGESS VLALVEALDT YIPEPERAID KAFLMPIEDV FSISGRGTVV TGRVEAGIVK VGESVEIVGI RDTQTTTVTG VEMFRKLLDE GRAGENCGVL LRGTKREDVQ RGQVLAKPGT IKPHTKFDAE VYVLSKEEGG RHTPFLNGYR PQFYFRTTDV TGAIQLKEGV EMVMPGDNVE MSVELIHPIA MDPGLRFAIR EGGRTVGAGV VAKVTA

Mascot: http://www.matrixscience.com/

Mascot Search Results

Protein View

Match to: **RPOA_ACIBC** Score: **199**
DNA-directed RNA polymerase subunit alpha OS=Acinetobacter baumannii (strain ACICU) GN=rpoA PE=3 SV=1
Found in search of C:\Temp\mas54.tmp

Nominal mass (M_r): **37295**; Calculated pI value: **5.10**

NCBI BLAST search of RPOA_ACIBC against nr
Unformatted sequence string for pasting into other applications

Taxonomy: Acinetobacter baumannii ACICU

Fixed modifications: Carbamidomethyl (C)
Variable modifications: Oxidation (M)
Cleavage by Trypsin: cuts C-term side of KR unless next residue is P
Sequence Coverage: **14%**

Matched peptides shown in **Bold Red**

```
  1 MTRTANEFLT PQAIKVEAVS GTSAKVILEP LERGFGHTLG NALRRILLSS
 51 LPGAAVVEVE IEGVEHEYST LEGLQQDIVE LLLNLKGLSI KLFDQNEAYL
101 TLEKQGPGDI TAADLRLPHN VEVVNPEHLI GTLSATGSLK MRLKVSQGRG
151 YETSDSRFPE GETRPVGRLQ LDASYSPIKR VSYTVENARV EQRTDLDKLV
201 IDLETNGTVD PEEAIRKAAT ILQQQIAIFV DLQKDQTPVA QEPREEVDPI
251 LLRPVDDLEL TVRSANCLKA ENIYYIGDLV QRTEVELLKT PNLGKKSLTE
301 IKDVLASKGL QLGMRLENWP PASLRMDDRF AYRSR
```

Show predicted peptides also

Sort Peptides By   ◉ Residue Number   ○ Increasing Mass   ○ Decreasing Mass

Start - End	Observed	Mr(expt)	Mr(calc)	Delta	Miss	Sequence
34 - 44	571.8351	1141.6557	1141.5992	0.0565	0	R.GFGHTLGNALR.R (Ions score 37)
92 - 104	792.4306	1582.8466	1582.7879	0.0587	0	K.LFDQNEAYLTLEK.Q (Ions score 42)

169 - 180	695.9186	1389.8227	1389.7616	0.0611	1	R.LQLDASYSPIKR.V	(Ions score 79)
270 - 282	777.4343	1552.8541	1552.7885	0.0655	0	K.AENIYYIGDLVQR.T	(Ions score 41)

Error: try setting browser cache to automatic.

Error: try setting browser cache to automatic.

AC B2HZ83; DT 24-MAR-2009, integrated into UniProtKB/Swiss-Prot. DT 10-JUN-2008, sequence version 1. DT 27-JUL-2011, entry version 21. DE RecName: Full=DNA-directed RNA polymerase subunit alpha; DE Short=RNAP subunit alpha; DE EC=2.7.7.6; DE AltName: Full=RNA polymerase subunit alpha; DE AltName: Full=Transcriptase subunit alpha; GN Name=rpoA; OrderedLocusNames=ACICU_03253; OS Acinetobacter baumannii (strain ACICU). OC Bacteria; Proteobacteria; Gammaproteobacteria; Pseudomonadales; OC Moraxellaceae; Acinetobacter; OC Acinetobacter calcoaceticus/baumannii complex. OX NCBI_TaxID=405416; RN [1] RP NUCLEOTIDE SEQUENCE [LARGE SCALE GENOMIC DNA]. RC STRAIN=ACICU; RX PubMed=18411315; DOI=10.1128/AAC.01643-07; RA Iacono M., Villa L., Fortini D., Bordoni R., Imperi F., Bonnal R.J., RA Sicheritz-Ponten T., De Bellis G., Visca P., Cassone A., Carattoli A.; RT "Whole-genome pyrosequencing of an epidemic multidrug-resistant RT Acinetobacter baumannii strain belonging to the European clone II RT group."; RL Antimicrob. Agents Chemother. 52:2616-2625(2008). CC -!- FUNCTION: DNA-dependent RNA polymerase catalyzes the transcription CC of DNA into RNA using the four ribonucleoside triphosphates as CC substrates (By similarity). CC -!- CATALYTIC ACTIVITY: Nucleoside triphosphate + RNA(n) = diphosphate CC + RNA(n+1). CC -!- SUBUNIT: Homodimer. The RNAP catalytic core consists of 2 alpha, 1 CC beta, 1 beta' and 1 omega subunit. When a sigma factor is CC associated with the core the holoenzyme is formed, which can CC initiate transcription (By similarity). CC -!- DOMAIN: The N-terminal domain is essential for RNAP assembly and CC basal transcription, whereas the C-terminal domain is involved in CC interaction with transcriptional regulators and with upstream CC promoter elements (By similarity). CC -!- SIMILARITY: Belongs to the RNA polymerase alpha chain family. CC --- CC Copyrighted by the UniProt Consortium, see http://www.uniprot.org/terms CC Distributed under the Creative Commons Attribution-NoDerivs License CC --- DR EMBL; CP000863; ACC58565.1; -; Genomic_DNA. DR RefSeq; YP_001847912.1; NC_010611.1. DR ProteinModelPortal; B2HZ83; -. DR SMR; B2HZ83; 1-233, 248-324. DR STRING; B2HZ83; -. DR GeneID; 6235189; -. DR GenomeReviews; CP000863_GR; ACICU_03253. DR KEGG; abc:ACICU_03253; -. DR HOGENOM; HBG430844; -. DR OMA; FGTTLGN; -. DR ProtClustDB; PRK05182; -. DR GO; GO:0003677; F:DNA binding; IEA:InterPro. DR GO; GO:0003899; F:DNA-directed RNA polymerase activity; IEA:UniProtKB-KW. DR GO; GO:0046983; F:protein dimerization activity;

IEA:InterPro. DR HAMAP; MF_00059; RNApol_bact_RpoA; 1; -. DR InterPro; IPR011261; DNA-dir_RNA_pol_dimersation. DR InterPro; IPR011262; DNA-dir_RNA_pol_insert. DR InterPro; IPR009025; DNA-dir_RNA_pol_RBP11-like. DR InterPro; IPR011263; DNA-dir_RNA_pol_RpoA/D/Rpb3. DR InterPro; IPR011260; RNAP_asu_C. DR InterPro; IPR011773; RpoA. DR Gene3D; G3DSA:2.170.120.12; RNAP_insert; 1. DR Pfam; PF01000; RNA_pol_A_bac; 1. DR Pfam; PF03118; RNA_pol_A_CTD; 1. DR Pfam; PF01193; RNA_pol_L; 1. DR ProDom; PD001179; RNAP_asu_C; 1. DR SMART; SM00662; RPOLD; 1. DR SUPFAM; SSF47789; RNAP_alpha_C; 1. DR SUPFAM; SSF56553; RNAP_insert; 1. DR SUPFAM; SSF55257; RNAP_RBP11-like; 1. DR TIGRFAMs; TIGR02027; RpoA; 1. PE 3: Inferred from homology; KW Complete proteome; DNA-directed RNA polymerase; KW Nucleotidyltransferase; Transcription; Transferase. FT CHAIN 1 335 DNA-directed RNA polymerase subunit FT alpha. FT /FTId=PRO_1000091918. FT REGION 1 233 Alpha N-terminal domain (alpha-NTD) (By FT similarity). FT REGION 247 335 Alpha C-terminal domain (alpha-CTD) (By FT similarity). SQ SEQUENCE 335 AA; 37260 MW; 070997D8F74DB888 CRC64; MTRTANEFLT PQAIKVEAVS GTSAKVILEP LERGFGHTLG NALRRILLSS LPGAAVVEVE IEGVEHEYST LEGLQQDIVE LLLNLKGLSI KLFDQNEAYL TLEKQGPGDI TAADLRLPHN VEVVNPEHLI GTLSATGSLK MRLKVSQGRG YETSDSRFPE GETRPVGRLQ LDASYSPIKR VSYTVENARV EQRTDLDKLV IDLETNGTVD PEEAIRKAAT ILQQQIAIFV DLQKDQTPVA QEPREEVDPI LLRPVDDLEL TVRSANCLKA ENIYYIGDLV QRTEVELLKT PNLGKKSLTE IKDVLASKGL QLGMRLENWP PASLRMDDRF AYRSR

Mascot: http://www.matrixscience.com/

Mascot Search Results

Protein View

Match to: **CH60_ACIB3** Score: **191**
60 kDa chaperonin OS=Acinetobacter baumannii (strain AB307-0294) GN=groL PE=3 SV=1
Found in search of C:\Temp\mas51.tmp

Nominal mass (M_r): **57245**; Calculated pI value: **4.92**

NCBI BLAST search of CH60_ACIB3 against nr
Unformatted sequence string for pasting into other applications

Taxonomy: Acinetobacter baumannii AB307-0294

Fixed modifications: Carbamidomethyl (C)
Variable modifications: Oxidation (M)
Cleavage by Trypsin: cuts C-term side of KR unless next residue is P
Sequence Coverage: **11%**

Matched peptides shown in **Bold Red**

```
  1 MSAKDVKFGD SARSKMIAGV NVLADAVKVT LGPKGRNVVI DRSFGAPHIT
 51 KDGVTVAKEI SLKDKFENMG AQLVREVSSK TNDIAGDGTT TATVLAQAIL
101 NEGIKSVTAG MNPMDLKRGI DIAVKTVVEN IRSIAKPADD FKAIEQVGSI
151 SANSDTTVGK LIAQAMEKVG KEGVITVEEG SGFEDALDVV EGMQFDRGYI
201 SPYFANKQDT LTAELENPFI LLVDKKISNI RELISVLEAV AKTGKPLLII
251 AEDVEGEALA TLVVNNMRGI IKVCAVKAPG FGDRRKAMLQ DIAILTGATV
301 ISEEVGMSLE QATLQDLGTA HKITVSKENT VIVDGAGDAA AIAERVQQIR
351 AQIEESTSEY DREKLQERVA KLAGGVAVIK IGAATEVEMK EKKDRVDDAL
401 HATRAAVEEG VVAGGGVALV RAVNALEGLK GANEDQTAGI NILRRAIEAP
451 LRQIVANAGD EPSVVINAVK NGEGNFGYNA ATGEYGDMLE MGILDPAKVT
501 RSALEHAASV AGLMLTTECM ITDIPEDKPA APDMGGMGGM GGMM
```

Show predicted peptides also

Sort Peptides By   ◉ Residue Number   ○ Increasing Mass   ○ Decreasing Mass

Start - End	Observed	Mr(expt)	Mr(calc)	Delta	Miss	Sequence
81 - 105	1244.6432	2487.2718	2486.2864	0.9854	0	K.TNDIAGDGTTTATVLAQAILNEGIK.S (Ions score 98)
81 - 105	830.1091	2487.3055	2486.2864	1.0191	0	K.TNDIAGDGTTTATVLAQAILNEGIK.S (Ions score 79)
172 - 197	949.1170	2844.3292	2843.2808	1.0484	0	K.EGVITVEEGSGFEDALDVVEGMQFDR.G Oxidation (M) (Ions score 52)
232 - 242	586.8663	1171.7180	1170.6860	1.0320	0	R.ELISVLEAVAK.T (Ions score 40)

Error: try setting browser cache to automatic.

Error: try setting browser cache to automatic.

AC B7GY36; DT 14-APR-2009, integrated into UniProtKB/Swiss-Prot. DT 10-FEB-2009, sequence version 1. DT 27-JUL-2011, entry version 18. DE RecName: Full=60 kDa chaperonin; DE AltName: Full=GroEL protein; DE AltName: Full=Protein Cpn60; GN Name=groL; Synonyms=groEL; OrderedLocusNames=ABBFA_000812; OS Acinetobacter baumannii (strain AB307-0294). OC Bacteria; Proteobacteria; Gammaproteobacteria; Pseudomonadales; OC Moraxellaceae; Acinetobacter; OC Acinetobacter calcoaceticus/baumannii complex. OX NCBI_TaxID=557600; RN [1] RP NUCLEOTIDE SEQUENCE [LARGE SCALE GENOMIC DNA]. RC STRAIN=AB307-0294; RX PubMed=18931120; DOI=10.1128/JB.00834-08; RA Adams M.D., Goglin K., Molyneaux N., Hujer K.M., Lavender H., RA Jamison J.J., MacDonald I.J., Martin K.M., Russo T., Campagnari A.A., RA Hujer A.M., Bonomo R.A., Gill S.R.; RT "Comparative genome sequence analysis of multidrug-resistant RT Acinetobacter baumannii."; RL J. Bacteriol. 190:8053-8064(2008). CC -!- FUNCTION: Prevents misfolding and promotes the refolding and CC proper assembly of unfolded polypeptides generated under stress CC conditions (By similarity). CC -!- SUBUNIT: Oligomer of 14 subunits composed of two stacked rings of CC 7 subunits (By similarity). CC -!- SUBCELLULAR LOCATION: Cytoplasm (By similarity). CC -!- SIMILARITY: Belongs to the chaperonin (HSP60) family. CC --- CC Copyrighted by the UniProt Consortium, see http://www.uniprot.org/terms CC Distributed under the Creative Commons Attribution-NoDerivs License CC --- DR EMBL; CP001172; ACJ57160.1; -; Genomic_DNA. DR RefSeq; YP_002324726.1; NC_011595.1. DR ProteinModelPortal; B7GY36; -. DR SMR; B7GY36; 3-524. DR STRING; B7GY36; -. DR GeneID; 7059658; -. DR GenomeReviews; CP001172_GR; ABBFA_000812. DR KEGG; abb:ABBFA_000812; -. DR HOGENOM; HBG625289; -. DR OMA; DKPAAGM; -. DR ProtClustDB; PRK00013; -. DR GO; GO:0005737; C:cytoplasm; IEA:UniProtKB-SubCell. DR GO; GO:0005524; F:ATP

binding; IEA:UniProtKB-KW. DR GO; GO:0042026; P:protein refolding; IEA:InterPro. DR HAMAP; MF_00600; CH60; 1; -. DR InterPro; IPR018370; Chaperonin_Cpn60_CS. DR InterPro; IPR001844; Chaprnin_Cpn60. DR InterPro; IPR002423; Cpn60/TCP-1. DR PANTHER; PTHR11353; Cpn60/TCP-1; 1. DR Pfam; PF00118; Cpn60_TCP1; 1. DR PRINTS; PR00298; CHAPERONIN60. DR SUPFAM; SSF48592; GroEL-ATPase; 1. DR TIGRFAMs; TIGR02348; GroEL; 1. DR PROSITE; PS00296; CHAPERONINS_CPN60; 1. PE 3: Inferred from homology; KW ATP-binding; Chaperone; Complete proteome; Cytoplasm; KW Nucleotide-binding. FT CHAIN 1 544 60 kDa chaperonin. FT /FTId=PRO_1000129956. SQ SEQUENCE 544 AA; 57166 MW; 59547DF028D633D7 CRC64; MSAKDVKFGD SARSKMIAGV NVLADAVKVT LGPKGRNVVI DRSFGAPHIT KDGVTVAKEI SLKDKFENMG AQLVREVSSK TNDIAGDGTT TATVLAQAIL NEGIKSVTAG MNPMDLKRGI DIAVKTVVEN IRSIAKPADD FKAIEQVGSI SANSDTTVGK LIAQAMEKVG KEGVITVEEG SGFEDALDVV EGMQFDRGYI SPYFANKQDT LTAELENPFI LLVDKKISNI RELISVLEAV AKTGKPLLII AEDVEGEALA TLVVNNMRGI IKVCAVKAPG FGDRRKAMLQ DIAILTGATV ISEEVGMSLE QATLQDLGTA HKITVSKENT VIVDGAGDAA AIAERVQQIR AQIEESTSEY DREKLQERVA KLAGGVAVIK IGAATEVEMK EKKDRVDDAL HATRAAVEEG VVAGGGVALV RAVNALEGLK GANEDQTAGI NILRRAIEAP LRQIVANAGD EPSVVINAVK NGEGNFGYNA ATGEYGDMLE MGILDPAKVT RSALEHAASV AGLMLTTECM ITDIPEDKPA APDMGGMGGM GGMM

Mascot: http://www.matrixscience.com/

Mascot Search Results

Protein View

Match to: **gi|254480295** Score: **62**
TCP-1/cpn60 chaperonin family protein [marine gamma proteobacterium HTCC2148]
Found in search of C:\Temp\mas28.tmp

Nominal mass (M_r): **31707**; Calculated pI value: **4.74**

NCBI BLAST search of gi|254480295 against nr
Unformatted sequence string for pasting into other applications

Taxonomy: marine gamma proteobacterium HTCC2148
Links to retrieve other entries containing this sequence from NCBI Entrez:
gi|214039857 from marine gamma proteobacterium HTCC2148

Fixed modifications: Carbamidomethyl (C)
Variable modifications: Oxidation (M)
Cleavage by Trypsin: cuts C-term side of KR unless next residue is P
Sequence Coverage: **20%**

Matched peptides shown in **Bold Red**

```
  1 KASKPLIIIA EDVEGEALAT LVVNNMRGIV KVAACKAPGF GDRRKAMLQD
 51 IAILTGGTVI SEEVGMDLES ATLEHLGSAK RMTMDKDNST IIDGAGDAAA
101 IHARVGEIRT QIENTSSDYD REKLQERVAK LAGGVAVIKV GAATEIEMKE
151 KKARVEDALH ATRAAVEEGV VAGGGVALVR AIADIAELKG DNEDQNHGIA
201 AALRAMEGPL RQIVGNAGDE ASVVLDKIRQ GEGNYGYNAA TGEYGDMIEM
251 GILDPAKVTR TALQAAGSVA ALMITTEVMV ADSPDDGAGG APAMPDMGGM
301 GGMGGMM
```

Show predicted peptides also

Sort Peptides By ◉ Residue Number ○ Increasing Mass ○ Decreasing Mass

Start - End	Observed	Mr(expt)	Mr(calc)	Delta	Miss	Sequence

2 - 27 928.5124 2782.5152 2781.4946 1.0206 0 K.ASKPLIIIAEDVEGEALATLVVNNMR.G
Oxidation (M) (Ions score 41)

46 - 81 1263.6455 3787.9146 3786.9019 1.0126 1 K.AMLQDIAILTGGTVISEEVGMDLESATLEHLGSAKR.
M 2 Oxidation (M) (Ions score 21)

Error: try setting browser cache to automatic.

Error: try setting browser cache to automatic.

LOCUS ZP_05093543 307 aa linear BCT 09-JUN-2010 DEFINITION TCP-1/cpn60 chaperonin family protein [marine gamma proteobacterium HTCC2148]. ACCESSION ZP_05093543 VERSION ZP_05093543.1 GI:254480295 DBLINK Project: 55363 DBSOURCE REFSEQ: accession NZ_DS999222.1 KEYWORDS . SOURCE marine gamma proteobacterium HTCC2148 ORGANISM marine gamma proteobacterium HTCC2148 Bacteria; Proteobacteria; Gammaproteobacteria; OMG group; OM60 clade. REFERENCE 1 (residues 1 to 307) AUTHORS Amann,R., Fuchs,B., Giovannoni,S.J., Ferriera,S., Johnson,J., Kravitz,S., Beeson,K., Sutton,G., Rogers,Y.-H., Friedman,R., Frazier,M. and Venter,J.C. TITLE Direct Submission JOURNAL Submitted (16-JUL-2008) J. Craig Venter Institute, 9704 Medical Center Drive, Rockville, MD 20850, USA COMMENT PREDICTED REFSEQ: This record has not been reviewed and the function is unknown. The reference sequence was derived from EEB80516. COMPLETENESS: incomplete on the amino end. Method: conceptual translation. FEATURES Location/Qualifiers source 1..307 /organism="marine gamma proteobacterium HTCC2148" /strain="HTCC2148" /isolation_source="10 m water depth from station NH15" / db_xref="taxon:247634" /country="USA: Oregon Coast" Protein <1..307 /product="TCP-1/cpn60 chaperonin family protein" Region <1..282 /region_name="chaperonin_like" /note="chaperonin_like superfamily. Chaperonins are involved in productive folding of proteins. They share a common general morphology, a double toroid of 2 stacked rings, each composed of 7-9 subunits. There are 2 main chaperonin groups. The symmetry of type...; cl02777" /db_xref="CDD:194437" CDS 1..307 /locus_tag="GPB2148_3334" / coded_by="NZ_DS999222.1:<1..926" /note="[O] COG0459 Chaperonin GroEL (HSP60 family)" /codon_start=3 / transl_table=11

Mascot: http://www.matrixscience.com/

Mascot Search Results

Protein View

Match to: **gi|169633901** Score: **236**
putative lipoprotein [Acinetobacter baumannii SDF]
Found in search of C:\Temp\mas7F.tmp

Nominal mass (M_r): **16390**; Calculated pI value: **4.79**

NCBI BLAST search of gi|169633901 against nr
Unformatted sequence string for pasting into other applications

Taxonomy: Acinetobacter baumannii SDF
Links to retrieve other entries containing this sequence from NCBI Entrez:
gi|169152693 from Acinetobacter baumannii

Fixed modifications: Carbamidomethyl (C)
Variable modifications: Oxidation (M)
Cleavage by Trypsin: cuts C-term side of KR unless next residue is P
Sequence Coverage: **36%**

Matched peptides shown in **Bold Red**

```
  1 MQQSRNKHEN KTETTSDAST PVQTAQSNNN EAVDTAHTAE NSLDWDGKYK
 51 GTLPCADCEG IKTELELKDD KTYELTETYL GKGDANPFET HGKFTFDKDN
101 TSVITLDDKA QNRKFFIGEN TATALDMEGK KVEGSLAEHY VLKKED
```

Show predicted peptides also

Sort Peptides By ◉ Residue Number ○ Increasing Mass ○ Decreasing Mass

Start - End	Observed	Mr(expt)	Mr(calc)	Delta	Miss	Sequence
69 - 82	838.4227	1674.8309	1674.7988	0.0321	1	K.DDKTYELTETYLGK.G (Ions score 58)
72 - 82	659.3732	1316.7318	1316.6500	0.0818	0	K.TYELTETYLGK.G (Ions score 48)
83 - 93	586.7891	1171.5636	1171.5258	0.0378	0	K.GDANPFETHGK.F (Ions score 28)
115 - 130	880.4000	1758.7854	1758.8134	-0.0280	0	K.FFIGENTATALDMEGK.K Oxidation (M)

(Ions score 53)

131 - 143 737.4225 1472.8304 1471.8035 1.0269 1 K.KVEGSLAEHYVLK.K (Ions score 51)

Error: try setting browser cache to automatic.

Error: try setting browser cache to automatic.

LOCUS YP_001707637 146 aa linear BCT 17-MAY-2011 DEFINITION lipoprotein [Acinetobacter baumannii SDF]. ACCESSION YP_001707637 VERSION YP_001707637.1 GI:169633901 DBLINK Project: 61601 DBSOURCE REFSEQ: accession NC_010400.1 KEYWORDS . SOURCE Acinetobacter baumannii SDF ORGANISM Acinetobacter baumannii SDF Bacteria; Proteobacteria; Gammaproteobacteria; Pseudomonadales; Moraxellaceae; Acinetobacter; Acinetobacter calcoaceticus/baumannii complex. REFERENCE 1 (residues 1 to 146) AUTHORS Vallenet,D., Nordmann,P., Barbe,V., Poirel,L., Mangenot,S., Bataille,E., Dossat,C., Gas,S., Kreimeyer,A., Lenoble,P., Oztas,S., Poulain,J., Segurens,B., Robert,C., Abergel,C., Claverie,J.M., Raoult,D., Medigue,C., Weissenbach,J. and Cruveiller,S. TITLE Comparative analysis of Acinetobacters: three genomes for three lifestyles JOURNAL PLoS ONE 3 (3), E1805 (2008) PUBMED 18350144 REMARK Publication Status: Online-Only REFERENCE 2 (residues 1 to 146) CONSRTM NCBI Genome Project TITLE Direct Submission JOURNAL Submitted (04-MAR-2008) National Center for Biotechnology Information, NIH, Bethesda, MD 20894, USA REFERENCE 3 (residues 1 to 146) AUTHORS Genoscope -,C.E.A. TITLE Direct Submission JOURNAL Submitted (25-FEB-2008) Genoscope - Centre National de Sequencage : BP 191 91006 EVRY cedex - FRANCE (E-mail : seqref@genoscope. cns.fr - Web : www.genoscope.cns.fr) COMMENT PROVISIONAL REFSEQ: This record has not yet been subject to final NCBI review. The reference sequence was derived from CAP01694. Annotation data relative to BLAST similarities, COG assignations, enzymatic function prediction (PRIAM software), TMHMM and SignalP predictions, and synteny results (Syntonizer software) are available in BaumannoScope database via the MaGe annotation system http://www.genoscope.cns.fr/agc/mage/baumannoscope. Method: conceptual translation. FEATURES Location/Qualifiers source 1..146 /organism="Acinetobacter baumannii SDF" /strain="SDF" /db_xref="taxon:509170" Protein 1..146 /product="lipoprotein" /function="1.6.10 : lipoprotein" /calculated_mol_wt=16155 Region 49..135 /region_name="NlpE" /note="Uncharacterized lipoprotein NlpE involved in copper resistance; cl01138" /db_xref="CDD:194049" CDS 1..146 /locus_tag="ABSDF2382" /coded_by="NC_010400.1:2203755..2204195" /inference="ab initio prediction:AMIGene:2.0" /note="Evidence 3 : Function proposed based on presence of conserved amino acid motif, structural feature or limited homology; Product type pm : membrane component" /transl_table=11 /db_xref="GeneID:5985564"

Mascot: http://www.matrixscience.com/

Mascot Search Results

Protein View

Match to: **gi|56131242** Score: **283**
carbapenem-associated resistance protein precursor [Acinetobacter baumannii]
Found in search of C:\Temp\masB.tmp

Nominal mass (M_r): **26403**; Calculated pI value: **4.79**

NCBI BLAST search of gi|56131242 against nr
Unformatted sequence string for pasting into other applications

Taxonomy: Acinetobacter baumannii
Links to retrieve other entries containing this sequence from NCBI Entrez:
gi|224015984 from Acinetobacter baumannii
gi|224015988 from Acinetobacter baumannii

Fixed modifications: Carbamidomethyl (C)
Variable modifications: Oxidation (M)
Cleavage by Trypsin: cuts C-term side of KR unless next residue is P
Sequence Coverage: **19%**

Matched peptides shown in **Bold Red**

```
  1 MKVLRVLVTT TALLAAGAAM ADEAVVHDSY AFDKNQLIPV GARAEVGTTG
 51 YGGALLWQAN PYVGLALGYN GGDISWSDDL SINGTKYDMD MDNKLAYLNA
101 EIRPWGASTN PWAQGLYVAA GAAYVDNQYD LTKNVGTNAS VEIDGNRFNG
151 GANGVSIAGN LKYDNDIAPY IGFGFAPKFS KNWGVFGEVG AYYSGNPKVS
201 LASNNDALIG SDGRTLGKTL DDQERKIAND DKYKWLPVGK VGVNFYW
```

Show predicted peptides also

Sort Peptides By (•) Residue Number ( ) Increasing Mass ( ) Decreasing Mass

Start - End	Observed	Mr(expt)	Mr(calc)	Delta	Miss	Sequence	
163 - 178	894.9410	1787.8675	1786.8566	1.0109	0	K.YDNDIAPYIGFGFAPK.F	(Ions score 79)

182 - 198	922.9259	1843.8373	1843.8530	-0.0156	0	K.NWGVFGEVGAYYSGNPK.V	(Ions score 100)
199 - 214	794.9258	1587.8371	1587.7852	0.0518	0	K.VSLASNNDALIGSDGR.T	(Ions score 104)

Error: try setting browser cache to automatic.

Error: try setting browser cache to automatic.

LOCUS AAV80243 247 aa linear BCT 28-MAR-2005 DEFINITION carbapenem-associated resistance protein precursor [Acinetobacter baumannii]. ACCESSION AAV80243 VERSION AAV80243.1 GI:56131242 DBSOURCE accession AY684798.1 KEYWORDS . SOURCE Acinetobacter baumannii ORGANISM Acinetobacter baumannii Bacteria; Proteobacteria; Gammaproteobacteria; Pseudomonadales; Moraxellaceae; Acinetobacter; Acinetobacter calcoaceticus/baumannii complex. REFERENCE 1 (residues 1 to 247) AUTHORS Mussi,M.A., Limansky,A.S. and Viale,A.M. TITLE Acquisition of resistance to carbapenems in multidrug-resistant clinical strains of Acinetobacter baumannii: natural insertional inactivation of a gene encoding a member of a novel family of beta-barrel outer membrane proteins JOURNAL Antimicrob. Agents Chemother. 49 (4), 1432-1440 (2005) PUBMED 15793123 REFERENCE 2 (residues 1 to 247) AUTHORS Mussi,M.A., Limansky,A.S. and Viale,A.M. TITLE Direct Submission JOURNAL Submitted (13-JUL-2004) Microbiology, IBR, Suipacha 531, Rosario, Santa Fe 2000, Argentina COMMENT Method: conceptual translation supplied by author. FEATURES Location/Qualifiers source 1..247 / organism="Acinetobacter baumannii" /isolate="Ab244" /db_xref="taxon:470" Protein 1..247 / product="carbapenem-associated resistance protein precursor" /name="outer membrane protein" sig_peptide 1..21 mat_peptide 22..247 /product="carbapenem-associated resistance protein" CDS 1..247 /gene="carO" / coded_by="AY684798.1:2134..2877" /note="associated with carbapenem resistance; heat-modifiable protein" / transl_table=11

Mascot: http://www.matrixscience.com/

Mascot Search Results

Protein View

Match to: **gi|56131242** Score: **139**
carbapenem-associated resistance protein precursor [Acinetobacter baumannii]
Found in search of C:\Temp\mas9.tmp

Nominal mass (M_r): **26403**; Calculated pI value: **4.79**

NCBI BLAST search of gi|56131242 against nr
Unformatted sequence string for pasting into other applications

Taxonomy: Acinetobacter baumannii
Links to retrieve other entries containing this sequence from NCBI Entrez:
gi|224015984 from Acinetobacter baumannii
gi|224015988 from Acinetobacter baumannii

Fixed modifications: Carbamidomethyl (C)
Variable modifications: Oxidation (M)
Cleavage by Trypsin: cuts C-term side of KR unless next residue is P
Sequence Coverage: **13%**

Matched peptides shown in **Bold Red**

```
  1 MKVLRVLVTT TALLAAGAAM ADEAVVHDSY AFDKNQLIPV GARAEVGTTG
 51 YGGALLWQAN PYVGLALGYN GGDISWSDDL SINGTKYDMD MDNKLAYLNA
101 EIRPWGASTN PWAQGLYVAA GAAYVDNQYD LTKNVGTNAS VEIDGNRFNG
151 GANGVSIAGN LKYDNDIAPY IGFGFAPKFS KNWGVFGEVG AYYSGNPKVS
201 LASNNDALIG SDGRTLGKTL DDQERKIAND DKYKWLPVGK VGVNFYW
```

Show predicted peptides also

Sort Peptides By ◉ Residue Number ○ Increasing Mass ○ Decreasing Mass

Start - End	Observed	Mr(expt)	Mr(calc)	Delta	Miss	Sequence	
163 - 178	894.4644	1786.9143	1786.8566	0.0577	0	K.YDNDIAPYIGFGFAPK.F	(Ions score 40)

182 - 198	922.9743	1843.9341	1843.8530	0.0812	0	K.NWGVFGEVGAYYSGNPK.V	(Ions score 100)

Error: try setting browser cache to automatic.

Error: try setting browser cache to automatic.

LOCUS AAV80243 247 aa linear BCT 28-MAR-2005 DEFINITION carbapenem-associated resistance protein precursor [Acinetobacter baumannii]. ACCESSION AAV80243 VERSION AAV80243.1 GI:56131242 DBSOURCE accession AY684798.1 KEYWORDS . SOURCE Acinetobacter baumannii ORGANISM Acinetobacter baumannii Bacteria; Proteobacteria; Gammaproteobacteria; Pseudomonadales; Moraxellaceae; Acinetobacter; Acinetobacter calcoaceticus/baumannii complex. REFERENCE 1 (residues 1 to 247) AUTHORS Mussi,M.A., Limansky,A.S. and Viale,A.M. TITLE Acquisition of resistance to carbapenems in multidrug-resistant clinical strains of Acinetobacter baumannii: natural insertional inactivation of a gene encoding a member of a novel family of beta-barrel outer membrane proteins JOURNAL Antimicrob. Agents Chemother. 49 (4), 1432-1440 (2005) PUBMED 15793123 REFERENCE 2 (residues 1 to 247) AUTHORS Mussi,M.A., Limansky,A.S. and Viale,A.M. TITLE Direct Submission JOURNAL Submitted (13-JUL-2004) Microbiology, IBR, Suipacha 531, Rosario, Santa Fe 2000, Argentina COMMENT Method: conceptual translation supplied by author. FEATURES Location/Qualifiers source 1..247 /organism="Acinetobacter baumannii" /isolate="Ab244" /db_xref="taxon:470" Protein 1..247 /product="carbapenem-associated resistance protein precursor" /name="outer membrane protein" sig_peptide 1..21 mat_peptide 22..247 /product="carbapenem-associated resistance protein" CDS 1..247 /gene="carO" /coded_by="AY684798.1:2134..2877" /note="associated with carbapenem resistance; heat-modifiable protein" /transl_table=11

Mascot: http://www.matrixscience.com/

Mascot Search Results

Protein View

Match to: **gi|126641429** Score: **75**
surface antigen [Acinetobacter baumannii ATCC 17978]
Found in search of C:\Temp\mas67.tmp

Nominal mass (M_r): **11318**; Calculated pI value: **4.77**

NCBI BLAST search of gi|126641429 against nr
Unformatted sequence string for pasting into other applications

Taxonomy: Acinetobacter baumannii ATCC 17978

Fixed modifications: Carbamidomethyl (C)
Variable modifications: Oxidation (M)
Cleavage by Trypsin: cuts C-term side of KR unless next residue is P
Sequence Coverage: **11%**

Matched peptides shown in **Bold Red**

```
 1 MVAGDHNYSD SRWRESYLTR PYYQEAQLTT PDLDYDRDFS AAYELGHRAR
51 SESKEGTQFE DMEGSLQQKW EELKAESRLK WEHAKQAIKD AWDDM
```

Show predicted peptides also

Sort Peptides By ◉ Residue Number ○ Increasing Mass ○ Decreasing Mass

Start - End	Observed	Mr(expt)	Mr(calc)	Delta	Miss	Sequence	
38 - 48	633.3560	1264.6975	1264.5836	0.1139	0	R.DFSAAYELGHR.A	(Ions score 28)
38 - 48	633.3607	1264.7069	1264.5836	0.1233	0	R.DFSAAYELGHR.A	(Ions score 75)

Error: try setting browser cache to automatic.

Error: try setting browser cache to automatic.

LOCUS YP_001084413 95 aa linear BCT 29-MAY-2010 DEFINITION surface antigen [Acinetobacter baumannii ATCC 17978]. ACCESSION YP_001084413 VERSION YP_001084413.1 GI:126641429 DBLINK Project: 58731 DBSOURCE REFSEQ: accession NC_009085.1 KEYWORDS . SOURCE Acinetobacter baumannii ATCC 17978 ORGANISM Acinetobacter baumannii ATCC 17978 Bacteria; Proteobacteria; Gammaproteobacteria; Pseudomonadales; Moraxellaceae; Acinetobacter; Acinetobacter calcoaceticus/baumannii complex. REFERENCE 1 (residues 1 to 95) AUTHORS Smith, M.G., Gianoulis,T.A., Pukatzki,S., Mekalanos,J.J., Ornston,L.N., Gerstein,M. and Snyder,M. TITLE New insights into Acinetobacter baumannii pathogenesis revealed by high-density pyrosequencing and transposon mutagenesis JOURNAL Genes Dev. 21 (5), 601-614 (2007) PUBMED 17344419 REFERENCE 2 (residues 1 to 95) CONSRTM NCBI Genome Project TITLE Direct Submission JOURNAL Submitted (05-MAR-2007) National Center for Biotechnology Information, NIH, Bethesda, MD 20894, USA REFERENCE 3 (residues 1 to 95) AUTHORS Smith,M.G., Gianoulis,T.A., Pukatzki,S., Mekalanos,J.J., Ornston,L.N., Gerstein,M. and Snyder,M. TITLE Direct Submission JOURNAL Submitted (27-NOV-2006) Department of Molecular, Cellular, and Developmental Biology, Yale University, 266 Whitney Ave, New Haven, CT 06520, USA COMMENT PROVISIONAL REFSEQ: This record has not yet been subject to final NCBI review. The reference sequence was derived from ABO11811. Source DNA and bacteria available from Michael Snyder (michael.snyder@yale.edu). Method: conceptual translation. FEATURES Location/Qualifiers source 1..95 /organism="Acinetobacter baumannii ATCC 17978" /strain="ATCC 17978" / db_xref="ATCC:17978" /db_xref="taxon:400667" Protein 1..95 /product="surface antigen" / calculated_mol_wt=11194 CDS 1..95 /locus_tag="A1S_1383" /coded_by="complement (NC_009085.1:1622740..1623027)" /transl_table=11 /db_xref="GeneID:4919934"

**Mascot:** http://www.matrixscience.com/

Mascot Search Results

Protein View

Match to: **gi|126641429** Score: **69**
surface antigen [Acinetobacter baumannii ATCC 17978]
Found in search of C:\Temp\mas71.tmp

Nominal mass (M_r): **11318**; Calculated pI value: **4.77**

NCBI BLAST search of gi|126641429 against nr
Unformatted sequence string for pasting into other applications

Taxonomy: Acinetobacter baumannii ATCC 17978

Fixed modifications: Carbamidomethyl (C)
Variable modifications: Oxidation (M)
Cleavage by Trypsin: cuts C-term side of KR unless next residue is P
Sequence Coverage: **24%**

Matched peptides shown in **Bold Red**

1 MVAGDHNYSD SRWR**ESYLTR PYYQEAQLTT PDLDYDR**DFS AAYELGHRAR
51 SESKEGTQFE DMEGSLQQKW EELKAESRLK WEHAKQAIKD AWDDM

Show predicted peptides also

Sort Peptides By ◉ Residue Number ○ Increasing Mass ○ Decreasing Mass

Start - End	Observed	Mr(expt)	Mr(calc)	Delta	Miss	Sequence
15 - 37	946.8142	2837.4207	2836.3191	1.1016	0	R.ESYLTRPYYQEAQLTTPDLDYDR.D (Ions score 69)

Error: try setting browser cache to automatic.

Error: try setting browser cache to automatic.

LOCUS YP_001084413 95 aa linear BCT 29-MAY-2010 DEFINITION surface antigen [Acinetobacter baumannii ATCC 17978]. ACCESSION YP_001084413 VERSION YP_001084413.1 GI:126641429 DBLINK Project: 58731 DBSOURCE REFSEQ: accession NC_009085.1 KEYWORDS . SOURCE Acinetobacter baumannii ATCC 17978 ORGANISM Acinetobacter baumannii ATCC 17978 Bacteria; Proteobacteria; Gammaproteobacteria; Pseudomonadales; Moraxellaceae; Acinetobacter; Acinetobacter calcoaceticus/baumannii complex. REFERENCE 1 (residues 1 to 95) AUTHORS Smith, M.G., Gianoulis,T.A., Pukatzki,S., Mekalanos,J.J., Ornston,L.N., Gerstein,M. and Snyder,M. TITLE New insights into Acinetobacter baumannii pathogenesis revealed by high-density pyrosequencing and transposon mutagenesis JOURNAL Genes Dev. 21 (5), 601-614 (2007) PUBMED 17344419 REFERENCE 2 (residues 1 to 95) CONSRTM NCBI Genome Project TITLE Direct Submission JOURNAL Submitted (05-MAR-2007) National Center for Biotechnology Information, NIH, Bethesda, MD 20894, USA REFERENCE 3 (residues 1 to 95) AUTHORS Smith,M.G., Gianoulis,T.A., Pukatzki,S., Mekalanos,J.J., Ornston,L.N., Gerstein,M. and Snyder,M. TITLE Direct Submission JOURNAL Submitted (27-NOV-2006) Department of Molecular, Cellular, and Developmental Biology, Yale University, 266 Whitney Ave, New Haven, CT 06520, USA COMMENT PROVISIONAL REFSEQ: This record has not yet been subject to final NCBI review. The reference sequence was derived from ABO11811. Source DNA and bacteria available from Michael Snyder (michael.snyder@yale.edu). Method: conceptual translation. FEATURES Location/Qualifiers source 1..95 /organism="Acinetobacter baumannii ATCC 17978" /strain="ATCC 17978" /db_xref="ATCC:17978" /db_xref="taxon:400667" Protein 1..95 /product="surface antigen" /calculated_mol_wt=11194 CDS 1..95 /locus_tag="A1S_1383" /coded_by="complement (NC_009085.1:1622740..1623027)" /transl_table=11 /db_xref="GeneID:4919934"

Mascot: http://www.matrixscience.com/

Mascot Search Results

Protein View

Match to: **RRF_ACIBS** Score: **75**
Ribosome-recycling factor OS=Acinetobacter baumannii (strain SDF) GN=frr PE=3 SV=1
Found in search of C:\Temp\mas5F.tmp

Nominal mass (M_r): **20657**; Calculated pI value: **5.66**

NCBI BLAST search of RRF_ACIBS against nr
Unformatted sequence string for pasting into other applications

Taxonomy: Acinetobacter baumannii SDF

Fixed modifications: Carbamidomethyl (C)
Variable modifications: Oxidation (M)
Cleavage by Trypsin: cuts C-term side of KR unless next residue is P
Sequence Coverage: **8%**

Matched peptides shown in **Bold Red**

```
  1 MINDLKKDSE QRMLKTLESL EQGFAKVRTG RAHPSILNGV MVPYYGSDVP
 51 LNQVANVGVE DSRTLIVQPF ERTMVAAIDK AIRESDLGLN PITADSIRVP
101 LPALTEETRR DMQKIARSEA ENAKVAIRNI RRDVLGDIKA LLKEKEISED
151 DERRAGDDIQ KITDKYVAEV DKRLAAKEAE LMRV
```

Show predicted peptides also

Sort Peptides By ◉ Residue Number ○ Increasing Mass ○ Decreasing Mass

Start - End	Observed	Mr(expt)	Mr(calc)	Delta	Miss	Sequence
84 - 98	801.4292	1600.8438	1599.8104	1.0334	0	R.ESDLGLNPITADSIR.V (Ions score 75)

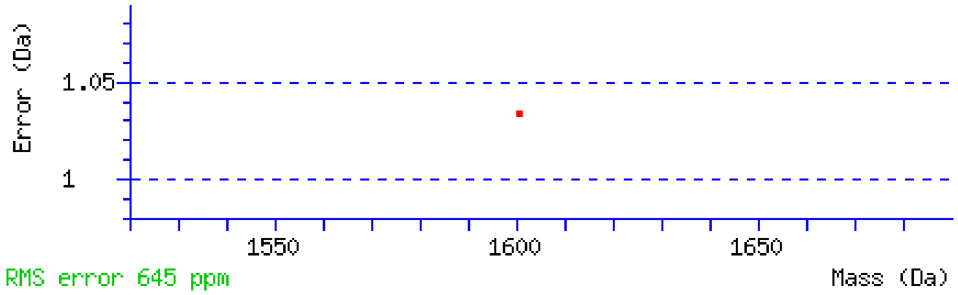

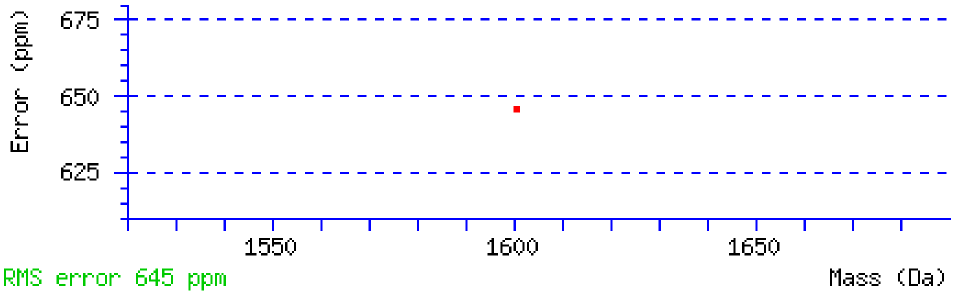

AC B0VMU5; DT 24-MAR-2009, integrated into UniProtKB/Swiss-Prot. DT 08-APR-2008, sequence version 1. DT 27-JUL-2011, entry version 26. DE RecName: Full=Ribosome-recycling factor; DE Short=RRF; DE AltName: Full=Ribosome-releasing factor; GN Name=frr; OrderedLocusNames=ABSDF1681; OS Acinetobacter baumannii (strain SDF). OC Bacteria; Proteobacteria; Gammaproteobacteria; Pseudomonadales; OC Moraxellaceae; Acinetobacter; OC Acinetobacter calcoaceticus/baumannii complex. OX NCBI_TaxID=509170; RN [1] RP NUCLEOTIDE SEQUENCE [LARGE SCALE GENOMIC DNA]. RC STRAIN=SDF; RX PubMed=18350144; DOI=10.1371/journal. pone.0001805; RA Vallenet D., Nordmann P., Barbe V., Poirel L., Mangenot S., RA Bataille E., Dossat C., Gas S., Kreimeyer A., Lenoble P., Oztas S., RA Poulain J., Segurens B., Robert C., Abergel C., Claverie J.-M., RA Raoult D., Medigue C., Weissenbach J., Cruveiller S.; RT "Comparative analysis of Acinetobacters: three genomes for three RT lifestyles."; RL PLoS ONE 3:E1805-E1805(2008). CC -!- FUNCTION: Responsible for the release of ribosomes from messenger CC RNA at the termination of protein biosynthesis. May increase the CC efficiency of translation by recycling ribosomes from one round of CC translation to another (By similarity). CC -!- SUBCELLULAR LOCATION: Cytoplasm (By similarity). CC -!- SIMILARITY: Belongs to the RRF family. CC --- CC Copyrighted by the UniProt Consortium, see http://www.uniprot.org/terms CC Distributed under the Creative Commons Attribution-NoDerivs License CC --- DR EMBL; CU468230; CAP01021.1; -; Genomic_DNA. DR RefSeq; YP_001707066.1; NC_010400.1. DR ProteinModelPortal; B0VMU5; -. DR SMR; B0VMU5; 1-184. DR STRING; B0VMU5; -. DR GeneID; 5987239; -. DR GenomeReviews; CU468230_GR; ABSDF1681. DR KEGG; abm:ABSDF1681; -. DR HOGENOM; HBG533975; -. DR OMA; YYGAETP; -. DR ProtClustDB; PRK00083; -. DR GO; GO:0005737; C:cytoplasm; IEA:UniProtKB-SubCell. DR GO; GO:0006412; P:translation; IEA:UniProtKB-KW. DR HAMAP; MF_00040; RRF; 1; -. DR InterPro; IPR002661; Ribosome_recyc_fac. DR InterPro; IPR023584; Ribosome_recyc_fac_dom. DR PANTHER; PTHR20982; Ribosome_recyc_fac; 1. DR Pfam; PF01765; RRF; 1. DR SUPFAM; SSF55194; Ribosome_recyc_fac; 1. DR TIGRFAMs; TIGR00496; Frr; 1. PE 3: Inferred from homology; KW Complete proteome; Cytoplasm; Protein biosynthesis. FT CHAIN 1 184 Ribosome-recycling factor. FT /FTId=PRO_1000090697. SQ SEQUENCE 184 AA; 20670 MW; 9D0B73BC15759FC7 CRC64; MINDLKKDSE QRMLKTLESL EQGFAKVRTG RAHPSILNGV MVPYYGSDVP LNQVANVGVE DSRTLIVQPF ERTMVAAIDK AIRESDLGLN PITADSIRVP LPALTEETRR DMQKIARSEA ENAKVAIRNI RRDVLGDIKA LLKEKEISED DERRAGDDIQ KITDKYVAEV DKRLAAKEAE LMRV

Mascot: http://www.matrixscience.com/

Mascot Search Results

Protein View

Match to: **gi|239500971** Score: **53**
Outer membrane protein W [Acinetobacter baumannii AB900]
Found in search of C:\Temp\mas79.tmp

Nominal mass (M_r): **21239**; Calculated pI value: **5.56**

NCBI BLAST search of gi|239500971 against nr
Unformatted sequence string for pasting into other applications

Taxonomy: Acinetobacter baumannii AB900

Fixed modifications: Carbamidomethyl (C)
Variable modifications: Oxidation (M)
Cleavage by Trypsin: cuts C-term side of KR unless next residue is P
Sequence Coverage: **10%**

Matched peptides shown in **Bold Red**

```
  1 MFKKVLVVAL MGVSSFTFAG NWQVKFGGSV IAPSEDTTTP LGVVKADHEY
 51 AFTPSVEYFF GQSPFSAELL LATPINHDVL LDGQKVARIK QLPPTITAKY
101 HFKNSTRFTP YIGIGATAFI PWDEQGVADK VKEDFGVAGQ VGFNFQPADA
151 KNWGVFVDVR YADISPEVTL TNGAKFDLDI NPFVYTLGYS YKF
```

Show predicted peptides also

Sort Peptides By ◉ Residue Number ○ Increasing Mass ○ Decreasing Mass

Start - End	Observed	Mr(expt)	Mr(calc)	Delta	Miss	Sequence
26 - 45	988.0298	1974.0451	1974.0310	0.0140	0	K.FGGSVIAPSEDTTTPLGVVK.A (Ions score 53)

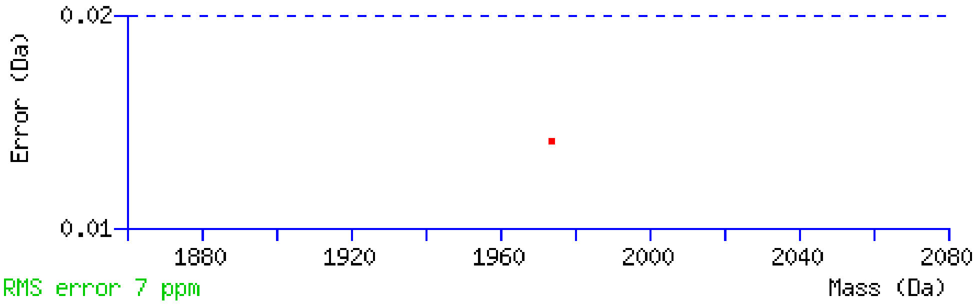

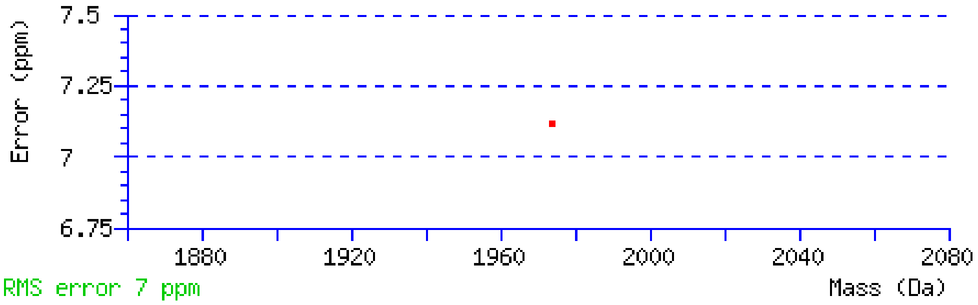

LOCUS ZP_04660281 193 aa linear BCT 10-DEC-2010 DEFINITION Outer membrane protein W [Acinetobacter baumannii AB900]. ACCESSION ZP_04660281 VERSION ZP_04660281.1 GI:239500971 DBSOURCE REFSEQ: accession NZ_ABXK01000001.1 KEYWORDS . SOURCE Acinetobacter baumannii AB900 ORGANISM Acinetobacter baumannii AB900 Bacteria; Proteobacteria; Gammaproteobacteria; Pseudomonadales; Moraxellaceae; Acinetobacter; Acinetobacter calcoaceticus/baumannii complex. COMMENT WGS REFSEQ: This record is provided to represent a collection of whole genome shotgun sequences. The reference sequence was derived from ABXK01000001. Method: conceptual translation. FEATURES Location/Qualifiers source 1..193 /organism="Acinetobacter baumannii AB900" /strain="AB900" /db_xref="taxon:557601" Protein 1..193 /product="Outer membrane protein W" /calculated_mol_wt=21121 Region 19..193 /region_name="Surface_Ag_2" /note="Surface antigen; cl01155" /db_xref="CDD:198629" CDS 1..193 /locus_tag="AbauAB_010100001530" /coded_by="complement (NZ_ABXK01000001.1:331995..332576)" /note="COG3047 Outer membrane protein W" /transl_table=11 /db_xref="CDD:112722"

Mascot: http://www.matrixscience.com/
