# Supplementary material for: Comparative Proteomics of Inner Membrane Fraction from Carbapenem-Resistant Acinetobacter baumannii with a Reference Strain
Source: PLoS One. 2012 Jun 26;7(6):e39451. doi: 10.1371/journal.pone.0039451 (PMC3383706; doi:10.1371/journal.pone.0039451)
Supplement: Table S1 — Differentially expressed proteins identified in inner membrane fraction of RS122. (PDF) [file pone.0039451.s005.pdf]

Table S1. Identification of differentially expressed proteins in inner membrane fraction of *Acinetobacter baumannii* intermediate resistant strain, RS122 with reference to ATCC 19606. Differential expression is shown as fold change (minimum 2 fold, p-value e0.05)

Master No.	Fold change	Mw kDa	pI	Identification protein	Score	Functions
510	2.98	19.4	4.98	ATP synthase subunit delta of *Acinetobacter baumannii*	194	Subunit delta does not bear the catalytic high-affinity ATP-binding sites but require for function of ATP Synthase.
620	2.95	18.6	5.72	DNA protection during starvation protein	166	It protects DNA from oxidative damage by sequestering intracellular Fe^{2+} ion.
