# Supplementary material for: Comparative Proteomics of Inner Membrane Fraction from Carbapenem-Resistant Acinetobacter baumannii with a Reference Strain
Source: PLoS One. 2012 Jun 26;7(6):e39451. doi: 10.1371/journal.pone.0039451 (PMC3383706; doi:10.1371/journal.pone.0039451)
Supplement: Table S2 — Differentially expressed proteins identified in inner membrane fraction of RS259. (PDF) [file pone.0039451.s006.pdf]

Table S2. Identification of differentially expressed proteins in inner membrane fraction of *Acinetobacter baumannii* low resistant strain, RS259 with reference to ATCC 19606. Differential expression is shown as fold change (minimum 2 fold, p-value e0.05).

Master No.	Fold change	Mw kDa	pI	Identification and Accession No.	Score	Role in Resistance
806	3.05	43.1	5.21	Elongation factor Tu OS=Acinetobacter baumannii, EFTU_ACIBT	74	Associated with efficient translation.
1003	3.01	51.5	5.43	3-isopropyl malate dehydratase large subunit OS=Acinetobacter baumannii LEUC_ACIBT	40	Involved in amino-acid biosynthesis, branched-chain amino acid biosynthesis, leucine biosynthesis.
